# Tailored biosynthesis of gibberellin plant hormones in yeast

**DOI:** 10.1016/j.ymben.2021.03.010

**Published:** 2021-07

**Authors:** Kanchana R. Kildegaard, Jonathan A. Arnesen, Belén Adiego-Pérez, Daniela Rago, Mette Kristensen, Andreas K. Klitgaard, Esben H. Hansen, Jørgen Hansen, Irina Borodina

**Affiliations:** a The Novo Nordisk Foundation Center for Biosustainability, Technical University of Denmark, Kemitorvet 220, 2800, Kgs. Lyngby, Denmark; b River Stone Biotech ApS, Fruebjergvej 3, 2100, København Ø, Denmark

**Keywords:** Gibberellins, Plant growth hormones, Isoprenoids, Oleaginous yeast, *Yarrowia lipolytica*

## Abstract

The application of small amounts of natural plant growth hormones, such as gibberellins (GAs), can increase the productivity and quality of many vegetable and fruit crops. However, gibberellin growth hormones usage is limited by the high cost of their production, which is currently based on fermentation of a natural fungal producer *Fusarium fujikuroi* that produces a mix of several GAs. We explored the potential of the oleaginous yeast *Yarrowia lipolytica* to produce specific profiles of GAs. Firstly, the production of the GA-precursor *ent*-kaurenoic acid (KA) at 3.75 mg/L was achieved by expression of biosynthetic enzymes from the plant *Arabidopsis thaliana* and upregulation of the mevalonate (MVA) pathway.

We then built a GA_4_-producing strain by extending the GA-biosynthetic pathway and upregulating the MVA-pathway further, resulting in 17.29 mg/L GA_4_. Additional expression of the *F. fujikoroi* GA-biosynthetic enzymes resulted in the production of GA_7_ (trace amounts) and GA_3_ (2.93 mg/L). Lastly, through protein engineering and the expression of additional KA-biosynthetic genes, we increased the GA_3_-production 4.4-fold resulting in 12.81 mg/L. The developed system presents a promising resource for the recombinant production of specific gibberellins, identifying bottlenecks in GA biosynthesis, and discovering new GA biosynthetic genes.

**Classification:**

Biological Sciences, Applied Biological Sciences.

## Highlights

• A complete biosynthetic pathway towards gibberellins was reconstructed in a microbial host
• The pathway towards *ent*-kaurenoic acid consisted of *Arabidopsis thaliana* enzymes
• The pathway from *ent*-kaurenoic acid to gibberellins GA_3_, GA_4_ and GA_7_ consisted of *Fusarium fujikuroi* enzymes
• *Y. lipolytica* expressed 14 heterologous genes for gibberellins biosynthesis and had 5 genome edits for improved mevalonate flux
• The strains produced up to 12 mg/L of GA_3_ and up to 17 mg/L GA_4_ in small-scale cultivations

## 1 Introduction

Gibberellins (GAs) are diterpene phytohormones involved in plant developmental processes like seed germination, pollen maturation, stem elongation, flower formation, and fruit development (Kato, 1955; Lang, 1956; Harrington et al., 1957; Kahn et al., 1957; Wittwer et al., 1957). Of the at least 126 known gibberellins, some are bioactive while others are inert side-products or biosynthetic intermediates (MacMillan, 2001). Bioactive gibberellins include GA_3_ (gibberellic acid), GA_4_, and GA_7_; they are used in commercial agricultural products, either pure or as mixtures (Camara et al., 2018). Spraying with gibberellins increases the size of seedless grapes, blueberries, and pears (Casanova et al., 2009; Ito et al., 2016; Zang et al., 2016), as well as the salt stress tolerance of soybeans, maize, and sugarcane (Tuna et al., 2008; Hamayun et al., 2010; Shomeili et al., 2011). Valent Biosciences, a commercial provider of GA-products, lists more than 40 plant species that can be enhanced by gibberellins (Rademacher, 2015). Gibberellins are also applied in beer brewing to enhance the malting process (MacLeod and Millar, 1962). According to some sources, the estimated global market size for gibberellins is USD 548.9 million as of 2016 (Gibberellins Market | Industry Analysis Report, 2016).

**Fig. 1:**
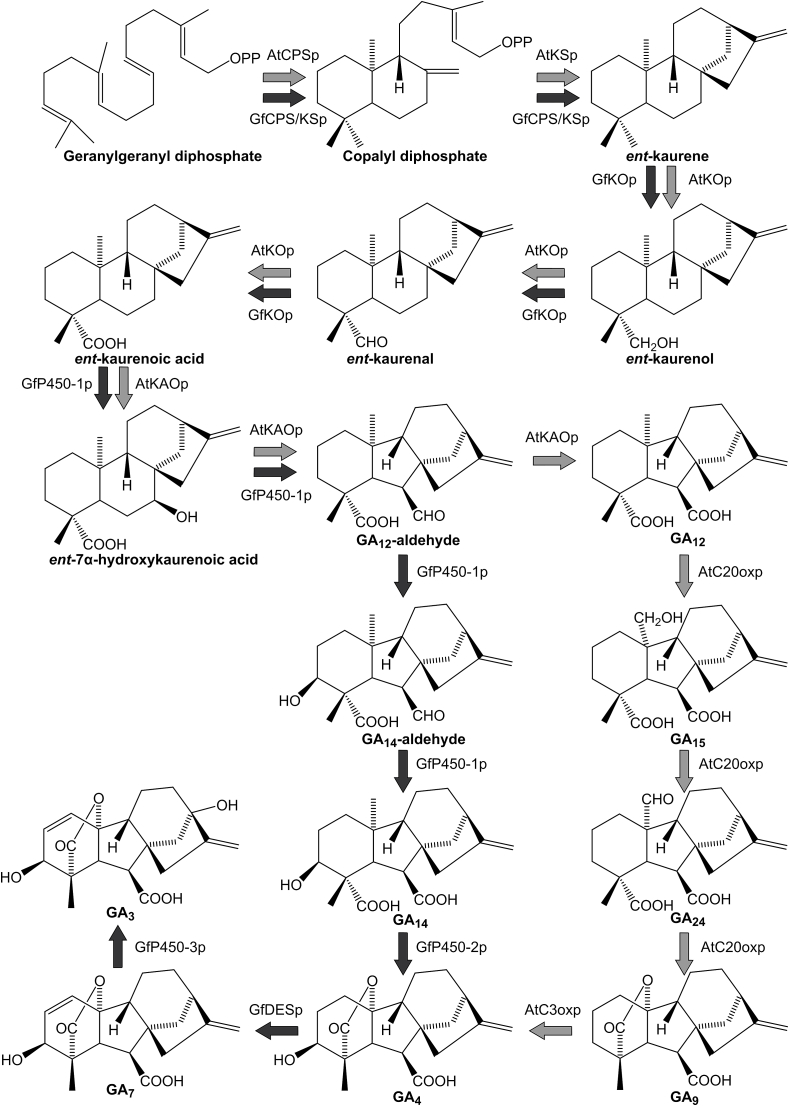
The biosynthetic pathways for bioactive GA_4_, GA_7,_ and GA_3_. Black arrows indicate GA-biosynthetic enzymes from *F. fujikuroi* (Gf), and grey arrows indicate GA-biosynthetic enzymes from *A. thaliana* (At). CPSp, copalyl disphosphate synthase. KSp, *ent*-kaurene synthase. CPS/KSp, bifunctional copalyl diphosphate synthase and *ent*-kaurene synthase. KOp, *ent*-kaurene oxidase. KAOp, *ent*-kaurenoic acid oxidase. C20oxp, GA carbon 20 oxidase. C3oxp, GA carbon 3 oxidase. P450-1p, P450-2p, P450-3p, cytochromes P450. DES, GA desaturase.

In plants, gibberellins are mainly produced through the plastidial methylerythritol pathway (MEP), while in fungi, they are produced through the mevalonate pathway (MVA) (Kasahara et al., 2002; Albermann et al., 2013b). The initial steps of gibberellin biosynthesis from geranylgeranyl diphosphate (GGPP) to GA_12_-aldehyde are similar in fungi and plants. Cyclization of GGPP into *ent-*copalyl diphosphate and then into *ent-*kaurene is catalyzed by the copalyl diphosphate synthase (CPSp) and the *ent*-kaurene synthase (KSp) in plants. In fungi, one bi-functional synthase (CPS/KSp) forms *ent*-kaurene (Fig. 1) (Sun and Kamiya, 1994; Tudzynski et al., 1998, 2001; Yamaguchi et al., 1998). *Ent-*kaurene is hydroxylated to *ent-*kaurenol, deprotonated to *ent-*kaurenal, and then modified to *ent-*kaurenoic acid (KA) by the *ent-*kaurene oxidase (KOp) (Helliwell et al., 1999; Tudzynski et al., 2001). *Ent-*kaurenoic acid oxidase (KAOp) then catalyzes the formation of GA_12_-aldehyde via the intermediate *ent-*7α-hydroxykarenoic acid (Helliwell et al., 2001a). In plants, KAOp also forms GA_12_ and is located in the ER-membrane, while CPSp, KSp, and KOp are located in plastids (Helliwell et al., 2001b). In fungi, KAOp (P450-1p) converts GA_12_-aldehyde into GA_14_-aldehyde and then into GA_14_ (Rojas et al., 2001). In plants, the GA 20-oxidase can convert GA_12_ into GA_9_ via GA_15_ and GA_24_, while GA 3-oxidase converts GA_9_ into the bioactive GA_4_ (Lange et al., 1994; Williams et al., 1998). In fungi, GA_14_ is converted to GA_4_ by a cytochrome P450 monooxygenase (P450-2p) (Tudzynski et al., 2002). Subsequently, GA_4_ is converted to GA_7_ by the GA desaturase (DESp) and then to GA_3_ by another monooxygenase (P450-3p) (Tudzynski et al., 2003).

While several fungi species like *Sphaceloma manihoticola* or *Phaeosphaeria* sp. and bacteria species like *Rhizobium phaseoli* or *Azospirillum lipoferum* can produce gibberellins, the industrially used microbe for gibberellin production is the fungus *Fusarium fujikuroi* (formerly *Gibberella fujikuroi*) (Rademacher and Graebe, 1979; Atzorn et al., 1988; Bottini et al., 1989; Sassa et al., 1994; Shi et al., 2017). Submerged fermentation is most common as solid-state fermentation is complicated to scale-up (Camara et al., 2018). Titers of 3.9 g/L GA_3_ for an eight-day submerged fermentation with fungal mycelium immobilized on calcium-polygalacturonate beads have been reported in the literature (Escamilla S et al., 2000). While GA_3_ from submerged fermentation can be recovered with column-based adsorption or liquid-liquid extraction, it is more challenging to separate GA_4_ from GA_7_ due to their high structural similarity (Rademacher, 2015; Camara et al., 2018). Since GA_4_ degrades faster than GA_3_ and GA_7_, it can be more useful for some applications, yet most GA_4_ products contain at least some GA_7_ (Rademacher, 2015).

While literature describes mutated strains of *F. fujikuroi* that are able to produce predominately GA_7_ at 234 mg/L or solely GA_4_ at 420 mg/L during ten days of fermentation, it is unclear whether these strains can be used industrially (Lale and Gadre, 2010; Albermann et al., 2013a). Although genome data and transformation procedures are available for *F. fujikuroi*, the molecular toolkit for genome modification is still limited compared to more conventional host organisms (Wiemann et al., 2013; Hwang and Ahn, 2016; Bashyal et al., 2017; Shi et al., 2019). Furthermore, commercial efforts to produce pure GA_4_ with *S. manihoticola* have failed (Rademacher, 2015). Moving the production into a heterologous host with more molecular tools available and established procedures for industrial-scale production could enable improved control of relative levels of bioactive GAs, better titers, and reduced production costs.

The oleaginous yeast species *Yarrowia lipolytica* is a promising host for the production of lipids and other compounds derived from acetyl- or acyl-CoAs, due to their high endogenous levels (Darvishi et al., 2018; Marella et al., 2018). Furthermore, *Y. lipolytica* offers other benefits due to GRAS status being granted to several production strains, amenability to genetic engineering, sequenced genomes, ability to express functional P450 enzymes, and the presence of lipid bodies for terpene sequestration (Dujon et al., 2004; Christen and Sauer, 2011; Groenewald et al., 2014; Gao et al., 2017; Holkenbrink et al., 2018). Indeed, *Y. lipolytica* has already been engineered to produce a variety of terpenoids, such as d-limonene, α-farnesene, betulinic acid, lycopene, and astaxanthin (Matthäus et al., 2014; Cao et al., 2016; Yang et al., 2016; Gao et al., 2017; Kildegaard et al., 2017; Sun et al., 2019). *Y. lipolytica* was used industrially to produce omega 3-fatty acids by DuPont (Zhu and Jackson, 2015).

This study demonstrates the production of bioactive gibberellins in *Y. lipolytica* by optimizing *ent*-kaurenoic acid production and establishing heterologous pathways combining enzymes originating from both fungi and plants for the complete biosynthesis of bioactive gibberellins GA_3_, GA_4_, and GA_7_.

## 2 Materials and methods

### 2.1 Strains and media

*Y. lipolytica* strain GB20/ST3683 (*mus*51Δ (*ku70*Δ), *nugm*-Htg2, *ndh*2i, *lys*11^−^, *leu*2^−^, *ura*3^−^, MatB) was a gift from Volker Zickermann (Goethe University Medical School, Institute of Biochemistry II, Germany) (Angerer et al., 2014). *Y. lipolytica* strains were grown on Yeast extract Peptone Dextrose (YPD) or synthetic drop-out media (SC) medium at 30°C. YPD medium contained 20 g/L peptone, 10 g/L yeast extract, 20 g/L glucose. For growth on solid media, YPD and SC medium were supplemented with 20 g/L agar. When necessary, the YPD medium was supplemented with antibiotics hygromycin (50 mg/L) or nourseothricin (250 mg/L). Cultivation of the recombinant strains for isoprenoid production was performed in Yeast extract Peptone medium containing 80 g/L glucose instead of 20 g/L glucose (YP+8%D).

*Escherichia coli* strain DH5α was used for DNA manipulations. Cells were cultivated at 37°C on Lysogeny Broth (LB) medium supplemented with 100 mg/L ampicillin for plasmid selection.

The chemicals were obtained, if not indicated otherwise, from Sigma-Aldrich. Nourseothricin was purchased from Jena BioScience GmbH (Germany).

### 2.2 Plasmid construction

The primers, biobricks and plasmids used in this study are listed in Supplementary Tables S4, S5, and S6. The biobricks were amplified with PCR using Phusion U polymerase (Thermo Scientific) and assembled into the EasyClone vectors with USER cloning (Holkenbrink et al., 2018). The USER reactions were transformed into *E. coli* and correct assemblies were verified by sequencing. Genes encoding *Arabidopsis thaliana AtCPS* (GenBank: AAA53632.1), *AtKS* (GenBank: AAC39443.1), *AtKO* (GenBank: AAC39507.1), *AtKAO2* (GenBank: ABJ17103.1), *AtC3ox1* (GenBank: AAO64762.1), *AtC20ox1* (GenBank: AEE36106.1), *AtATR2* (GenBank: AEE85737.1), *Fusarium (Gibberella) fujikuroi GfCPS/KS* (GenBank: BAA84917.1), *GfP450-1* (GenBank: CAA75565.1), *GfP450-2* (GenBank: CAA75566.1), *GfP450-3* (GenBank: CAA75567.1), *GfP450-4* (GfKO; GenBank: CCT69175.1), *GfDES* (GenBank: CAD10289.1), *GfCPR* (GenBank: CAE09055.1), *GfCyb5* (GenBank: KLO84088.1), *GfCybRed* (GenBank: KLO80170.1), and *Synechococcus* sp. *SsGGPPs7* (NCBI Reference Sequence: WP_011429285.1) were codon-optimized for *Y. lipolytica* and synthesized as GeneArt String DNA fragments or Synthetic Genes by Thermo Fisher Scientific (see Supplementary sequences for full DNA sequences). The software ChloroP v1.1 was used to predict chloroplast targeting protein sequences (Emanuelsson et al., 1999). The software InterProScan was used to predict the presence of protein transmembrane regions (Jones et al., 2014; Blum et al., 2021).

A Hygromycin B resistance cassette included in BioBrick BB2080 was cloned into plasmids pCfB6409, pCfB6410, pCfB6411, pCfB6412, pCfB6413, pCfB6414, and pCfB6415 between SacI and HpaI restriction sites in order to build plasmids from pCfB6601 to pCfB6607 (Supplementary Table S6).

Vectors pCfB6607 and pCfB6408 were constructed via point mutation of parental plasmids using primers indicated in Supplementary Table S4 and following indications of QuickChange II Site-Directed Mutagenesis Kit (Agilent Technologies).

### 2.3 Yeast strain construction

All strains used in this study are listed in Supplementary Table S7. The plasmids for targeted integration were *Not*I-linearized and transformed into *Y. lipolytica* cells using an adapted protocol based on the published lithium acetate transformation protocol (Holkenbrink et al., 2018). In detail, the parental strain was spread over a YPD agar and grown for 24 h at 30°C prior to transformation. Cells were scraped from the plate and washed with water. Subsequently, a certain amount of cells (volume corresponding to OD_600_ 9.2 per transformation) was resuspended in 100 μL of a transformation mix containing 43.8% poly-ethylene glycol (PEG 3350), 100 mM dithiothreitol, 0.25 g/L single-stranded DNA and 0.1 M LiAc. DNA was then added (a minimum of 500 ng). After a heat-shock at 39°C for 1 h, cells were pelleted for 5 min at 3000 rpm. The transformation mix was removed, and cell pellets were resuspended in 1 mL YPD and allowed to recover for 2 h at 30°C, 250 rpm agitation. Finally, cells were pelleted for 5 min at 3000 rpm, resuspended in 100 μL sterile water, and plated on selective medium. Plates were incubated at 30°C for 2–5 days until colonies appeared.

All strains were verified by PCR using insert-specific oligos in combination with oligos specific to the regions outside the different recombination sites. The CreA-loxP-mediated selection marker loop-out was performed as described previously (Holkenbrink et al., 2018). The elimination of the selection marker was verified by PCR in addition to phenotypic tests.

### 2.4 Cultivation

For KA-quantification, single colonies were inoculated from fresh plates into 3 mL YPD in 24-well plates with air-penetrable lids (EnzyScreen, NL) and grown for 24 h at 30°C and 300 rpm agitation as pre-cultures. For cultivation, the required volume of the pre-culture was transferred to 50 ml YP+8%D in 250 mL baffled shake flasks for an initial optical density at 600 nm (OD_600_) of 1.0. The shake flask cultures were cultivated for 72 h at 30°C with 300 rpm agitation.

For GA-quantification, single colonies were inoculated from fresh plates into 2.5 or 3 mL YPD in 24-well plates with air-penetrable lids and grown for 16–24 h at 30°C and 300 rpm agitation as pre-cultures. For cultivation, the required volume of the pre-culture was transferred to 2.5 or 3 mL YP+8%D in 24-well plates for an initial optical density at 600 nm (OD_600_) of 0.1. The plates were cultivated for 72 h at 30°C with 300 rpm agitation. By the end of the cultivation, either cell dry weight, OD_600_, or both were measured. OD_600_ was measured using a NanoPhotometer (Implen GmbH, Germany). All cultivations were done in biological triplicates.

For the feeding experiments with GA_9_, GA_4_ or GA_7_, pre-cultures and cultivation were done under the same conditions as for GA-quantification. During the cultivation step, 100 mg/L of either GA_9_, GA_4_ or GA_7_ was added to the media (3 or 2.5 ml of YP+8% glucose). The stock solutions of GA_9_, GA_4,_ and GA_7_ were 5 mg/mL in absolute ethanol, which yielded a final ethanol concentration of 2% in the media.

For growth profiling of selected strains, pre-culture preparations from single colonies were inoculated from fresh plates into 2.5 YPD in 24-well plates with air-penetrable lids and grown for ~16 h at 30°C and 300 rpm agitation. Then the required volume of pre-culture was transferred to 1 mL of YP+8%D in 24-roundwell plates (Enzyscreen, CR1424f) with air-penetrable lids. The plates were cultivated, and their growth continuously measured in a Growth Profiler 960 (Enzyscreen) at 30°C with 225 rpm agitation for 72 h. All growth profiling experiments were conducted in biological triplicates.

### 2.5 Analytical methods

For the KA-quantification, GA_12_-detection, GA-analysis of ST6513 and ST6514, and feeding experiments, the supernatant was sampled directly in the case of cell-free media or extracted by the following protocol. 300 μL of cultivation broth was transferred into a 2 ml microtube (Sarstedt). 500 μL of 0.5–0.75 mm acid-washed glass beads and 1.2 mL of acetonitrile were added to each tube. Thereafter, the cells were disrupted with a Precellys®24 homogenizer (Bertin Corp.) four times at 5500 rpm for 10 s, with samples being kept on ice in between rounds of breaking. The samples were then shaken for 10 min at room temperature. Lastly, the samples were centrifuged, and the supernatant fractions were sampled for analysis.

For quantification of GA_9_, GA_4_, GA_7_, and GA_3_ for all other strains, 1 mL of broth were transferred to a 2 mL microtube. The samples were centrifuged, and the supernatant was extracted for analysis. Samples were diluted in water prior to analysis.

Furthermore, to investigate the presence of GA_9_, GA_4_, GA_7_, and GA_3_ in ST8580 cell pellets, 1 mL of broth was transferred 2 mL microtube. The cells were pelleted by centrifugation, and the supernatant was removed. The cell pellets were washed twice by resuspension in 1 mL of water, centrifugation, and removal of the liquid phase. Thereafter, 500 μl of 0.5–0.75 mm acid-washed glass beads and 1 mL of acetonitrile were added to each tube. Subsequently, the cells were disrupted with a Precellys®24 homogenizer four times at 5500 rpm for 10 s, with samples being kept on ice in between rounds of breaking. The samples were then shaken for 10 min in a DVX-2500 Multi-tube vortexer at room temperature. Lastly, the tubes were centrifuged, and the acetonitrile fractions were sampled for analysis.

The quantitative LC-MS data for the feeding experiments, KA-quantification, GA-quantification for ST6513 and ST6514, and GA_12_ mass confirmation can be seen in the supplementary methods.

The LC-MS/MS analysis of GAs for all other strains was performed on a Vanquish Duo UHPLC binary system (Thermo Fisher Scientific, USA) coupled to IDX-Orbitrap Mass Spectrometer (Thermo Fisher Scientific, USA). The chromatographic separation was achieved using a Waters ACQUITY BEH C18 (10 cm × 2.1 mm, 1.7 μm) equipped with an ACQUITY BEH C18 guard column kept at 40 C and using a flow rate of 0.35 mL/min. The mobile phases consisted of MilliQ© water + O.1% formic acid (A) and acetonitrile + 0.1% formic acid (B). The initial composition was 2%B held for 0.8 min, followed by a linear gradient till 5% in 3.3 min, and afterward, 100%B was reached in 10 min and held for 1 min before going back to initial conditions. Re-equilibration time was 2.7 min. The MS measurement was done in negative-heated electrospray ionization (HESI) mode with a voltage of 2500 V acquiring in full MS/MS spectra (Data dependent Acquisition-driven MS/MS) in the mass range of 70–1000 Da. The authentic GA_3_ (012 2503), GA_4_ (012 2532), GA_7_ (012 2543), and GA_9_ (012 2663) standards were acquired from OlChemIm s.r.o. (Czech Republic) and the detection limits of all GAs were 0.05 mg/L.

## 3 Results

### 3.1 Establishing the biosynthetic pathway to *ent*-kaurenoic acid

**Fig. 2:**
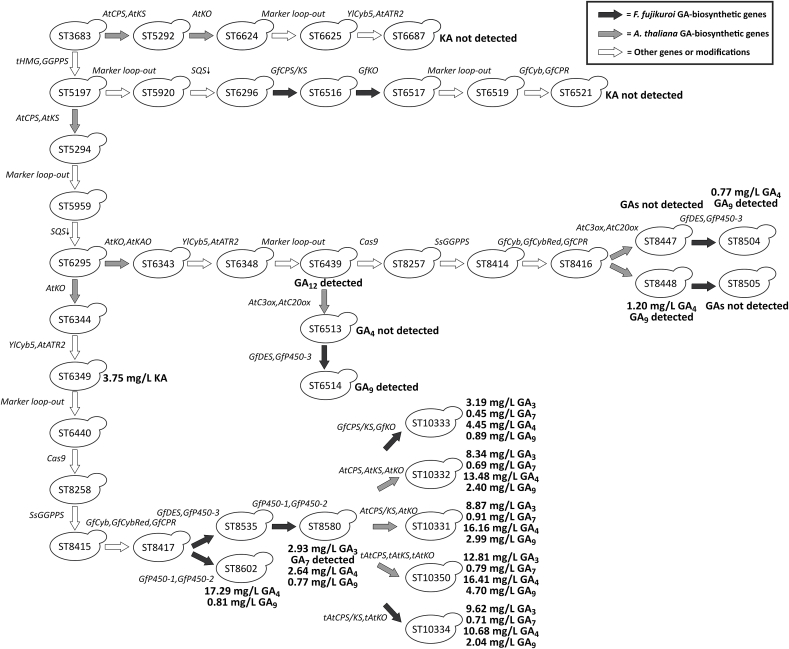
Overview of the strain engineering process of *Y. lipolytica* for GA-production. Italicized genes were expressed or overexpressed. *SQS*↓, downregulation of the squalene synthase gene by promoter truncation. ‘Marker loop-out’, removal of selection markers by Cre/*loxP*-recombination.

We began with evaluating plant and fungal biosynthetic pathways for *ent*-kaurenoic acid production in *Y. lipolytica*. All genes that were not native to *Y. lipolytica* were codon-optimized. To produce the GA-precursor *ent*-kaurenoic acid via the plant pathway, we expressed the genes encoding copalyl diphosphate synthase (*AtCPS*), *ent*-kaurene synthase (*AtKS*), *ent-*kaurene oxidase (*AtKO*), NADPH cytochrome P450 reductase (*AtATR2*) from *A. thaliana,* and the native *Y. lipolytica* cytochrome *b*5 (*YlCyb5*) to generate strain ST6687 (Fig. 2). The genes encoding *AtCPS*, *AtKS*, and *AtKO* were full-length with the native plastidial signal peptides intact. Furthermore, an MVA-pathway optimized strain ST6349 was constructed that expressed the same genes as ST6687, a truncated version of the native 3-hydroxy-3-methyl-glutaryl-coenzyme A reductase (*tHMG*), and the native geranylgeranyl diphosphate synthase (*GGPPS*), while the endogenous squalene synthase gene was downregulated by truncating its promoter down to 50 base pairs (*SQS*↓) (Kildegaard et al., 2017). The HMG-reductase (HMGp) that reduces HMG-CoA to mevalonate was previously reported as one of the flux-controlling enzymes in the MVA-pathway (Cao et al., 2016, 2017; Yang et al., 2016). Furthermore, in *Saccharomyces cerevisiae,* removing the N-terminal transmembrane domain of HMGp resulted in a non-regulated protein retaining its catalytic activity (Donald et al., 1997).

Overexpression of GGPPSp has been shown to increase the production of β-carotene in *Y. lipolytica* and gibberellins in *F. fujikuroi* (Albermann et al., 2013b; Kildegaard et al., 2017). The final modification in the MVA-optimized strains is the truncation of the native squalene promoter, which was previously shown to increase β-carotene production in *Y. lipolytica* (Kildegaard et al., 2017).

To produce *ent*-kaurenoic acid via the fungal pathway, we expressed the genes encoding the bifunctional *F. fujikuroi* copalyl disphosphate synthase/*ent*-kaurene synthase (*GfCPS/KS*), *F. fujikuroi ent*-kaurene oxidase (*GfKO*), *F. fujikuroi* NADPH cytochrome P450 reductase (*GfCPR*) and *F. fujikuroi* cytochrome *b*5 (*GfCyb5*), while *tHMG* and *GGPPS* were overexpressed, and the squalene promoter truncated (*SQS*↓), which generated ST6521.

**Fig. 3:**
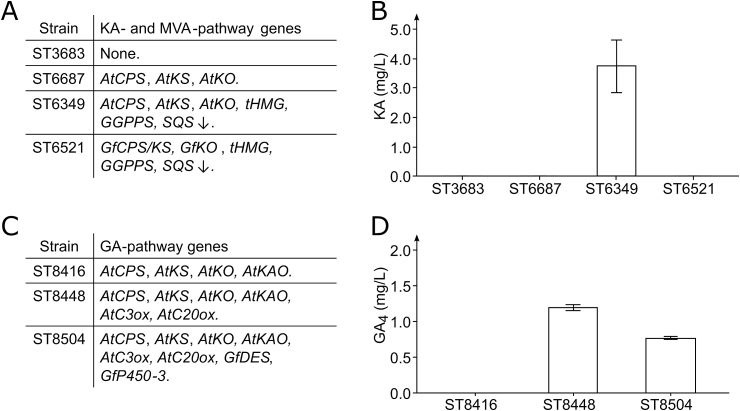
KA-production by engineered *Y. lipolytica* strains. **A.** Overview of KA-producing strains showing which MVA-pathway genes were overexpressed or downregulated and which KA-biosynthetic genes were expressed. **B.** KA-production for ST3683, ST6687, ST6349, and ST6521. **C.** Overview of GA-producing strains showing which GA-biosynthetic genes were expressed. **D.** GA_4_-production for ST8416, ST8448, and ST8504. Three biological replicates were used to calculate titer averages and standard deviations for all strains.

The MVA-optimized strain ST6349 expressing the plant KA-biosynthetic pathway produced 3.75 mg/L KA, while no KA was detected for the non-optimized strain ST6687 (Fig. 3A and B). Surprisingly, the strain ST6521 with optimized MVA-pathway and expressing fungal enzymes did not produce measurable amounts of KA*.*

### 3.2 Production of the early gibberellin intermediates

Since KA production was achieved with the plant pathway enzymes, we sought to reconstruct the pathway towards bioactive GAs with mainly plant-derived enzymes. The strain ST6439 for production of the inert intermediate GA_12_ was constructed based on the *ent*-kaurene producer ST6295 and further expression of *AtKO*, *A. thaliana ent-*kaurenoic acid oxidase (*AtKAO*), *YlCyb5,* and *AtATR2*. After cultivation, a compound identified as GA_12_ could be detected for ST6439 based on targeted ion mass and retention time (Fig. S1). Since no standard was available, the amount of putative GA_12_ could not be quantified.

### 3.3 Introduction of the biosynthetic pathway for bioactive gibberellin production

As the next step, we wanted to extend the pathway from GA_12_ to the biologically active gibberellin GA_4_. For this, we expressed codon-optimized versions of the *A. thaliana* GA C20-oxidase (*AtC20ox*) and GA C3-oxidase (*AtC3ox*) in the GA_12_-producing strain ST6439 to generate ST6513 that should be able to produce GA_4_ from GA_12_ via the intermediates GA_15_, GA_24,_ and GA_9_. However, the cultivation of ST6513 did not result in detectable amounts of GA_4_. However, we still attempted to further extend the pathway from GA_4_ to GA_7_ and to GA_3_ by expressing fungal enzymes GfP450-3p and GfDESp generating ST6514.

While GA_9_ was detected for ST6514, neither GA_4_, GA_7,_ nor GA_3_ were detected (Table S2). This indicated that AtC20oxp was active in ST6514 and catalyzed the formation of GA_9_ from GA_12_. To discern whether the three biosynthetic enzymes, AtC3oxp, GfDESp, and GfP450-3p, were active, we conducted several precursor feeding experiments. When strain ST6514 was cultivated in a medium supplemented with GA_9_, it produced detectable amounts of GA_4_, which was not the case for the control strain ST3683 (Table S2). This confirmed that AtC3oxp was active in ST6514 and that the formation of GA_4_ was limited by GA_9_-supply. When the ST6514 was supplemented with GA_4_, the presence of GA_7_ was detected; However, GA_7_ was also detected when ST3683 and cell-free media were supplemented with GA_4_ (Table S2 and S3). This suggests that GA_4_ is either spontaneously converted to GA_7_ or that the commercial GA_4_ product contains small amounts of GA_7_. The manufactures (OlChemIm s.r.o.) note that the GA_4_ standard we used is ≥90% pure, which indicates the presence of impurities (OlchemIm s.r.o.|Products, 2021). Furthermore, other providers note that their GA_4_ standards contain at least some GA_7_ (Sigma-Aldrich|Gibberellin A4, 2021). Indeed, GA_4_ and GA_7_ are difficult to separate during purification, which may explain such impurities (Rademacher, 2015; Camara et al., 2018). Supplementing the cultivation medium of ST6514 with GA_7_ did not lead to a detectable amount of GA_3_ (Table S2). In summary, the results indicate that AtC20oxP and AtC3oxp were active_,_ while GfP450-3p was likely inactive when expressed in ST6514.

### 3.4 Production of GA_4_ by further engineering

We decided to perform additional metabolic engineering of the yeast strain ST6349 to increase the flux towards the GA-biosynthesis. To simplify the genome editing, we integrated a codon-optimized *cas9* gene from *Streptococcus pyogenes* into GA_12_-producing strain ST6439, resulting in strain ST8257 (Fig. 2). This enabled marker-free integration of DNA constructs (Holkenbrink et al., 2018). Firstly, to further boost the production of GGPP, a codon-optimized version of the *Synechococcus sp*. GGPP synthase (*SsGGPPS7*) was expressed in ST8257, giving strain ST8414. Expression of *SsGGPPS7* was previously shown to substantially increase carotenoid production in *Y. lipolytica* (Tramontin et al., 2019). Secondly, three cytochromes P450 partners: cytochrome *b*5, cytochrome *b*5 reductase, and cytochrome P450 reductase (*GfCyb5*, *GfCyb5Red*, *GfCPR*) from *F. fujikuroi* were expressed to promote the electron transfer from NADPH to *F. fujikuroi* cytochromes P450 for the last steps towards GA_3_, resulting in strain ST8416. Finally, *AtC20ox* and *AtC3ox* were expressed under stronger promoters to overcome the potential rate-limitation of these steps. The genes *AtC20ox* and *AtC3ox* were expressed under the control of the promoters *PrExp* and *PrTefintron*, respectively, to generate strain ST8447 and under promoters *PrTefintron* and *PrGpd*, respectively, to generate ST8448. Both strains should be capable of producing GA_4_, albeit potentially at different levels due to the different promoter pairs used. Furthermore, the *PrTefintron* promoter is currently the strongest known constitutive promoter for *Y. lipolytica* (Tai and Stephanopoulos, 2013; Holkenbrink et al., 2018).

Cultivation of ST8448 resulted in the production of 1.20 mg/L GA_4_ and trace amounts of GA_9_ (Fig. 3C and D, and S2A), whereas no GA_4_ or GA_9_ could be detected for ST8447. The difference between the strains may be explained by the low expression level of *AtC20ox* from the *PrExp* promoter in ST8447 (Holkenbrink et al., 2018). After establishing *de novo* production of GA_4_ in yeast, we proceeded to complete the biosynthetic pathway towards GA_7_ and GA_3_. For this, *GfDES* and *GfP450-3* were expressed in both strains ST8447 and ST8448, resulting in strains ST8504 and ST8505, respectively. Cultivation of ST8504 resulted in the production of 0.77 mg/L GA_4_ and trace amounts of GA_9,_ while neither GA_7_ nor GA_3_ were detected (Fig. 3C and D, and S2A). None of these four compounds were detected in strain ST8505. This contradicts the previous results, where only the parental strain of ST8505 produced GA_4_.

Feeding of ST8504 with GA_7_ did not result in detectable amounts of GA_3,_ which suggested that GfP450-3p was inactive in this strain (Table S3). This strain also grew very poorly when supplemented with GA_7_ and 2% ethanol (3 g dry cell weight/L) compared to without (15 g dry cell weight/L). Furthermore, GA_4_ was also detected when both ST3683 and cell-free media were supplemented with GA_7_, possibly due to product impurities (Table S3).

### 3.5 Production of GA_3_, GA_4_, and GA_7_ by the introduction of fungal cytochromes P450

Since no GA_3_ could be detected by expressing the plant genes *AtC20ox*, *AtC3ox,* and fungal genes *GfDES* and *GfP450-3*, we sought to reconstruct the biosynthetic pathway KA to GA_3_, GA_7_, and GA_4_ with fungal enzymes.

We built on top of the KA-producing strain ST6440, into which, through several rounds of transformation, we integrated the genes encoding for *Cas9*, *SsGGPPS*, and P450 auxiliary proteins *GfCyb5*, *GfCybRed*, *GfCPR*, generating ST8417. Since *Cas9* expression may be toxic to yeast, we investigated the growth profiles of the *Cas9* expressing strain ST8258 and its parental strain ST6440, however, no reductions in the growth of ST8258 compared to ST6440 were found (Fig. S3A and S3B).

**Fig. 4:**
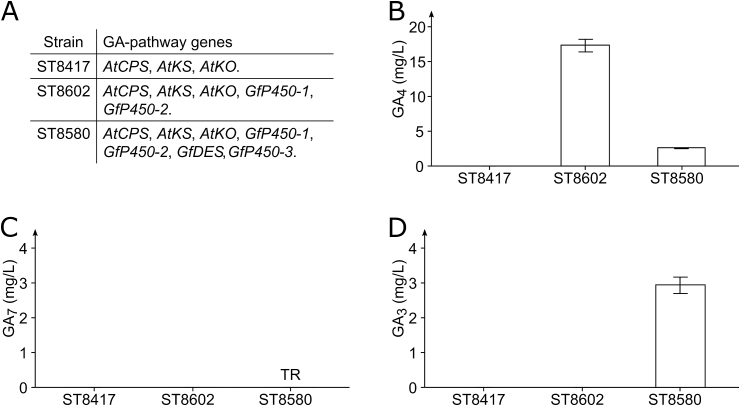
GA-production by engineered *Y. lipolytica* strains. **A.** Overview of GA-producing strains showing which GA-biosynthetic genes were expressed. **B.** GA_4_-production for ST8417, ST8602, and ST8580. **C.** GA_7_-production for ST8417, ST8602, and ST8580. **D.** GA_3_-production for ST8417, ST8602, and ST8580. TR, trace amounts. Three biological replicates were used to calculate titer averages and standard deviations for all strains.

Furthermore, we expressed two fungal genes *GfP450-1* and *GfP450-2,* encoding cytochromes P450 that should convert KA into GA_4_. The resulting strain ST8602 produced 17.29 mg/L GA_4_ and 0.81 mg/L GA_9_ (Fig. 4A and B, and S2B). The presence of GA_9_ is likely due to the oxygenation of GA_12_-aldehyde by GfP450-1p resulting in GA_12_, which is then processed into GA_9_ by the action of GfP450-2p (Tudzynski et al., 2002). The titer of GA_4_ in strain ST8602 expressing *GfP450-1* and *GfP450-2* was 14.4-fold greater than what was achieved for the analogous strain ST8448 expressing *AtC20ox* and *AtC3ox*. As the next step, the strain ST8580 was built that combined all the modifications as in ST8602 and additionally expressed *GfP450-3* and *GfDES*. Cultivation of ST8580 resulted in the production of 2.93 mg/L GA_3_, trace amounts of GA_7_, 2.64 mg/L GA_4_, and 0.77 mg/L GA_9_ (Fig. 4A–D, and S2B). The identities of the compounds were confirmed by retention time and high-resolution mass-spectrometry compared to authentic standards (Fig. S4). Furthermore, analysis of the cell fraction of ST8580 did not show detectable amounts of GA_7_ and only trace amounts of GA_3_, GA_4_, and GA_9_, indicating that all GAs were secreted. Lastly, we investigated the growth profiles of the GA-producing strains ST8580 and ST8602 compared to their parental strain ST8417. There were no significant differences in growth (Fig. S3A and S3C).

### 3.6 Improved GA-production by increasing early GA-pathway flux

**Fig. 5:**
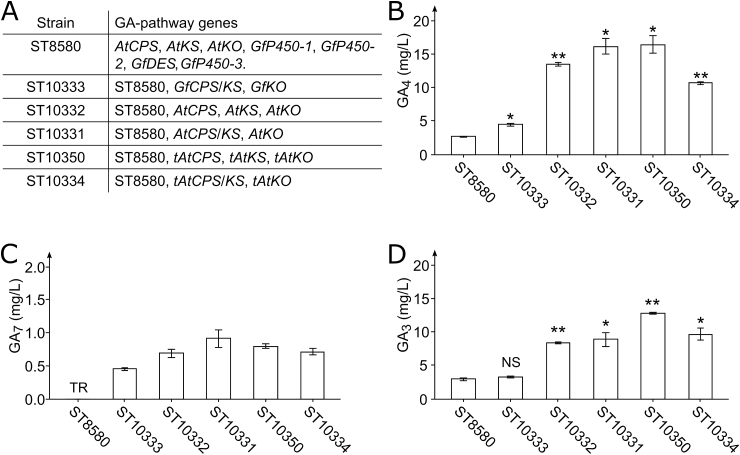
*Y. lipolytica* strains engineered for improved GA-production. **A.** Overview of GA-producing strains showing which GA-biosynthetic genes were expressed. **B.** GA_4_-production for ST8580, ST10333, ST10332, ST10331, ST10350, and ST10334. **C.** GA_7_-production for ST8580, ST10333, ST10332, ST10331, ST10350, and ST10334. **D.** GA_3_-production for ST8580, ST10333, ST10332, ST10331, ST10350, and ST10334. TR, trace amounts. Three biological replicates were used to calculate titer averages and standard deviations for all strains. Statistical significance (two-tailed student’s t-test) compared to ST8580 is represented by asterisks (*, p < 0.05. **, p < 0.001. NS, not significant).

To increase GA-production, we expressed either *GfCPS/KS* and *GfKS* or *AtCPS*, *AtKS*, and *AtKO* in ST8580, generating strains ST10333 and ST10332, respectively. A fused enzyme consisting of *AtCPS* C-terminally linked to the N-terminal of *AtKS* (*AtCPS*/*KS*) was expressed together with *AtKO* in ST8580, generating ST10331. Furthermore, truncated variants of the *A. thaliana* KA-biosynthetic enzymes with N-terminal plastidial targeting sequences removed (*tAtCPS*, *tAtKS*, *tAtKO)* were expressed under individual promoters or with *tAtCPS* fused to *tAtKS* (*tAtCPS/KS*) alongside the individual expression of *tAtKO* in ST8580 generating ST10350 and ST10334, respectively. The ChloroP software and earlier reports were used to predict the plastidial targeting sequences of AtCPSp and AtKOp based on which the 2–60 and 2–28 amino acids were removed to make tAtCPSp and tAtKOp, respectively (Emanuelsson et al., 1999; Helliwell et al., 2001b). Of note, a transmembrane region at amino acid positions 6–24 was predicted for AtKOp by the InterProScan software, whereas no transmembrane regions were predicted for pAtCPS and pAtKS (Jones et al., 2014; Blum et al., 2021). No plastidial targeting sequence for AtKSp was predicted by ChloroP; however, earlier reports confirm the presence of a plastidial targeting sequence in AtKSp and suggest that the 44 first amino acids of AtKSp are involved in the translocation (Yamaguchi et al., 1998; Helliwell et al., 2001b). ST10350 exhibited the most enhanced GA-production with 12.81 mg/L GA_3_, 16.41 mg/L GA_4_, and 4.70 mg/L GA_9_, a 4.4-, 6.2- and 6.1- fold increase over ST8580, respectively (Fig. 5). Interestingly, all strains constructed from ST8580 demonstrated improved growth rates compared to ST8580 on YP+8%D media, likely due to the restoration of uracil prototrophy (Fig. S3A and S3D).

## 4 Discussion

The gibberellins GA_4_, GA_7_, and GA_3_ are plant hormones with several applications in agriculture. The current production methods for these GAs are based on fermentation of the fungus *F. fujikuroi* (Shi et al., 2017). We have demonstrated the production of GA_3_, GA_4_, and GA_7_ in recombinant yeast *Y. lipolytica* by expression of biosynthetic pathway enzymes from *F. fujikuroi* and *A. thaliana*.

The production of KA was established by improving the flux towards GGPP and expression of *AtCPS*, *AtKS*, and *AtKO*. Interestingly, AtKOp has previously been functionally characterized by expression in *S. cerevisiae*, demonstrating its functionality in another yeast species (Helliwell et al., 1999). Yet, the presence of cofactors, substrate, and pH-levels may differ in *Y. lipolytica* compared to the native host environment, which may affect the activity of heterologous enzymes. Indeed, *in vitro* kinetic assays of N-terminally truncated AtCPS demonstrate optimal catalytic rates at 0.1 mM of the cofactor Mg^2+^ with decreasing enzymatic activity at both greater and lesser Mg^2+^-concentrations. A similar inhibitory effect is exerted by the substrate GGPP, while the optimal pH for truncated AtCPS was 7.5–8 (Prisic and Peters, 2007). Differences in such factors could also explain why the expression of *GfCPS/KS* and *GfKO* did not result in KA-production. Furthermore, there exists different algorithms for codon-optimization and it has been shown that codon-optimization can sometimes result in decreased expression or impaired folding of heterologous proteins (Fath et al., 2011; Komar A. A., 2019; Saito et al., 2019; Fu et al., 2020).

It would be interesting for future research to investigate the activity of heterologous KA-biosynthetic genes from various sources and with DNA sequences based on different codon-optimization algorithms in *Y. lipolytica*. Notably, the first two enzymes of KA-biosynthesis, AtCPSp, and AtKSp, are located in plastids in plants, while AtKOp is located on the outer plastidial membrane, and the rest of the GA-biosynthetic enzymes are located in the ER and cytosol (Helliwell et al., 2001b; Hedden and Sponsel, 2015). In the case of AtCPSp, the protein is processed into the mature enzyme during transport into the chloroplast, likely by removal of the N-terminal plastidial targeting peptide, which may yield a better performing enzyme (Sun and Kamiya, 1994; Helliwell et al., 2001b). Indeed, the highest GA_3_ production was achieved by expression of *tAtCPS*, *tAtKS*, and *tAtKO*, which suggests that N-terminal truncation may be a viable strategy for increasing enzyme activity.

Furthermore, the removal of plastidial targeting peptides from heterologous diterpene synthases has also been successfully used to construct yeast cell factories for the production of 13*R*-manoyl oxide (Zhang et al., 2019). In fact, targeting diterpene synthases to the plant cytosol by removal of targeting peptides alongside MVA-pathway upregulation resulted in increased production of both taxadiene, 13*R*-manoyl oxide, and forskolin in *Nicotiana benthamiana* compared to the expression of nontruncated diterpene synthases and MEP-pathway upregulation (De La Peña and Sattely, 2020). Interestingly, a transmembrane domain within the truncated region was predicted for tAtKOp, which likely renders the protein cytosolic upon removal.

Additionally, expression of fused *AtCPS*/*KS* or *tAtCPS/KS* together with *AtKO* or *tAtKO*, respectively, also led to improved GA-production. The fusion of the CPSp and KSp from *Salvia miltiorrhiza* led to enhanced production of *ent*-kaurene in *S. cerevisiae* (Su et al., 2016). Interestingly, certain mosses and liverworts have naturally occurring bifunctional CPS/KSp enzymes similar to *F. fujikuroi* (Hayashi et al., 2006; Kawaide et al., 2011). Therefore, enzymatic fusion could be further explored to improve GA-production in *Y. lipolytica*.

The further expression of AtKAOp resulted in the production of a compound putatively identified as GA_12_. This enzyme has previously been characterized and co-expressed with either *AtATR1* or *AtATR2* in *S. cerevisiae*, where co-expression with *AtATR2* led to increased relative amounts of GA_12_ (Helliwell et al., 2001a). This indicates that optimal redox partner pairing with cytochromes P450 like AtKAOp can lead to improved activity in heterologous systems. Although GA_12_ is considered to be biologically inert, it is implicated in long-range GA signaling in *A. thaliana*, since GA_12_ may be transported via plant vascular tissue, converted to bioactive GAs, and thereby activate GA signaling cascades in receiving tissues, resulting in decreased accumulation of growth inhibitory DELLA proteins (Regnault et al., 2015). Therefore, agricultural GA_12_-application could potentially be useful to enhance plant growth. Furthermore, GA_12_ can be converted by the “Gain of Function in ABA-modulated Seed Germination 2”-enzyme (AtGas2p) to GA_12_ 16, 17-dihydro-16α-ol (DHGA_12_), which can enhance seed germination and seedling development in *A. thaliana* (Liu et al., 2019). Cell factories for the production of GA_12_ and bioactive GA_12_-derivatives may therefore become useful in the future.

While we attempted to complete the biosynthetic pathway towards GA_12_ to GA_3_ by expression of *AtC20ox*, *AtC3ox*, *GfDES*, and *GfP450*-3 to generate ST6514, neither GA_4_, GA_7_, nor GA_3_ were produced by this strain. The feeding studies indicated that GfP450-3p was not active in ST6514. However, GA_3_ was produced when GfP450-3p was expressed in ST8580. Interestingly, ST6514 expressed only *AtATR2* and *YlCyb5*, while ST8580 expressed the fungal redox partners *GfCyb5*, *GfCybRed*, and *GfCPR* in addition to the prior. Therefore, the presence of specific redox partners may be necessary to support the activity of *F. fujikuroi* cytochromes P450. Indeed, previous research has demonstrated that the activity of heterologous cytochromes P450 in yeast depends heavily on their co-expressed redox partners. The cytochrome P450 FoCYP53A19p from *Fusarium oxysporum* could convert 3-hydroxybenzoic acid to 3,4-dihydroxybenzoic acid when paired with an *F. oxysporum* CPR but not when paired with an *S. cerevisiae* or *Candida albicans* CPR (Durairaj et al., 2015). Furthermore, the activity of heterologous cytochromes P450 could be affected by codon-optimization and promoter choice, since the available constitutive promoters for *Y. lipolytica* vary greatly in strength (Holkenbrink et al., 2018).

The GA_3_-titer of 12.81 mg/L reported herein is still very low compared to the highest reported titer of 3.9 g/L for the natural producer *F. fujikuroi* (Escamilla S et al., 2000). However, we demonstrate that GA-production of our strains can be improved considerably by engineering the KA-biosynthetic pathway, and many existing strategies for terpenoid overproduction could be utilized for further improvements.

Indeed, *Y. lipolytica* is a relatively new host for recombinant terpene production, but it has already outperformed *S. cerevisiae* for the production of β-carotene (Larroude et al., 2018; Moser and Pichler, 2019). This is likely due to the higher acetyl-CoA flux and the lipophilic environment in *Y. lipolytica*, which may serve to sequester and store lipophilic terpenes (Christen and Sauer, 2011). Therefore, *Y. lipolytica* holds great potential as a microbial cell factory for terpenoid production.

## 5 Conclusions

Production of bioactive gibberellins GA_3_, GA_4,_ and GA_7_ in *Y. lipolytica* was achieved by expressing a GA-biosynthetic pathway with both plant and fungal enzymes. The complete biosynthesis of GA_3_, GA_4,_ and GA_7_ was done by heterologous expression of *A. thaliana* enzymes for KA-production and *F. fujikuroi* enzymes for the subsequent biosynthetic steps. We demonstrate that the GA-production could be enhanced by further engineering of the yeast chassis. This yeast-based platform holds the potential to improve the future production of bioactive GAs.

## Author statements

Kanchana R. Kildegaard: Conceptualization, methodology, investigation, formal analysis, supervision, writing – original draft, writing – review & editing, funding acquisition. Jonathan A. Arnesen: methodology, investigation, validation, formal analysis, visualization, writing – original draft, writing – review & editing. Belén Adiego-Pérez: Investigation, writing – review & editing. Daniela Rago: methodology, investigation, data curation, visualization. Mette Kristensen: methodology, investigation, data curation, writing – review & editing. Andreas K. Klitgaard: methodology, investigation, data curation, visualization, writing – review & editing. Esben H. Hansen: resources, methodology, writing – review & editing. Jørgen Hansen: resources, writing – review & editing. Irina Borodina: Conceptualization, methodology, supervision, writing – original draft, writing – review & editing, supervision, funding acquisition.

## Declaration of competing interest

No conflicts of interest.

## References

[bib1] Albermann S., Elter T., Teubner A., Krischke W., Hirth T., Tudzynski B. (2013). Characterization of novel mutants with an altered gibberellin spectrum in comparison to different wild-type strains of *Fusarium fujikuroi*. Appl. Microbiol. Biotechnol..

[bib2] Albermann S., Linnemannstöns P., Tudzynski B. (2013). Strategies for strain improvement in *Fusarium fujikuroi*: overexpression and localization of key enzymes of the isoprenoid pathway and their impact on gibberellin biosynthesis. Appl. Microbiol. Biotechnol..

[bib3] Angerer H., Radermacher M., Mankowska M., Steger M., Zwicker K., Heide H. (2014). The LYR protein subunit NB4M/NDUFA6 of mitochondrial complex I anchors an acyl carrier protein and is essential for catalytic activity. Proc. Natl. Acad. Sci..

[bib4] Atzorn R., Crozier A., Wheeler C.T., Sandberg G. (1988). Production of gibberellins and indole-3-acetic acid by *Rhizobium phaseoli* in relation to nodulation of *Phaseolus vulgaris* roots. Planta.

[bib5] Bashyal B.M., Rawat K., Sharma S., Kulshreshtha D., Gopala Krishnan S., Singh A.K. (2017). Whole genome sequencing of *Fusarium fujikuroi* provides insight into the role of secretory proteins and cell wall degrading enzymes in causing bakanae disease of rice. Front. Plant Sci..

[bib6] Blum M., Chang H.-Y., Chuguransky S., Grego T., Kandasaamy S., Mitchell A. (2021). The InterPro protein families and domains database: 20 years on. Nucleic Acids Res..

[bib7] Bottini R., Fulchieri M., Pearce D., Pharis R.P. (1989). Identification of gibberellins A_1_, A_3_, and iso-A_3_ in cultures of *Azospirillum lipoferum*. Plant Physiol..

[bib8] Camara M.C., Vandenberghe L.P.S., Rodrigues C., de Oliveira J., Faulds C., Bertrand E. (2018). Current advances in gibberellic acid (GA_3_) production, patented technologies and potential applications. Planta.

[bib9] Cao X., Lv Y.B., Chen J., Imanaka T., Wei L.J., Hua Q. (2016). Metabolic engineering of oleaginous yeast *Yarrowia lipolytica* for limonene overproduction. Biotechnol. Biofuels.

[bib10] Cao X., Wei L.J., Lin J.Y., Hua Q. (2017). Enhancing linalool production by engineering oleaginous yeast *Yarrowia lipolytica*. Bioresour. Technol..

[bib11] Casanova L., Casanova R., Moret A., Agustí M. (2009). The application of gibberellic acid increases berry size of “Emperatriz” seedless grape. Spanish J. Agric. Res..

[bib12] Christen S., Sauer U. (2011). Intracellular characterization of aerobic glucose metabolism in seven yeast species by ^13^C flux analysis and metabolomics. FEMS Yeast Res..

[bib13] Darvishi F., Ariana M., Marella E.R., Borodina I. (2018). Advances in synthetic biology of oleaginous yeast *Yarrowia lipolytica* for producing non-native chemicals. Appl. Microbiol. Biotechnol..

[bib14] De La Peña R., Sattely E.S. (2020). Rerouting plant terpene biosynthesis enables momilactone pathway elucidation. Nat. Chem. Biol..

[bib15] Donald K.A.G., Hampton R.Y., Fritz I.B. (1997). Effects of overproduction of the catalytic domain of 3-hydroxy-3-methylglutaryl coenzyme A reductase on squalene synthesis in *Saccharomyces* cerevisiae. Appl. Environ. Microbiol..

[bib16] Dujon B., Sherman D., Fischer G., Durrens P., Casaregola S., Lafontaine I. (2004). Genome evolution in yeasts. Nature.

[bib17] Durairaj P., Jung E., Park H.H., Kim B.G., Yun H. (2015). Comparative functional characterization of a novel benzoate hydroxylase cytochrome P450 of *Fusarium oxysporum*. Enzym. Microb. Technol..

[bib18] Emanuelsson O., Nielsen H., Heijne G. Von (1999). ChloroP, a neural network-based method for predicting chloroplast transit peptides and their cleavage sites. Protein Sci..

[bib19] Escamilla S E.M., Dendooven L., Magaña I.P., Parra S R., De la Torre M. (2000). Optimization of gibberellic acid production by immobilized *Gibberella fujikuroi* mycelium in fluidized bioreactors. J. Biotechnol..

[bib20] Fath S., Bauer A.P., Liss M., Spriestersbach A., Maertens B., Hahn P. (2011). Multiparameter RNA and codon optimization: a standardized tool to assess and enhance autologous mammalian gene expression. PloS One.

[bib21] Fu H., Liang Y., Zhong X., Pan Z.L., Huang L., Zhang H.L. (2020). Codon optimization with deep learning to enhance protein expression. Sci. Rep..

[bib22] Gao S., Tong Y., Zhu L., Ge M., Zhang Y., Chen D. (2017). Iterative integration of multiple-copy pathway genes in *Yarrowia lipolytica* for heterologous β-carotene production. Metab. Eng..

[bib23] Gibberellins Market (2016). Industry analysis report. https://www.grandviewresearch.com/industry-analysis/gibberellins-market.

[bib24] Groenewald M., Boekhout T., Neuvéglise C., Gaillardin C., Van Dijck P.W.M., Wyss M. (2014). *Yarrowia lipolytica*: safety assessment of an oleaginous yeast with a great industrial potential. Crit. Rev. Microbiol..

[bib25] Hamayun M., Khan S.A., Khan A.L., Shin J.-H., Ahmad B., Shin D.-H. (2010). Exogenous gibberellic acid reprograms soybean to higher growth and salt stress tolerance. J. Agric. Food Chem..

[bib26] Harrington J.F., Rappaport L., Hood K.J. (1957). Influence of gibberellins on stem elongation and flowering of endive. Science.

[bib27] Hayashi K., Kawaide H., Notomi M., Sakigi Y., Matsuo A., Nozaki H. (2006). Identification and functional analysis of bifunctional *ent*-kaurene synthase from the moss *Physcomitrella patens*. FEBS Lett..

[bib28] Hedden P., Sponsel V. (2015). A century of gibberellin research. J. Plant Growth Regul..

[bib29] Helliwell C.A., Chandler P.M., Poole A., Dennis E.S., Peacock W.J. (2001). The CYP88A cytochrome P450, *ent*-kaurenoic acid oxidase, catalyzes three steps of the gibberellin biosynthesis pathway. Proc. Natl. Acad. Sci..

[bib30] Helliwell C.A., Poole A., James Peacock W., Dennis E.S. (1999). Arabidopsis *ent*-kaurene oxidase catalyzes three steps of gibberellin biosynthesis. Plant Physiol..

[bib31] Helliwell C.A., Sullivan J.A., Mould R.M., Gray J.C., James Peacock W., Dennis E.S. (2001). A plastid envelope location of *Arabidopsis ent*-kaurene oxidase links the plastid and endoplasmic reticulum steps of the gibberellin biosynthesis pathway. Plant J..

[bib32] Holkenbrink C., Dam M.I., Kildegaard K.R., Beder J., Dahlin J., Doménech Belda D. (2018). EasyCloneYALI: CRISPR/Cas9-Based synthetic toolbox for engineering of the yeast *Yarrowia lipolytica*. Biotechnol. J..

[bib33] Hwang I.S., Ahn I.-P. (2016). Multi-homologous recombination-based gene manipulation in the rice pathogen *Fusarium fujikuroi*. Plant Pathol. J..

[bib34] Ito A., Sakamoto D., Itai A., Nishijima T., Oyama-Okubo N., Nakamura Y. (2016). Effects of GA_3+4_ and GA_4+7_ application either alone or combined with prohexadione-Ca on fruit development of Japanese pear ‘kosui’. Horticulture J..

[bib35] Jones P., Binns D., Chang H.-Y., Fraser M., Li W., McAnulla C. (2014). InterProScan 5: genome-scale protein function classification. Bioinformatics.

[bib36] Kahn A., Goss J.A., Smith D.E. (1957). Effect of gibberellin on germination of lettuce seed. Science.

[bib37] Kasahara H., Hanada A., Kuzuyama T., Takagi M., Kamiya Y., Yamaguchi S. (2002). Contribution of the mevalonate and methylerythritol phosphate pathways to the biosynthesis of gibberellins in *Arabidopsis*. J. Biol. Chem..

[bib38] Kato Y. (1955). Responses of plant cells to gibberellin. Bot. Gaz..

[bib39] Kawaide H., Hayashi K.I., Kawanabe R., Sakigi Y., Matsuo A., Natsume M. (2011). Identification of the single amino acid involved in quenching the *ent*-kauranyl cation by a water molecule in *ent*-kaurene synthase of *Physcomitrella patens*. FEBS J..

[bib40] Kildegaard K.R., Adiego-Pérez B., Doménech Belda D., Khangura J.K., Holkenbrink C., Borodina I. (2017). Engineering of *Yarrowia lipolytica* for production of astaxanthin. Synth. Syst. Biotechnol..

[bib41] Komar A.A. (2019). Synonymous codon usage—a guide for Co-translational protein folding in the cell. Mol. Biol..

[bib42] Lale G., Gadre R. (2010). Enhanced production of gibberellin A_4_ (GA_4_) by a mutant of *Gibberella fujikuroi* in wheat gluten medium. J. Ind. Microbiol. Biotechnol..

[bib43] Lang A. (1956). Induction of flower formation in biennial hyoscyamus by treatment with gibberellin. Naturwissenschaften.

[bib44] Lange T., Hedden P., Graebe J.E. (1994). Expression cloning of a gibberellin 20-oxidase, a multifunctional enzyme involved in gibberellin biosynthesis. Proc. Natl. Acad. Sci..

[bib45] Larroude M., Celinska E., Back A., Thomas S., Nicaud J.M., Ledesma-Amaro R. (2018). A synthetic biology approach to transform *Yarrowia lipolytica* into a competitive biotechnological producer of β-carotene. Biotechnol. Bioeng..

[bib46] Liu H., Guo S., Lu M., Zhang Y., Li J., Wang W. (2019). Biosynthesis of DHGA_12_ and its roles in *Arabidopsis* seedling establishment. Nat. Commun..

[bib47] MacLeod A.M., Millar A.S. (1962). Effects of gibberellic acid on barley endosperm. J. Inst. Brew..

[bib48] MacMillan J. (2001). Occurrence of gibberellins in vascular plants, fungi, and bacteria. J. Plant Growth Regul..

[bib49] Marella E.R., Holkenbrink C., Siewers V., Borodina I. (2018). Engineering microbial fatty acid metabolism for biofuels and biochemicals. Curr. Opin. Biotechnol..

[bib50] Matthäus F., Ketelhot M., Gatter M., Barth G. (2014). Production of lycopene in the non-carotenoid-producing yeast *Yarrowia lipolytica*. Appl. Environ. Microbiol..

[bib51] Moser S., Pichler H. (2019). Identifying and engineering the ideal microbial terpenoid production host. Appl. Microbiol. Biotechnol..

[bib52] OlchemIm s.r.o (2021). Products. https://www.olchemim.cz/Products.aspx?idc=1&idp=4.

[bib53] Prisic S., Peters R.J. (2007). Synergistic substrate inhibition of *ent*-copalyl diphosphate synthase: a potential feed-forward inhibition mechanism limiting gibberellin metabolism. Plant Physiol..

[bib54] Rademacher W. (2015). Plant growth regulators: backgrounds and uses in plant production. J. Plant Growth Regul..

[bib55] Rademacher W., Graebe J.E. (1979). Gibberellin A_4_ produced by *Sphaceloma manihoticola*, the cause of the superelongation disease of cassava (*Manihotesculenta*). Biochem. Biophys. Res. Commun..

[bib56] Regnault T., Davière J.-M., Wild M., Sakvarelidze-Achard L., Heintz D., Carrera Bergua E. (2015). The gibberellin precursor GA_12_ acts as a long-distance growth signal in *Arabidopsis*. Nature Plants.

[bib57] Rojas M.C., Hedden P., Gaskin P., Tudzynski B. (2001). The P450-1 gene of *Gibberella fujikuroi* encodes a multifunctional enzyme in gibberellin biosynthesis. Proc. Natl. Acad. Sci..

[bib58] Saito Y., Kitagawa W., Kumagai T., Tajima N., Nishimiya Y., Tamano K. (2019). Developing a codon optimization method for improved expression of recombinant proteins in actinobacteria. Sci. Rep..

[bib59] Sassa T., Kawaide H., Takarada T. (1994). Identification of gibberellins A_4_ , A_9_ , and A_24_ from *Phaeosphaeria* sp. L487 cultured in a chemically defined medium. Biosci. Biotechnol. Biochem..

[bib60] Shi T.Q., Gao J., Wang W.J., Wang K.F., Xu G.Q., Huang H. (2019). CRISPR/Cas9-Based genome editing in the filamentous fungus *Fusarium fujikuroi* and its application in strain engineering for gibberellic acid production. ACS Synth. Biol..

[bib61] Shi T.Q., Peng H., Zeng S.Y., Ji R.Y., Shi K., Huang H. (2017). Microbial production of plant hormones: opportunities and challenges. Bioengineered.

[bib62] Shomeili M., Nabipour M., Meskarbashee M., Memari H.R. (2011). Effects of gibberellic acid on sugarcane plants exposed to salinity under a hydroponic system. Afr. J. Plant Sci..

[bib63] Sigma-Aldrich (2021). Gibberellin A_4_. https://www.sigmaaldrich.com/catalog/product/sigma/g7276?lang=en&region=DK.

[bib64] Su P., Tong Y., Cheng Q., Hu Y., Zhang M., Yang J. (2016). Functional characterization of *ent*-copalyl diphosphate synthase, kaurene synthase and kaurene oxidase in the *Salvia miltiorrhiza* gibberellin biosynthetic pathway. Sci. Rep..

[bib65] Sun J., Zhang C., Nan W., Li D., Ke D., Lu W. (2019). Glycerol improves heterologous biosynthesis of betulinic acid in engineered *Yarrowia lipolytica*. Chem. Eng. Sci..

[bib66] Sun T.P., Kamiya Y. (1994). The Arabidopsis *GA1* locus encodes the cyclase *ent*-kaurene synthetase A of gibberellin biosynthesis. Plant Cell.

[bib67] Tai M., Stephanopoulos G. (2013). Engineering the push and pull of lipid biosynthesis in oleaginous yeast *Yarrowia lipolytica* for biofuel production. Metab. Eng..

[bib68] Tramontin L.R.R., Kildegaard K.R., Sudarsan S., Borodina I. (2019). Enhancement of astaxanthin biosynthesis in oleaginous yeast *Yarrowia lipolytica* via microalgal pathway. Microorganisms.

[bib69] Tudzynski B., Hedden P., Carrera E., Gaskin P. (2001). The P450-4 gene of *Gibberella fujikuroi* encodes *ent*-kaurene oxidase in the gibberellin biosynthesis pathway. Appl. Environ. Microbiol..

[bib70] Tudzynski B., Kawaide H., Kamiya Y. (1998). Gibberellin biosynthesis in *Gibberella fujikuroi*: cloning and characterization of the copalyl diphosphate synthase gene. Curr. Genet..

[bib71] Tudzynski B., Mihlan M., Rojas M.C., Linnemannstöns P., Gaskin P., Hedden P. (2003). Characterization of the final two genes of the gibberellin biosynthesis gene cluster of *Gibberella fujikuroi*. J. Biol. Chem..

[bib72] Tudzynski B., Rojas M.C., Gaskin P., Hedden P. (2002). The gibberellin 20-oxidase of *Gibberella fujikuroi* is a multifunctional monooxygenase. J. Biol. Chem..

[bib73] Tuna A.L., Kaya C., Dikilitas M., Higgs D. (2008). The combined effects of gibberellic acid and salinity on some antioxidant enzyme activities, plant growth parameters and nutritional status in maize plants. Environ. Exp. Bot..

[bib74] Wiemann P., Sieber C.M.K., von Bargen K.W., Studt L., Niehaus E.M., Espino J.J. (2013). Deciphering the cryptic genome: genome-wide analyses of the rice pathogen *Fusarium fujikuroi* reveal complex regulation of secondary metabolism and novel metabolites. PLoS Pathog..

[bib75] Williams J., Phillips A.L., Gaskin P., Hedden P. (1998). Function and substrate specificity of the gibberellin 3β-hydroxylase encoded by the *Arabidopsis* GA4 gene. Plant Physiol..

[bib76] Wittwer S.H., Bukovac M.J., Sell H.M., Weller L.E. (1957). Some effects of gibberellin on flowering and fruit setting. Plant Physiol..

[bib77] Yamaguchi S., Sun T., Kawaide H., Kamiya Y. (1998). The *GA2* locus of *Arabidopsis thaliana* encodes *ent*-kaurene synthase of gibberellin biosynthesis. Plant Physiol..

[bib78] Yang X., Nambou K., Wei L., Hua Q. (2016). Heterologous production of α-farnesene in metabolically engineered strains of *Yarrowia lipolytica*. Bioresour. Technol..

[bib79] Zang Y.X., Chun I.J., Zhang L.L., Hong S.B., Zheng W.W., Xu K. (2016). Effect of gibberellic acid application on plant growth attributes, return bloom, and fruit quality of rabbiteye blueberry. Sci. Hortic..

[bib80] Zhang C., Ju H., Lu C.-Z., Zhao F., Liu J., Guo X. (2019). High-titer production of 13*R*-manoyl oxide in metabolically engineered *Saccharomyces cerevisiae*. Microb. Cell Factories.

[bib81] Zhu Q., Jackson E.N. (2015). Metabolic engineering of *Yarrowia lipolytica* for industrial applications. Curr. Opin. Biotechnol..

