# Supplementary material for: Tailored biosynthesis of gibberellin plant hormones in yeast
Source: Metab Eng. 2021 Jul;66:1–11. doi: 10.1016/j.ymben.2021.03.010 (PMC8205117; doi:10.1016/j.ymben.2021.03.010)
Supplement: Multimedia component 1 [file mmc1.docx]

**Supplementary Information**

**Tailored biosynthesis of gibberellin plant hormones in yeast**

Kanchana R. Kildegaard^a,1^, Jonathan A. Arnesen^a,1^, Belén Adiego Pérez^a^, Daniela Rago^a^, Mette Kristensen^a^, Andreas K. Klitgaard^a^, Esben H. Hansen^b^, Jørgen Hansen^b^, Irina Borodina^a,^*

^a^ The Novo Nordisk Foundation Center for Biosustainability, Technical University of Denmark, Kemitorvet 220, 2800 Kgs. Lyngby, Denmark

^b^ River Stone Biotech, Fruebjergvej 3, 2100 København Ø, Denmark

^1^ Equal contribution

* Corresponding Author: Irina Borodina, The Novo Nordisk Foundation Center for Biosustainability, Technical University of Denmark, Kemitorvet 220, 2800 Kgs. Lyngby, Denmark. Tel: +45 2179 8657. E-mail: [irbo@biosustain.dtu.dk](mailto:irbo@biosustain.dtu.dk)

**Supplementary methods**

**Analytical methods**

The quantitative LC-MS data for the feeding experiments involving ST8504, control strain ST3683, and cell-free media was collected on EVOQ EliteTriple Quadrupole Mass Spectrometer system coupled with an Advance UHPLC pump (Bruker, Fremont Ca). The detection limit was 0.05 mg/L for all quantified gibberellins. Samples were held in the CTC HTS PAL autosampler at a temperature of 5.0°C during the analysis. Injection of 1 μL of the sample were made onto a Waters ACQUITY HSS T3 C18 UHPLC column, with the following dimensions; 1.8 um particle size, 2.1 mm i.d. and a length of 100 mm. The column was held at a temperature of 35.0°C. The solvent system used was Solvent A: MilliQ water with 0.1% formic acid and Solvent B: Acetonitrile with 0.1% formic acid. The Flow Rate was 0.400 mL/min with an initial solvent composition of %A = 90, %B = 10 held until 0.50 min, the solvent composition was then changed following a linear gradient until it reached %A = 5.0 and %B = 95.0 at 1.00 min. This was held until 4.5 min when the solvent was returned to the initial conditions and the column was re-equilibrated until 6.00 min. The column eluent flowed directly into the Heated ESI probe of the MS, which was held at 200°C, and a voltage of 2500 V. MRM Data was collected in negative ion mode and the target masses are shown in Supplementary Table S1. The MS settings were as follows, sheath gas flow rate of 50 units, nebulizer gas flow rate of 50 units, cone gas flow rate of 20 units, cone temp was 350°C, and collision gas pressure 1 mTorr. Authentic gibberellin standards were purchased from OlChemIm S.R.O. (Czech Republic).

All other LC-MS analyses of feeding experiments including control ST3683, KA-quantification, GA-quantification for ST6513 and ST6514, and GA_12_ mass confirmation were conducted on a Waters ACQUITY UPLC® (Waters Corporation) with a Waters ACQUITY UPLC® BEH C18 column (2.1 x 50 mm, 1.7 µm particles, 130 Å pore size) equipped with a pre-column (2.1 x 5 mm, 1.7 µm particles, 130 Å pore size) coupled to a Waters ACQUITY SQD2 single quadropole mass spectrometer with electrospray ionization (ESI) operated in negative ionization mode.

Compound separation was achieved using a gradient of the two mobile phases: Phase A (water with 0.1% formic acid) and phase B (ACN with 0.1% formic acid) were separated by increasing from 10% to 52.5% B from 0.0 to 3.0 minutes, increasing to 100% B after 5.0 minutes and holding 100% B for 0.3 minutes, and re-equilibrating for 0.3 minutes. The flow rate was 0.6 mL/min, and the column temperature was set at 55°C. The flow from the LC was split 1:3 before being introduced to the MS.

The MS was operated using the following settings: Capillary voltage -2500 V, cone voltage -38 V, desolvation temperature 300°C, and source temperature 150°C. Gibberellins were monitored using SIR (Single Ion Recording) and quantified by comparing against authentic standards. The following SIR traces were monitored: 301.2, 315.2, 317.2, 329.1, 331.2, 333.2, 345.2, 347.2, 361.2, 363.2, and 377.2 m/z.

Authentic gibberellin and *ent*-kaurenoic acid standards were purchased from OlChemIm S.R.O. (Czech Republic).

**Supplementary figures**


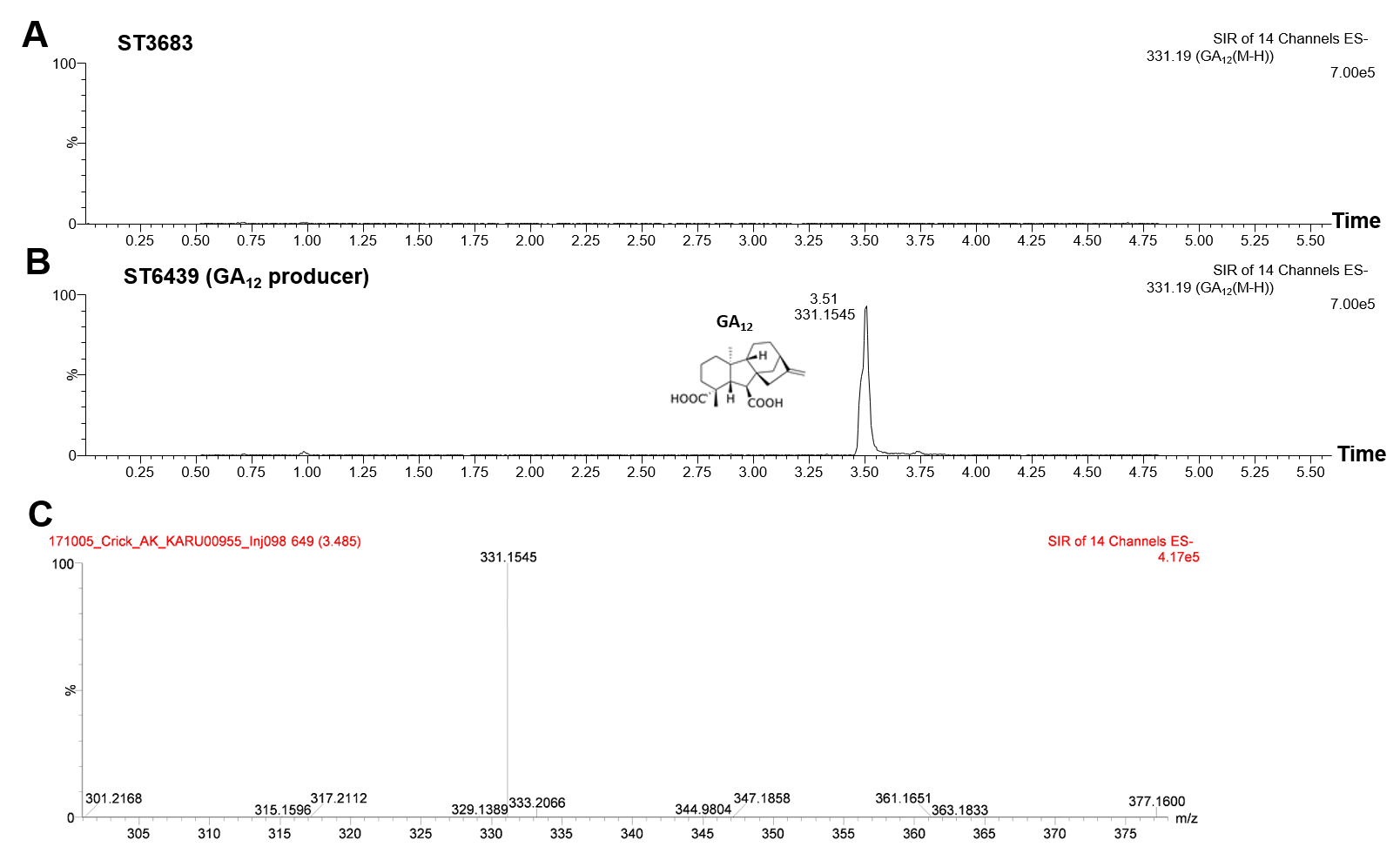


Supplementary Figure 1: LC-MS analysis of ST3683 and ST6439. A. Selected ion recording (SIR) extracted chromatogram of ST3683. B. SIR-extracted chromatogram of ST6439. C. mass-spectrometric fragmentation pattern of putative GA_12_ from ST6439.


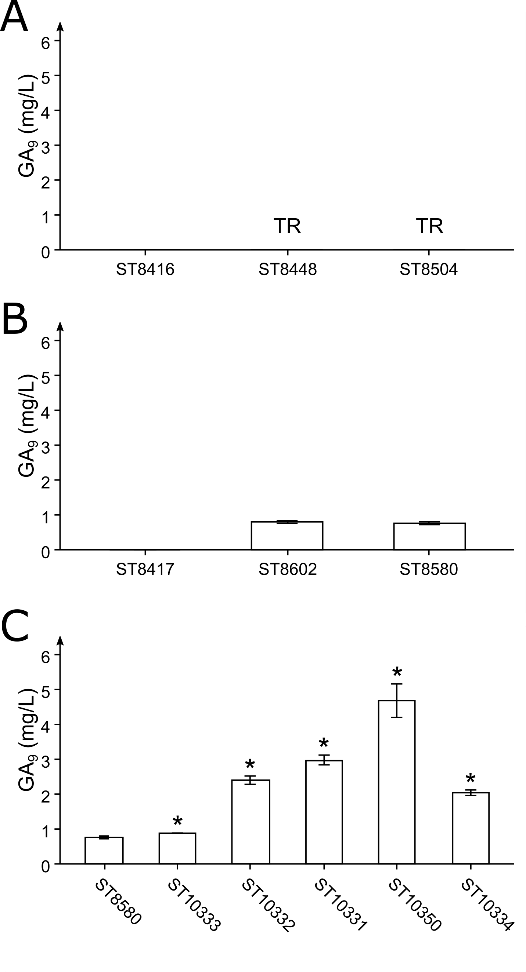


Figure 2: GA_9_-production by engineered Y. lipolytica strains **A.** GA_9_-production for ST8416, ST8448, and ST8504. **B.** GA_9_-production for ST8417, ST8602, and ST8580. **C.** GA_9_-production for ST8580, ST10333, ST10332, ST10331, ST10350, and ST10334. TR, trace amounts. Three biological replicates were used to calculate titer averages and standard deviations for all strains. Statistical significance (two tailed student’s t-test) compared to ST8580 is represented by asterisks (*, p < 0.05).


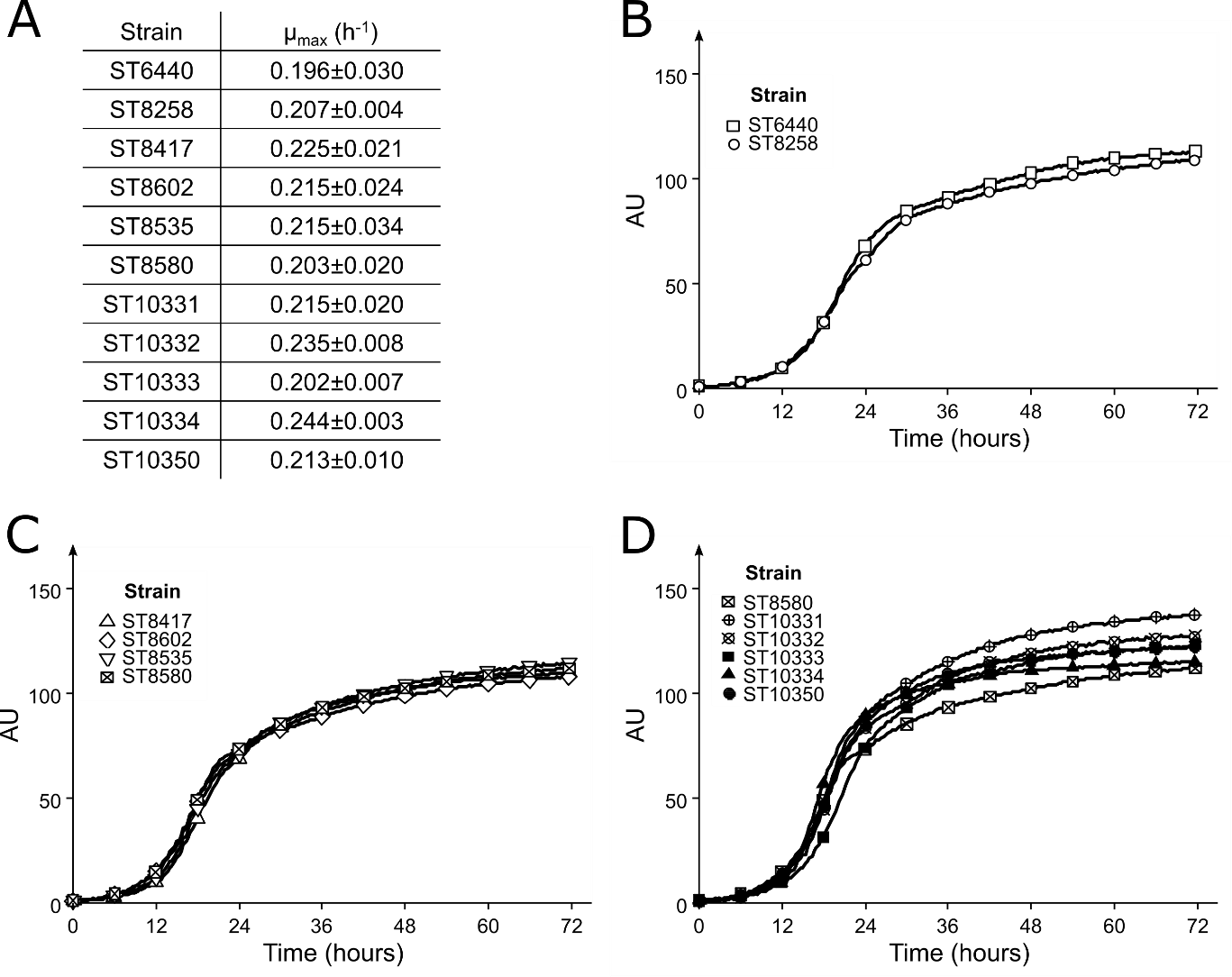


Figure 3: Growth profiles of engineered Y. lipolytica strains. **A.** Strain overview with μ_max_-values and standard deviations. **B.** Growth profiles of ST6440 and Cas9-expressing ST8258. **C.** Growth profiles of ST8417, ST8602, ST8535, and ST8580. **D.** Growth profiles of ST8580, ST10331, ST10332, ST10333, ST10334, and ST10350. Each growth profile represents the average of three biological replicates. AU, arbitrary units.


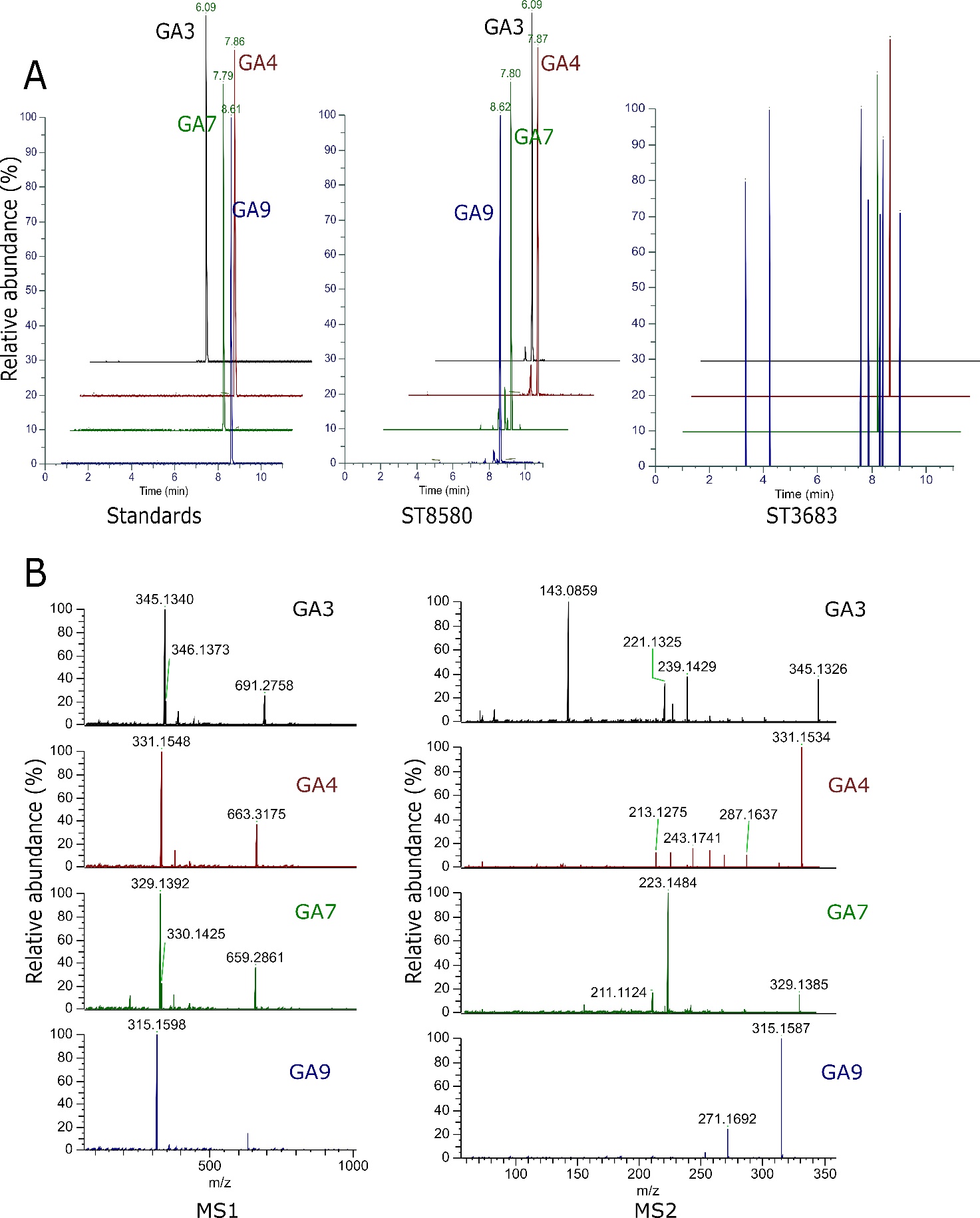


Figure 4. **A.** LC-MS chromatograms of authentic standards, GA-producer ST8580, and control strain ST3683. **B.** Chromatogram of GA3, GA4, GA7, and GA9. MS1, full scan. MS2, fragmentation pattern.

**Supplementary tables**

**Supplementary table 1**. Transitions for the gibberellins analyzed. 1. Transition was used as quantifier transition, 2. Transition was used as qualifier.

| Compound | Rt, min | Transition | Collision energy (V) |
| --- | --- | --- | --- |
| Gibberelic acid | 1.57 | 1. 345.6→239.5  2. 345.6→142.6 | 15  20 |
| Gibberellin A14 | 1.78 | 1. 347.5→303.6  2. 347.5→285.4 | 35  35 |
| Gibberellin A4 | 1.82 | 1. 331.3→256.8  2. 331.3→212.2 | 20  30 |
| Gibberellin A7 | 1.82 | 1. 329.6→223.5  2. 329.6→155.5 | 15  20 |
| Gibberellin A9 | 2.01 | 1. 315→270.8  2. 315→251.9 | 20  30 |
| Kaurenoic acid | 3.65 | 1. 301.3→301.3 | 8 |

**Supplementary table 2. Feeding experiment.** LC-MS results of *Yarrowia lipolytica* cultures fed with either GA_9_, GA_4_ or GA_7_.

|  | **strain** | **GA_9_** | **GA_4_** | **GA_7_** | **GA_3_** |
| --- | --- | --- | --- | --- | --- |
| **No feeding** | No strain | nd | nd | nd | nd |
|  | ST3683 | nd | nd | nd | nd |
|  | ST6514 | 6403 ± 584 | nd | nd | nd |
| **GA_9_ feeding** | ST3683 | 2304608 ± 1102401 | nd | nd | nd |
|  | ST6514 | 2809978 ± 98039 | 3468 ± 1280 | nd | nd |
| **GA_4_ feeding** | ST3683 | 18912 ± 1527 | 2016045 ± 68749 | 33893 ± 1934 | nd |
|  | ST6514 | 3915 ± 458 | 2101398 ± 51175 | 33672 ± 785 | nd |
| **GA_7_ feeding** | ST3683 | nd | 81235 ± 6597 | 1498320 ± 68730 | nd |
|  | ST6514 | nd | 85175 ± 702 | 1560660 ± 14448 | nd |

Values represent area under curve (AUC), arbitrary units.

nd, not detected.

**Supplementary table 3. Feeding experiment.** LC-MS results of *Yarrowia lipolytica* cultures fed with either GA_4_ or GA_7_.

|  | **strain** | **GA_9_** | **GA_4_** | **GA_7_** | **GA_3_** |
| --- | --- | --- | --- | --- | --- |
| **No feeding** | No strain | nd | nd | nd | nd |
|  | ST3683 | nd | nd | nd | nd |
|  | ST8504 | nd | 72568 ± 11245 | nd | nd |
| **GA_4_ feeding** | No strain | 85053 ± 1151 | 1764333 ± 68135 | 722247 ± 31753 | nd |
|  | ST3683 | 91621 ± 4833 | 1900333 ± 65501 | 843638 ± 1636 | nd |
| **GA_7_ feeding** | No strain | nd | 103829 ± 1351 | 24256667 ± 633114 | nd |
|  | ST3683 | nd | 107049 ± 4154 | 24926667 ± 1261600 | nd |
|  | ST8504 | nd | 102061 ± 2137 | 24866667 ± 237978 | nd |

Values represent area under curve (AUC), arbitrary units. No strain, YP+8%D without inoculation.

nd, not detected.

**Supplementary Methods**

**Supplementary Table S4. List of Primers used in this study.** Underlined sequences represent overhangs used for USER cloning.

| **Primer Name** | **Sequence (5'->3')** |
| --- | --- |
| NB326URA3fwdU | AGAACAGCUGAAGCTTCGTACG |
| NB327URA3Rev2U | AGGCCACUAGTGGATCTGATATCAC |
| PR-10593 (Fragment2EpiVecYL _fw) | AGCAGGCTUGGAGGCGACGTGGCAG |
| PR-10595 (PrTefYL _fw) | CGTGCGAUAGAGACCGGGTTGG |
| PR-10596 (PrTefYL _rev) | ATTTTGAAUGATTCTTATACTCAGAAG |
| PR-10599 (TefYL terminator _fw) | ACCCCACGUTGCCGGTCTTGCCTC |
| PR-10600 (TefYL terminator _rev) | CACGCGAUGAATTCGGACACGGGCATC |
| PR-10604 (tracrRNA _rev) | CACGCGAUACCGTACCCACACAAAAAAAGCACCACCGACTC |
| PR-10607 (PrtRNAGly _fw) | CGTGCGAUAGTGAATCATTGCTAACAGATC |
| PR-14149 (TPex20-Tlip2_rev) | AGCTGTTCUCAGATGCATTCTTGGGCGGTC |
| PR-14595 (IntA-1_DW_fw) | AGTGGCCUGGGGCTGGCGTGTGAAGGAG |
| PR-14785 (Cas9-start_fw) | ATTCAAAAUGGACAAGAAGTACTCTATC |
| PR-14786 (Cas9-start_rev) | AGTTTCGGUTGGCGAAGCCGTCAGAC |
| PR-14787 (Cas9-end_fw) | ACCGAAACUTCATGCAGCTGATC |
| PR-14788 (Cas9-end_rev) | ACGTGGGGUTAGACCTTTCGCTTCTTC |
| PR-15508 (PrTef_rev) | ATGACAGAUTTTGAATGATTCTTA |
| PR-15521 (PrExp_fw) | CGTGCGAUAAGGAGTTTGGCGCCCGTT |
| PR-15522 (PrExp_rev) | ATGACAGAUTGCTGTAGATATGTCTTGT |
| PR-15523 (PrYlFBA1 _fw) | CGTGCGAUAACAGTGTACGCAGTACTATAGAGG |
| PR-15524 (PrYlFBA1 _rev) | ATGACAGAUTGTGTGATGTGTAGTTTAGATTTCG |
| PR-15525 (PrYlFBA1for fusion_fw) | AGCTACTGAUAACAGTGTACGCAGTACTATAGAGG |
| PR-15528 (PGpd_rev) | ATGACAGAUTGTTGATGTGTGTTTAATTCAAGAATG |
| PR-15529 (PrYlGpdfor fusion_fw) | AGCTACTGAUGACGCAGTAGGATGTCCTGCACGG |
| PR-15585 (YlCYB5_U1_fw) | AGTGCAGGU GCCACA ATGATCATCAACGGCAAGGT |
| PR-15586 (YlCYB5_U1_rv) | CGTGCGAU TTATTTCTGACCCTGGAGGT |
| PR-15600 (GfCPS/KS_U2_fw) | ATCTGTCAU GCCACA ATGCACATCCTGACCTAC |
| PR-15601 (GfCPS/KS_U2_rv) | CACGCGAU TTACTTCATCGAAGAGGACAG |
| PR-15602 (AtGA2_U2_fw) | ATCTGTCAU GCCACA ATGTCGATCAACCTGCGA |
| PR-15603 (AtGA2_U2_rv) | CACGCGAU TTAGGTCAGAGATTCCTTCTGC |
| PR-15604 (PrExp_for fusion->_U2_fw) | AGCTACTGAUAAGGAGTTTGGCGCCCGTT |
| PR-15605 (<-PrExp_for fusion_U1_fw) | ATCAGTAGCUAAGGAGTTTGGCGCCCGTT |
| PR-15606 (<-PrExp_for fusion_U1_rv) | ACCTGCACUTGCTGTAGATATGTCTTGT |
| PR-15788 (PrtRNA-Gly_rev) | TAACCAACCUGCGCCGACCCGGAATCGAAC |
| PR-15789 (crRNA-TRPR_fw) | GTTTTAGAGCUAGAAATAGCAAGTTAAAATAAG |
| PR-17069 (GfP450-2_U1_fw) | AGTGCAGGU GCCACA ATGTCCATCTTCAACATGATC |
| PR-17070 (GfP450-2_U1_rv) | CGTGCGAU TTAGATGGCCTCGAGGTC |
| PR-17071 (GfDES_U2_fw) | ATCTGTCAU GCCACA ATGCCCCACAAGGACAAC |
| PR-17072 (GfDES_U2_rv) | CACGCGAU TTACCAGAAAGCAATGAAC |
| PR-17073 (GfP450-3_U1_fw) | AGTGCAGGU GCCACA ATGAAGTACACCACCTGT |
| PR-17074 (GfP450-3_U1_rv) | CGTGCGAU TTATCGTCGTCGCATCTC |
| PR-17075 (AtGA5_U1_fw) | AGTGCAGGU GCCACA ATGGCCGTGTCTTTCGTG |
| PR-17076 (AtGA5_U1_rv) | CGTGCGAU TTAGATAGGCTTGGTCAG |
| PR-17077 (AtGA3OX1_U2_fw) | ATCTGTCAU GCCACA ATGCCCGCCATGCTGACC |
| PR-17078 (AtGA3OX1_U2_rv) | CACGCGAU TTATTCCTCTCGGTGGTTTC |
| PR-17079 (GfCPR_U2_fw) | ATCTGTCAU GCCACA ATGGCCGAGCTGGACACC |
| PR-17080 (GfCPR_U2_rv) | CACGCGAU TTAAGACCACACGTCCTC |
| PR-17085 (GfP450-1_U2_fw) | ATCTGTCAU GCCACA ATGGCCAACCACTCTTCT |
| PR-17086 (GfP450-1_U2_rv) | CACGCGAU TCAGATGGCGATCTCCTC |
| PR-17990 (PrExp_U1_fw) | CACGCGAUAAGGAGTTTGGCGCCCGTT |
| PR-17991 (PrExp_U1_rv) | ACCTGCACU TGCTGTAGATATGTCTTGT |
| PR-18046 (AtATR2_U2_fw) | ATCTGTCAU GCCACA ATGTCCTCTTCGTCGTCC |
| PR-18047 (AtATR2_U2_rv) | CACGCGAU TCACCACACATCTCGCAG |
| PR-18048 (GfP450-4_U1_fw) | AGTGCAGGU GCCACA ATGTCCAAGTCCAACTCC |
| PR-18049 (GfP450-4_U1_rv) | CGTGCGAU TTACTCATCTCGGAGAG |
| PR-18050 (AtGA3_U1_fw) | AGTGCAGGU GCCACA ATGGCCTTCTTCTCCATG |
| PR-18051 (AtGA3_U1_rv) | CGTGCGAU TTAAGATCGTCGGGGGTT |
| PR-18214 (PTefintron_USER_rv) | AGTACTGCAAAAAGUGCTG |
| PR-18239 (gRNA3_IntC_3_sense) | AATAGTGCTATGGCTTTGGGGTTTTAGAGCT |
| PR-18240 (gRNA3_IntC_3_antisense) | CCCAAAGCCATAGCACTATTTAACCAACCT |
| PR-18241 (gRNA2_IntD_1_sense) | GCAGACGCAGACGGAGACTGGTTTTAGAGCT |
| PR-18242 (gRNA2_IntD_1_antisense) | CAGTCTCCGTCTGCGTCTGCTAACCAACCT |
| PR-18253 (gRNA3_IntE_3_sense) | CCTTGAATCAGTCGATGGTCGTTTTAGAGCT |
| PR-18254 (gRNA3_IntE_3_antisense) | GACCATCGACTGATTCAAGGTAACCAACCT |
| PR-18706 (PrYlFBA1for fusion_U1_fw) | ATCAGTAGCUAACAGTGTACGCAGTACTATAGAGG |
| PR-18707 (PrYlFBA1 _U1_rv) | ACCTGCACUTGTGTGATGTGTAGTTTAGATTTCG |
| PR-18708 (SsGGPS7_U2_fw) | ATCTGTCAU GCCACA ATGGTCGCACAAACTTTC |
| PR-18709 (SsGGPS7_U2_rv) | CACGCGAU TTAATGCTGACGACGTGTG |
| PR-18714 (GfCyb5_U1_fw) | AGTGCAGGU GCCACA ATGTCTGCCAAGAAGGAA |
| PR-18715 (GfCyb5_U1_rv) | CGTGCGAU TTAGGCAGAGGCTTGGCC |
| PR-18716 (GfP450-3UP_U1_fw) | AGTGCAGGU GCCACA ATGATGTCTAACTTCGTGA |
| PR-18717 (GfP450-3UP_U1_rv) | AGGCCACUGGTCAGAGGCG |
| PR-18718 (GfP450-3Down_U1_fw) | AGTGGCCUCGAACCTCTAAG |
| PR-18719 (GfCPR_new_U2_rv) | CACGCGAUTTAAGACCACACGTCCTCTTGGTACTGGTTGGCAGATCG |
| PR-18720 (GfCPS_KS_new_U2_fw) | ATCTGTCAUGCCACAATGCCCGGCAAGATCGAGAACGGC |
| PR-18721 (SsGGPS7_U1_fw) | AGTGCAGGU GCCACA ATGGTCGCACAAACTTTC |
| PR-18722 (SsGGPS7_U1_rv) | CGTGCGAU TTAATGCTGACGACGTGTG |
| PR-18930 (PrTEF1) | ATCAGTAGCU AGAGACCGGGTTGG |
| PR-18931 (PrTEF1 ->_forfusion_U2_fw) | AGCTACTGAU AGAGACCGGGTTGG |
| PR-18977 (PrTef->_fw) | AGCTACTGAUAGAGACCGGGTTGGCGGCG |
| PR-21844 (gRNA_opened_rv) | ATCAGTAGCUCTACTGTATATTCAAGCAAG |
| PR-21915 (TPOT1->_forfusion_U3_fw) | ACTTACAAU ATGTACATACAAGATTATTTATAG |
| PR-21916 (TPOT1->_forfusion_U3_rv) | ATCAGTAGCU ACCGCTCGACAACCTCCC |
| PR-21919 (GfCybRed_U2_fw) | ATCTGTCAU GCCACA ATGTCCTCTAACGGCGAC |
| PR-21920 (GfCybRed_U2_rv) | ATTGTAAGU TTAGAAGGCGAACACCTG |
| PR-21921 (AtGA5_forfusionPTefin_U1_fw) | ACTTTTTGCAGTACUAACCGCAG GCCGTGTCTTTCGTGACC |
| PR-21922 (AtGA3ox_forfusionPTef_U2_fw) | ACTTTTTGCAGTACUAACCGCAG CCCGCCATGCTGACCGAC |
| PR-22679 (TLeu2_U_rv) | AAGCCTGCUTGTCATGCCTACAACTCATATAC |
| PR-26761 (GfKO_F) | ATCTGTCAUGCCACAATGTCCAAGTCCAACTCCATG |
| PR-26762 (GfKO_R) | CGTGCGAUTTACTCATCTCGGAGAGATCG |
| PR-26763 (GfCPS/KS_F) | ACTTTTTGCAGTACUAACCGCAGCCCGGCAAGATCGAGAACG |
| PR-26764 (GfCPS/KS_R) | CACGCGAUTTACTTCATCGAAGAGGACAGG |
| PR-26765 (AtKS_gpd_F) | ATCTGTCAUGCCACAATGTCGATCAACCTGCGATCT |
| PR-26766 (AtKS_gpd_R) | CGTGCGAUTTAGGTCAGAGATTCCTTCTGC |
| PR-26767 (AtCPS_tefint_F) | ACTTTTTGCAGTACUAACCGCAGTCTCTGCAGTACCACGTGC |
| PR-26768 (AtCPS_tefint_R) | AAATCACTACTCUTTACACCTTCTGGAACAGCA |
| PR-26769 (Tpot1_F) | AGAGTAGTGATTUATGTACATACAAGATTATTTATAGAA |
| PR-26770 (Tpot1_R) | AGACACTCTTACUACCGCTCGACAACCTCC |
| PR-26771 (PrExp_Tpot1_F) | AGTAAGAGTGTCUAAGGAGTTTGGCGCCCGTT |
| PR-26772 (AtKO_R) | CACGCGAUTTAAGATCGTCGGGGGTTGA |
| PR-26773 (tAtKS_F) | ATCTGTCAUGCCACAATGATGCTCGAGAAGGTCGAG |
| PR-26775 (PrExp_R) | ACCTGCACUTGCTGTAGATATGTCTTGTGTG |
| PR-26776 (tAtKO_F) | AGTGCAGGUGCCACAATGTCTTTCTCGCGAAAGAACATG |
| PR-26777 (tAtKO_gpd_F) | ATCTGTCAUGCCACAATGTCTTTCTCGCGAAAGAACAT |
| PR-26778 (tAtKO_gpd_R) | CGTGCGAUTTAAGATCGTCGGGGGTT |
| PR-26779 (tAtCPS_link_R) | ACCGCCCACCUTCTGGAACAGCACCTTAGAG |
| PR-26780 (tAtKS_link_F) | AGGTGGGCGGUGGCTCTATGCTCGAGAAGGTCGAGC |
| PR-26781 (tAtKS_Tlip2_R) | CACGCGAUTTAGGTCAGAGATTCCTTCTGC |
| PR-26782 (AtKO_gpd_F) | ATCTGTCAUGCCACAATGGCCTTCTTCTCCATGATC |
| PR-26783 (AtKS_link_F) | AGGTGGGCGGUGGCTCTATGTCGATCAACCTGCGATC |
| PR-26867 (tAtCPS_V2_F) | ACTTTTTGCAGTACUAACCGCAGTCTCAAGAGGTGCAGCACGACCTGC |
| PR-26955 (AtCPS_tefint_2_R) | TCTATAAATAATCTTGTATGTACATAAATCACTACTCTTTACACCTTCTGGAACAGCACC |
| PR-26956 (Tpot1_2_F) | CTGTTCCAGAAGGTGTAAAGAGTAGTGATTTATGTACATACAAGATTATTTATAGAAATG |
| PR-26959 (PrExp_2_R) | GACATGTTCTTTCGCGAGAAAGACATTGTGGCACCTGCACTTGCTGTAGATATGTCTTGT |
| PR-26960 (tAtKO_2_F) | TACACACAAGACATATCTACAGCAAGTGCAGGTGCCACAATGTCTTTCTCGCGAAAGAAC |
| PR-27422 (Yl_USER-backbone1_rev) | ATCGCACGCU TT CCG TTG AAG TGT GGA TGG GGA AGT GAG T |
| PR-27423 (Yl_USER-backbone2_fw) | ATCGCGTGCAU TCCTTCTGTTCGGAATCAACCTCAAGG |

**Supplementary Table S5. List of Biobricks used in this study**

| **Biobrick Name** | **Template** | **Forward primer** | **Reverse primer** |
| --- | --- | --- | --- |
| BB0993 (PTef1->) | ST3683 | PR-10596 | PR-10595 |
| BB0995 (TTef->) | ST3683 | PR-10600 | PR-10599 |
| BB01541 (Cas9_start) | p4904 (Cas9_YLopti_1), | PR-14786 | PR-14785 |
| BB01542 (Cas9_end) | p4905 (Cas9_YLopti_2), | PR-14788 | PR-14787 |
| BB1558 (PrExp->) | ST3683 | PR-15522 | PR-15521 |
| BB01559 (PFba1->) | ST3683 | PR-15524 | PR-15523 |
| BB01560 (PFba1->_for fusion) | ST3683 | PR-15524 | PR-15525 |
| BB01563 (PGpd1->_for fusion) | ST3683 | PR-15528 | PR-15529 |
| BB01620 (PExp_for fusion->) | ST3683 | PR-15522 | PR-15604 |
| BB01621 (<-PExp_for fusion) | ST3683 | PR-15606 | PR-15605 |
| BB01628 (AtGA2->) | pCfB5227 | PR-15603 | PR-15602 |
| BB1635 (PrtRNA-Gly) | pCfB4589 | PR-15788 | PR-10607 |
| BB1636 (crRNA-TRPR) | pCfB4589 | PR-10604 | PR-15789 |
| BB01901 (GfP450-2<-) | pCfB5868 | PR-17070 | PR-17069 |
| BB01902 (GfDES->) | pCfB5873 | PR-17072 | PR-17071 |
| BB01904 (AtGA5<-) | pCfB5870 | PR-17076 | PR-17075 |
| BB01905 (AtGA3OX1->) | pCfB5872 | PR-17078 | PR-17077 |
| BB01909 (GfP450-1->) | pCfB5228 | PR-17086 | PR-17085 |
| BB01911 (YlCYB5<-) | ST3683 | PR-15586 | PR-15585 |
| BB02016 (PrExp<-) | ST3683 | PR-17991 | PR-17990 |
| BB02054 (AtATR2->) | pCfB5180 | PR-18047 | PR-18046 |
| BB02055 (GfP450-4<-) | pCfB5179 | PR-18049 | PR-18048 |
| BB02056 (AtGA3<-) | pCfB5178 | PR-18051 | PR-18050 |
| BB02080 (TLip2-Hph_cass-IntE_4-DW), | pCfB5929 (4 kb SacI-HpaI fragment) |  |  |
| BB02141 (PFba1<-_for fusion) | ST3683 | PR-18707 | PR-18706 |
| BB02142 (SsGGPPS7yl) | pCfB6778 | PR-18709 | PR-18708 |
| BB02145 (GfCyb5<-) | pCfB6781 | PR-18714 | PR-18714 |
| BB02146 (GfP450-3UP<-) | pCfB6782 | PR-18716 | PR-18716 |
| BB02147 (GfP450-3Down<-) | pCfB5869 | PR-18718 | PR-18718 |
| BB02148 (GfCPR_new->) | pCfB5872 | PR-18719 | PR-17079 |
| BB02149 (GfCPS/KS_New->) | pCfB5203 | PR-15601 | PR-18720 |
| BB2171 (PTef_forfusion->) | ST3683 | PR-15508 | PR-18931 |
| BB02174 (<-PrExp-PrGpd1->) | pCfB6793 | PR-15528 | PR-15606 |
| BB02210 ( PrTefintron_USER_forfusion <-) | ST3683 | PR-18214 | PR-18930 |
| BB03106 (GfP450-3<-PFba1<-) | pCfB6794 | PR-18706 | PR-17074 |
| BB02211 ( PTefintron_forfusion->) | ST3683 | PR-18977 | PR-18214 |
| BB2731 (Tpot->) | ST3683 | PR-21916 | PR-21915 |
| BB02745 (gRNA backbone_p6630) | pCfB6630 | PR-21844 | PR-10593 |
| BB03107 (PExp->GfDES->) | pCfB6794 | PR-15604 | PR-17072 |
| BB02813 (<-PExp-PTefintron->) | BB02211, BB1621 | PR-15606 | PR-18214 |
| BB02827 (GfCybRed_U3->) | pCfB8066 | PR-21919 | PR-21920 |
| BB02829 (AtGA5_forfusion with PTefintron) | pCfB6793 | PR-17076 | PR-21921 |
| BB02830 (AtGA3ox_forfusion with Ptefin) | pCfB6793 | PR-17078 | PR-21922 |
| BB2985 (<-PExp-PGpd1-GfCybRed) | BB02174, BB02827 | PR-21920 | PR-15606 |
| BB03080 (PTefintron-YlLeu2-TLeu2) | pCfB6458 | PR-22679 | PR-18931 |
| BB3865 (<-PrGDP_Tefint->) | Described in (Arnesen *et al.*, 2020) |  |  |
| BB4417 (GfKO_gpd) | pCfB6600 (pIntE-4-Hph-GfP450-4<-PrExp) | 26761 | 26762 |
| BB4418 (GfCPS/KS_tefint) | pCfB6804 (pIntE-1-NatMx-PrExp->GfCPS/KS_new) | 26763 | 26764 |
| BB4419 (AtKS_gpd) | p05249 (pIntE-1-NatMx-AtGA1<-PrExp-PrGPD->AtGA2) | 26765 | 26766 |
| BB4420 (AtCPS_tefint) | p05249 (pIntE-1-NatMx-AtGA1<-PrExp-PrGPD->AtGA2) | 26767 | 26955 |
| BB4421 (Tpot1) | pCfB8092 (repair_template-,<-MaC16/18E<-PrExp-PrGPD->YlÄ9D->TEFintron->YlÄ12D->) | 26956 | 26770 |
| BB4422 (PrExp-AtKO) | pCfB6599 (pIntE-4-Hph-AtGA3<-PrExp) | 26771 | 26772 |
| BB4423 (AtKO_gpd) | pCfB6599 (pIntE-4-Hph-AtGA3<-PrExp) | 26782 | 26778 |
| BB4426 (tAtKS_gpd) | p05249 (pIntE-1-NatMx-AtGA1<-PrExp-PrGPD->AtGA2) | 26773 | 26766 |
| BB4428 (PrExp) | p05249 (pIntE-1-NatMx-AtGA1<-PrExp-PrGPD->AtGA2) | 26771 | 26959 |
| BB4429 (tAtKO) | pCfB6599 (pIntE-4-Hph-AtGA3<-PrExp) | 26960 | 26772 |
| BB4430 (tAtKO_gpd) | pCfB6599 (pIntE-4-Hph-AtGA3<-PrExp) | 26777 | 26778 |
| BB4433 (AtCPS-Tpot1) | BB4420 (AtCPS_tefint)+BB4421 (Tpot1) | 26767 | 26770 |
| BB4436 (PrExp-tAtKO) | BB4428 (PrExp)+BB4429 (tAtKO) | 26771 | 26772 |
| BB4634 (AtCPS_link_USER) | p05249 (pIntE-1-NatMx-AtGA1<-PrExp-PrGPD->AtGA2) | 26767 | 26779 |
| BB4635 (link-AtKS_USER) | p05249 (pIntE-1-NatMx-AtGA1<-PrExp-PrGPD->AtGA2) | 26783 | 26781 |
| BB4636 (tAtCPS_tefint_USER) | p05249 (pIntE-1-NatMx-AtGA1<-PrExp-PrGPD->AtGA2) | 26867 | 26768 |
| BB4637 (Tpot1_USER) | pCfB8092 (repair_template-,<-MaC16/18E<-PrExp-PrGPD->YlÄ9D->TEFintron->YlÄ12D->) | 26769 | 26770 |
| BB4638 (tAtCPS_link_USER) | p05249 (pIntE-1-NatMx-AtGA1<-PrExp-PrGPD->AtGA2) | 26867 | 26779 |
| BB4639 (link-tAtKS_USER) | p05249 (pIntE-1-NatMx-AtGA1<-PrExp-PrGPD->AtGA2) | 26780 | 26781 |
| BB4760 (AtKO<-PrGPD-Tefintron->AtCPS-link-AtKS) | pCfB10014 (pIntA-1-URA-TPex20-AtKO<-PrGPD-Tefintron->AtCPS-link-AtKS-TLip2_V2) | 14595 | 14149 |
| BB4761 (AtKS<-PrGPD-Tefintron->AtCPS-Tpot1-PrEXP->AtKO) | pCfB9673 (pIntA-1-URA-TPex20-AtKS<-PrGPD-Tefintron->AtCPS-Tpot1-PrEXP->AtKO-TLip2) | 14595 | 14149 |
| BB4762 (GfKO<-PrGPD-Tefintron->GfCPS/KS) | pCfB9674 (pIntA-1-URA-TPex20-GfKO<-PrGPD-Tefintron->GfCPS/KS-TLip2) | 14595 | 14149 |
| BB4763 (tAtKO<-PrGPD-Tefintron->tAtCPS-link-tAtKS) | pCfB10015 (pIntA-1-URA-TPex20-tAtKO<-PrGPD-Tefintron->tAtCPS-link-tAtKS-TLip2_V2) | 14595 | 14149 |
| BB4764 (tAtKS<-PrGPD-Tefintron->tAtCPS-Tpot1-PrEXP->tAtKO) | pCfB10016 (pIntA-1-URA-TPex20-tAtKS<-PrGPD-Tefintron->tAtCPS-Tpot1-PrEXP->tAtKO-TLip2_V2) | 14595 | 14149 |
| BB4765 (pIntA_1_vector_backbone_opened) | pCfB5935 (pIntA-1-HphMx-TPex20-TLip2) | 14595 | 14149 |
| BB4766 (URA-cass_from_4586) | pCfB4586 (pIntB-Ura-TPex20-TLip2) | 141 | 142 |
| BB4792 (URA3-cassette for Y. lipolytica) | ST6295 | 141 | 142 |
| BB4802 (pIntA1_URA3_functional_backbone) | pCfB10132 (pIntA-1-URA-TPex20-tAtKS<-PrGPD-Tefintron->tAtCPS-Tpot1-PrEXP->tAtKO-TLip2_v2) | 27423 | 27422 |
| BB4803 (Fragment_C) | pCfB10016 (pIntA-1-URA-TPex20-tAtKS<-PrGPD-Tefintron->tAtCPS-Tpot1-PrEXP->tAtKO-TLip2_V2) | 26766 | 26770 |
| BB4804 (Fragment_D) | pCfB10016 (pIntA-1-URA-TPex20-tAtKS<-PrGPD-Tefintron->tAtCPS-Tpot1-PrEXP->tAtKO-TLip2_V2) | 26776 | 26772 |
| BB4807 (PrEXP_fix_PL-10132) | p05249 (pIntE-1-NatMx-AtGA1<-PrExp-PrGPD->AtGA2) | 26771 | 26775 |

**Supplementary Table S6. List of Plasmids used in this study**

| **Plasmid ID** | **Plasmid Name** | **Parent plasmid** | **Biobricks** | **References** |  |
| --- | --- | --- | --- | --- | --- |
| pCfB3405 | pCfB3405 (pORI1001-Nat-CEN1-USER) |  |  | (Holkenbrink *et al.*, 2018) |  |
| pCfB3529 | pCfB3529 (pORI1001-LeuKl-CEN1-USER) |  |  | (Holkenbrink *et al.*, 2018) |  |
| pCfB4132 | pCfB4132 (pIntB-HphSyn-TPex20-TLip2) |  |  | (Holkenbrink *et al.*, 2018) |  |
| pCfB4158 | pCfB4158 (pLeu-PrExp-Cre) |  |  | (Holkenbrink *et al.*, 2018) |  |
| pCfB4572 | pCfB4572 (pIntF-Hph-TPex20-TLip2) |  |  | (Holkenbrink *et al.*, 2018) |  |
| pCfB4586 | pCfB4586 (pIntB-Ura-TPex20-TLip2) |  |  | (Holkenbrink *et al.*, 2018) |  |
| pCfB4589 | pCfB4589 (PrtRNA-Gly-gRNA_GFPNat) |  |  | (Holkenbrink *et al.*, 2018) |  |
| pCfB4589 | pCfB4589 (PrtRNA-Gly-gRNA_GFPNat) |  |  | (Holkenbrink *et al.*, 2018) |  |
| pCfB4666 | pCfB4666 (pIntE-1-Nat-TPex20-TLip2) |  |  | (Holkenbrink *et al.*, 2018) |  |
| pCfB4779 | pCfB4779 (pIntE_2-Nat-TPex20-TLip2) |  |  | (Holkenbrink *et al.*, 2018) |  |
| pCfB4782 | pCfB4782 (pIntC_2-Nat-TPex20-TLip2) |  |  | (Holkenbrink *et al.*, 2018) |  |
| pCfB4784 | pCfB4784 (pIntF_2-Nat-TPex20-TLip2) |  |  | (Holkenbrink *et al.*, 2018) |  |
| pCfB4904 | p4904 (Cas9_YLopti_1), |  |  | (Holkenbrink *et al.*, 2018) |  |
| pCfB4905 | p4905 (Cas9_YLopti_2), |  |  | (Holkenbrink *et al.*, 2018) |  |
| pCfB5122 | pCfB5122 (pIntB-HphMx-PrExp->hrGFP) |  |  | (Holkenbrink *et al.*, 2018) |  |
| pCfB5219 | pCfB5219 (pIntE-4-HphMx-TPex20-TLip2) |  |  | (Holkenbrink *et al.*, 2018) |  |
| pCfB5249 | pCfB5249 (pIntE-1-NatMx-AtGA1-AtGA2) | pCfB4666 | BB1563, BB1621, BB1628, pCfB5177 | This study |  |
| pCfB5921 | pCfB5921 (pIntF-HphMX-PrTEF1-Cas9-TTEF) | pCfB4572 | BB993, BB995, BB1541, BB1542 | This study |  |
| pCfB5935 | pCfB5935 (pIntA-1-HphMx-TPex20-TLip2) |  |  | (Holkenbrink *et al.*, 2018) |  |
| pCfB6063 | pCfB6063 (pIntE-4-Markerfree-AtKO-AtKAO2) | pCfB5251 | BB1563, BB1621, pCfB5178, pCfB5140 | This study |  |
| pCfB6371 | pCfB6371 (pIntC_3-TPex20-TLip2) |  |  | (Holkenbrink *et al.*, 2018) |  |
| pCfB6458 | pCfB6458 (pGPATnative_Leu2) |  |  | (Holkenbrink *et al.*, 2018) |  |
| pCfB6544 | pCfB6544 (pIntE-4-Markerfree-AtGA3) | pCfB5251 | BB2016, BB2056 | This study |  |
| pCfB6556 | pCfB6556 (pIntE-4-Markerfree-GfP450-4) | pCfB5251 | BB2016, BB2055 | This study |  |
| pCfB6563 | pCfB6563 (pIntC-2-Nat-YlCyb5-AtATR2) | pCfB4782 | BB1563, BB1621, BB1911, BB2054 | This study |  |
| pCfB6598 | pCfB6598 (pIntE-4-Hph-AtGA3-AtKAO2) | pCfB6063 (SacI-HpaI) | BB2080 | This study |  |
| pCfB6599 | pCfB6599 (pIntE-4-Hph-AtGA3) | pCfB6544 (SacI-HpaI) | BB2080 | This study |  |
| pCfB6600 | pCfB6600 (pIntE-4-Hph-GfP450-4} | pCfB6556 (SacI-HpaI) | BB2080 | This study |  |
| pCfB6630 | pCfB6630 (pNat-YLgRNA3_IntC_3) | pCfB3405 | BB1635, BB1636, PR-18239, PR-18240 | This study |  |
| pCfB6630 | pCfB6630 (pNat-YLgRNA3_IntC_3) | pCfB3405 | BB1635, BB1636, PR-18239, PR-18240 | This study |  |
| pCfB6631 | pCfB6631 (pNat-YLgRNA2_IntD_1) | pCfB3405 | BB1635, BB1636, PR-18241, PR-18242 | This study |  |
| pCfB6637 | pCfB6637 (pNat-YLgRNA3_IntE_3) | pCfB3405 | BB1635, BB1636, PR-18253, PR-18254 | This study |  |
| pCfB6681 | pCfB6681 (pIntE_3-TPex20-TLip2) |  |  | (Holkenbrink *et al.*, 2018) |  |
| pCfB6684 | pCfB6684 (pIntD_1-TPex20-TLip2) |  |  | (Holkenbrink *et al.*, 2018) |  |
| pCfB6792 | pCfB6792 (pIntF-2-Nat-GfP450-2) | pCfB4784 | BB2016, BB1901 | This study |  |
| pCfB6793 | pCfB6793 (pIntF-2-Nat-AtGA5-PExp-PGpd1-AtGA3OX1) | pCfB4784 | BB1621, BB1563, BB1904, BB1905 | This study |  |
| pCfB6794 | pCfB6794 (pIntA-1-Hph-GfP450-3-PFba1-PExp-GfDES) | pCfB5935 | BB2141, BB1620, BB2146, BB2147, BB1902 | This study |  |
| pCfB6804 | pCfB6804 (pIntE-1-NatMx-PrExp->GfCPS/KS_new) | pCfB4666 | BB1558, BB2149 | This study |  |
| pCfB6832 | pCfB6832 (pIntC-2-Nat- GfCyb5-GfCPR) | pCfB4782 | BB1621, BB1560, BB2145, BB2148 | This study |  |
| pCfB8092 | pCfB8092 (repair_template-,<-MaC16/18E<-PrExp-PrGPD->YlÄ9D->TEFintron->YlÄ12D->) |  |  | Provided by L. R. R. Tramontin. |  |
| pCfB8125 | pCfB8125 (pIntD_1-PFBA1-SsGGPPs7) | pCfB6684 | BB1559, BB2142 | This study |  |
| pCfB8141 | pCfB8141 (pIntE_3-GfCyb-GfCybRed-GfCPR) | pCfB6681 | BB2145, BB2985, BB2731, BB2171, BB2148 | This study |  |
| pCfB8246 | pCfB8246 (pIntC-3-AtGA5-PExp-PTefintron-AtGA3ox) | pCfB6371 | BB2813, BB1904, BB2830 | This study |  |
| pCfB8247 | pCfB8247 (pIntC-3-AtGA5-PTefintron-PGpd-AtGA3ox) | pCfB6371 | BB2210, BB1563, BB2829, BB1905 | This study |  |
| pCfB8299 | pCfB8299 (pIntE-2-NAT-GfP450-3-PFba1-PExp-GfDES) | pCfB4779 | BB3106, BB3107 | This study |  |
| pCfB8398 | pCfB8398 (pIntC-3-GfP450-2-PExp-PGpd-GfP450-1) | pCfB6371 | BB2174, BB1901, BB1909 | This study |  |
| pCfB8399 | pCfB8399 (pLeu2-YLgRNA3_IntC_3) |  | BB2745, BB3080 | This study |  |
| pCfB9673 | pCfB9673 (pIntA-1-URA-TPex20-AtKS<-PrGPD-Tefintron->AtCPS-Tpot1-PrEXP->AtKO-TLip2) | pCfB9856 (pIntA-1-URA3-TPex20-TLip2) | BB3865 (<-PrGDP_Tefint->), BB4419 (AtKS_gpd), BB4433 (AtCPS-Tpot1), BB4422 (PrExp-AtKO) | This study |  |
| pCfB9674 | pCfB9674 (pIntA-1-URA-TPex20-GfKO<-PrGPD-Tefintron->GfCPS/KS-TLip2) | pCfB9856 (pIntA-1-URA3-TPex20-TLip2) | BB3865 (<-PrGDP_Tefint->), BB4417 (GfKO_gpd), BB4418 (GfCPS/KS_tefint) | This study |  |
| pCfB9856 | pCfB9856 (pIntA-1-URA3-TPex20-TLip2) |  | BB4765 (pIntA_1_vector_backbone_opened), BB4766 (URA-cass_from_4586) | This study |  |
| pCfB10014 | pCfB10014 (pIntA-1-URA-TPex20-AtKO<-PrGPD-Tefintron->AtCPS-link-AtKS-TLip2_V2) | pCfB9856 (pIntA-1-URA3-TPex20-TLip2) | BB3865 (<-PrGDP_Tefint->), BB4423 (AtKO_gpd), BB4634 (AtCPS_link_USER), BB4635 (link-AtKS_USER) | This study |  |
| pCfB10015 | pCfB10015 (pIntA-1-URA-TPex20-tAtKO<-PrGPD-Tefintron->tAtCPS-link-tAtKS-TLip2_V2) | pCfB9856 (pIntA-1-URA3-TPex20-TLip2) | BB3865 (<-PrGDP_Tefint->), BB4430 (tAtKO_gpd), BB4638 (tAtCPS_link_USER), BB4639 (link-tAtKS_USER) | This study |  |
| pCfB10016 | pCfB10016 (pIntA-1-URA-TPex20-tAtKS<-PrGPD-Tefintron->tAtCPS-Tpot1-PrEXP->tAtKO-TLip2_V2) | pCfB9856 (pIntA-1-URA3-TPex20-TLip2) | BB3865 (<-PrGDP_Tefint->), BB4426 (tAtKS_gpd), BB4636 (tAtCPS_tefint_USER), BB4637 (Tpot1_USER), BB4436 (PrExp-tAtKO) | This study |  |
| pCfB10128 | pCfB10128 (pIntA-1-URA-TPex20-AtKO<-PrGPD-Tefintron->AtCPS-link-AtKS-TLip2_v2) |  | BB4760 (AtKO<-PrGPD-Tefintron->AtCPS-link-AtKS), BB4792 (URA3-cassette for Y. lipolytica) | This study |  |
| pCfB10129 | pCfB10129 (pIntA-1-URA-TPex20-AtKS<-PrGPD-Tefintron->AtCPS-Tpot1-PrEXP->AtKO-TLip2_v2) |  | BB4761 (AtKS<-PrGPD-Tefintron->AtCPS-Tpot1-PrEXP->AtKO), BB4792 (URA3-cassette for Y. lipolytica) | This study |  |
| pCfB10130 | pCfB10130 (pIntA-1-URA-TPex20-GfKO<-PrGPD-Tefintron->GfCPS/KS-TLip2_v2) |  | BB4762 (GfKO<-PrGPD-Tefintron->GfCPS/KS), BB4792 (URA3-cassette for Y. lipolytica) | This study |  |
| pCfB10131 | pCfB10131 (pIntA-1-URA-TPex20-tAtKO<-PrGPD-Tefintron->tAtCPS-link-tAtKS-TLip2_v2) |  | BB4763 (tAtKO<-PrGPD-Tefintron->tAtCPS-link-tAtKS), BB4792 (URA3-cassette for Y. lipolytica) | This study |  |
| pCfB10132 | pCfB10132 (pIntA-1-URA-TPex20-tAtKS<-PrGPD-Tefintron->tAtCPS-Tpot1-PrEXP->tAtKO-TLip2_v2) |  | BB4764 (tAtKS<-PrGPD-Tefintron->tAtCPS-Tpot1-PrEXP->tAtKO), BB4792 (URA3-cassette for Y. lipolytica) | This study |  |
| pCfB10135 | pCfB10135 (pIntA-1-URA-TPex20-tAtKS<-PrGPD-Tefintron->tAtCPS-Tpot1-PrEXP->tAtKO-TLip2_v2.2) |  | BB4802 (pIntA1_URA3_functional_backbone), BB4803 (Fragment_C), BB4804 (Fragment_D), BB4807 (PrEXP_fix_PL-10132) | This study |  |
| **Synthetic genes** | | | | | |
| pCfB5140 | p5140 (GeneUStringAtKAO2) |  |  | This study |  |
| pCfB5177 | p5177 (GeneUString AtGA1) |  |  | This study |  |
| pCfB5178 | p5178 (GeneUString AtGA3) |  |  | This study |  |
| pCfB5179 | p5179 (GeneUString GfP450-4) |  |  | This study |  |
| pCfB5180 | p5180 (GeneUString AtATR2) |  |  | This study |  |
| pCfB5203 | p5203 (GfCPS/KS) |  |  | This study |  |
| pCfB5227 | p5227 (AtGA2) |  |  | This study |  |
| pCfB5228 | p5228 (GfP450-1) |  |  | This study |  |
| pCfB5868 | p5868 (GfP450-2) |  |  | This study |  |
| pCfB5869 | p5869 (GfP450-3) |  |  | This study |  |
| pCfB5870 | p5870 (AtGA5) |  |  | This study |  |
| pCfB5871 | p5871 (AtGA3OX1) |  |  | This study |  |
| pCfB5872 | p5872 (GfCPR) |  |  | This study |  |
| pCfB5873 | p5873 (GfDES) |  |  | This study |  |
| pCfB6778 | p6778 (SsGGPS7) |  |  | This study |  |
| pCfB6781 | p6781 (GfCyb5) |  |  | This study |  |
| pCfB6782 | p6782 (GfP450-3_UP) |  |  | This study |  |
| pCfB8066 | p8066 (GfCyb5Red) |  |  | This study |  |

**Supplementary Table S7. List of strains used in this study**

| **Strain ID** | **Genotypes** | **Parent strain** | **Plasmid/ Biobricks** | **Reference** |
| --- | --- | --- | --- | --- |
| ST3683 | mus51∆, nugm -Htg2, ndh 2i, lys11-, leu2-, ura3-, MatB |  |  | (Angerer *et al.*, 2014) |
| ST5197 | ↑YltHMG1-↑YlGGS1(Hph) | ST3683 | pCfB5122 | This study |
| **Plant pathway non-optimized** | | | | |
| ST5292 | ↑AtGA1-↑AtGA2(Nat) | ST3683 | pCfB5249 | This study |
| ST6624 | ↑AtGA1-↑AtGA2(Nat) ↑AtGA3(Hph) | ST5292 | pCfB6599 | This study |
| ST6625 | ↑AtGA1-↑AtGA2 ↑AtGA3 | ST6624 | pCfB4158 | This study |
| ST6687 | ↑AtGA1-↑AtGA2 ↑AtGA3 ↑YlCyb5-↑AtATR2(Nat) | ST6625 | pCfB6563 | This study |
| **Plant pathway precursor optimized** | | | | |
| ST5294 | ↑YltHMG1-↑YlGGS1(Hph) ↑AtGA1-↑AtGA2(Nat) | ST5197 | pCfB5249 | This study |
| ST5959 | ↑YltHMG1-↑GGS1 ↑AtGA1-↑AtGA2 | ST5294 | pCfB4158 | This study |
| ST6295 | ↑YltHMG1-↑YlGGS1 ↑AtGA1-↑AtGA2 PrSQS1_50bp:SQS1(URA3) | ST5959 | BB1967, BB1970 | This study |
| ST6344 | ↑YltHMG1-↑YlGGS1 ↑AtGA1-↑AtGA2 PrSQS1_50bp:SQS1(URA3) ↑AtGA3(Hph) | ST6295 | pCfB6599 | This study |
| ST6349 | ↑YltHMG1-↑YlGGS1 ↑AtGA1-↑AtGA2 PrSQS1_50bp:SQS1(URA3) ↑AtGA3(Hph) ↑YlCyb5-↑AtATR2(Nat) | ST6344 | pCfB6563 | This study |
| ST6440 | ↑YltHMG1-↑YlGGS1 ↑AtGA1-↑AtGA2 PrSQS1_50bp:SQS1 ↑AtGA3 ↑YlCyb5-↑AtATR2 | ST6349 | pCfB4158 | This study |
| ST6343 | ↑YltHMG1-↑YlGGS1 ↑AtGA1-↑AtGA2 PrSQS1_50bp:SQS1(URA3) ↑AtGA3-AtKAO2(Hph) | ST6259 | pCfB6598 | This study |
| ST6348 | ↑YltHMG1-↑YlGGS1 ↑AtGA1-↑AtGA2 PrSQS1_50bp:SQS1(URA3) ↑AtGA3-AtKAO2(Hph) ↑YlCyb5-↑AtATR2(Nat) | ST6343 | pCfB6563 | This study |
| ST6439 | ↑YltHMG1-↑YlGGS1 ↑AtGA1-↑AtGA2 PrSQS1_50bp:SQS1 ↑AtGA3-↑AtKAO2 ↑YlCyb5-↑AtATR2 | ST6348 | pCfB4158 | This study |
| ST6513 | ↑YltHMG1-↑YlGGS1 ↑AtGA1-↑AtGA2 PrSQS1_50bp:SQS1 ↑AtGA3-↑AtKAO2 ↑YlCyb5-↑AtATR2 ↑AtGA5-↑AtGA3ox(Nat) | ST6439 | pCfB6793 | This study |
| ST6514 | ↑YltHMG1-↑YlGGS1 ↑AtGA1-↑AtGA2 PrSQS1_50bp:SQS1 ↑AtGA3-↑AtKAO2 ↑YlCyb5-↑AtATR2 ↑AtGA5-↑AtGA3ox(Nat) ↑GfP450-3-↑GfDes(Hph) | ST6513 | pCfB6794 | This study |
| ST7827 | ↑YltHMG1-↑YlGGS1 ↑AtGA1-↑AtGA2 PrSQS1_50bp:SQS1 ↑AtGA3-↑AtKAO2 ↑YlCyb5-↑AtATR2 ↑AtGA5-↑AtGA3ox ↑GfP450-3-↑GfDes | ST6514 | pCfB4158 | This study |
| **Plant pathway with GGPPS, GfCyb5, GfCybRed, GfCPR** | | | | |
| ST8257 | ↑YltHMG1-↑YlGGS1 ↑AtGA1-↑AtGA2 PrSQS1_50bp:SQS1 ↑AtGA3-↑AtKAO2 ↑YlCyb5-↑AtATR2 ↑Cas9(Hph) | ST6439 | pCfB5921 | This study |
| ST8414 | ↑YltHMG1-↑YlGGS1 ↑AtGA1-↑AtGA2 PrSQS1_50bp:SQS1 ↑AtGA3-↑AtKAO2 ↑YlCyb5-↑AtATR2 ↑Cas9(Hph) ↑SsGGPPS7 | ST8257 | pCfB8125, pCfB6631 | This study |
| ST8416 | ↑YltHMG1-↑YlGGS1 ↑AtGA1-↑AtGA2 PrSQS1_50bp:SQS1 ↑AtGA3-↑AtKAO2 ↑YlCyb5-↑AtATR2 ↑Cas9(Hph) ↑SsGGPPS7 ↑GfCyb5-↑GfCybRed-↑GfCPR | ST8414 | pCfB8141, pCfB6637 | This study |
| ST8447 | ↑YltHMG1-↑YlGGS1 ↑AtGA1-↑AtGA2 PrSQS1_50bp:SQS1 ↑AtGA3-↑AtKAO2 ↑YlCyb5-↑AtATR2 ↑Cas9(Hph) ↑SsGGPPS7 ↑GfCyb5-↑GfCybRed-↑GfCPR ↑AtGA5-PExp-PTefintron-↑AtGA3ox | ST8416 | pCfB8246, pCfB6630 | This study |
| ST8448 | ↑YltHMG1-↑YlGGS1 ↑AtGA1-↑AtGA2 PrSQS1_50bp:SQS1 ↑AtGA3-↑AtKAO2 ↑YlCyb5-↑AtATR2 ↑Cas9(Hph) ↑SsGGPPS7 ↑GfCyb5-↑GfCybRed-↑GfCPR ↑AtGA5-PTefintron-PGPD-↑AtGA3ox | ST8416 | pCfB8247, pCfB6630 | This study |
| ST8504 | ↑YltHMG1-↑YlGGS1 ↑AtGA1-↑AtGA2 PrSQS1_50bp:SQS1 ↑AtGA3-↑AtKAO2 ↑YlCyb5-↑AtATR2 ↑Cas9(Hph) ↑SsGGPPS7 ↑GfCyb5-↑GfCybRed-↑GfCPR ↑AtGA5-PExp-PTefintron-↑AtGA3ox ↑GfP450-3-↑GfDes(Nat) | ST8447 | pCfB8299 | This study |
| ST8505 | ↑YltHMG1-↑YlGGS1 ↑AtGA1-↑AtGA2 PrSQS1_50bp:SQS1 ↑AtGA3-↑AtKAO2 ↑YlCyb5-↑AtATR2 ↑Cas9(Hph) ↑SsGGPPS7 ↑GfCyb5-↑GfCybRed-↑GfCPR ↑AtGA5-PTefintron-PGPD-↑AtGA3ox ↑GfP450-3-↑GfDes(Nat) | ST8448 | pCfB8299 | This study |
| **Fungal pathway non-optimized** | | | | |
| ST6560 | ↑GfCPS/KS(Nat) | ST3683 | pCfB6804 | This study |
| ST6567 | ↑GfCPS/KS(Nat) ↑GfP450-4(Hph) | ST6560 | pCfB6600 | This study |
| ST6568 | ↑GfCPS/KS ↑GfP450-4 | ST6567 | pCfB4158 | This study |
| ST6569 | ↑GfCPS/KS ↑GfP450-4 ↑GfCyb5-↑GfCPR(Nat) | ST6568 | pCfB6832 | This study |
| **Fungal pathway precursor optimized** | | | | |
| ST5960 | ↑YltHMG1-↑YlGGS1 | ST5197 | pCfB4158 | This study |
| ST6296 | ↑YltHMG1-↑YlGGS1 PrSQS1_50bp:SQS1(URA3) | ST5960 | BB1967, BB1970 | This study |
| ST6516 | ↑YltHMG1-↑YlGGS1 PrSQS1_50bp:SQS1(URA3) ↑GfCPS/KS(Nat) | ST6296 | pCfB6804 | This study |
| ST6517 | ↑YltHMG1-↑YlGGS1 PrSQS1_50bp:SQS1(URA3) ↑GfCPS/KS(Nat) ↑GfP450-4(Hph) | ST6516 | pCfB6600 | This study |
| ST6519 | ↑YltHMG1-↑YlGGS1 PrSQS1_50bp:SQS1 ↑GfCPS/KS ↑GfP450-4 | ST6517 | pCfB4158 | This study |
| ST6521 | ↑YltHMG1-↑YlGGS1 PrSQS1_50bp:SQS1(URA3) ↑GfCPS/KS ↑GfP450-4 ↑GfCyb5-↑GfCPR(Nat) | ST6519 | pCfB6832 | This study |
| ST6518 | ↑YltHMG1-↑YlGGS1 PrSQS1_50bp:SQS1(URA3) ↑GfCPS/KS(Nat) ↑GfP450-4-↑GfP450-1(Hph) | ST6516 | pCfB6596 | This study |
| ST6520 | ↑YltHMG1-↑YlGGS1 PrSQS1_50bp:SQS1 ↑GfCPS/KS ↑GfP450-4-↑GfP450-1 | ST6518 | pCfB4158 | This study |
| ST6522 | ↑YltHMG1-↑YlGGS1 PrSQS1_50bp:SQS1 ↑GfCPS/KS ↑GfP450-4-↑GfP450-1 ↑GfCyb5-↑GfCPR(Nat) | ST6520 | pCfB6832 | This study |
| ST6762 | ↑YltHMG1-↑YlGGS1 PrSQS1_50bp:SQS1 ↑GfCPS/KS ↑GfP450-4-↑GfP450-1 ↑GfCyb5-↑GfCPR | ST6522 | pCfB4158 | This study |
| ST6843 | ↑YltHMG1-↑YlGGS1 PrSQS1_50bp:SQS1 ↑GfCPS/KS ↑GfP450-4-↑GfP450-1 ↑GfCyb5-↑GfCPR ↑GfP450-2(Nat) | ST6762 | pCfB6792 | This study |
| ST6844 | ↑YltHMG1-↑YlGGS1 PrSQS1_50bp:SQS1 ↑GfCPS/KS ↑GfP450-4-↑GfP450-1 ↑GfCyb5-↑GfCPR ↑GfP450-2(Nat) ↑GfP450-3-↑GfDes(Hph) | ST6843 | pCfB6794 | This study |
| ST7828 | ↑YltHMG1-↑YlGGS1 PrSQS1_50bp:SQS1 ↑GfCPS/KS ↑GfP450-4-↑GfP450-1 ↑GfCyb5-↑GfCPR ↑GfP450-2 ↑GfP450-3-↑GfDes | ST6844 | pCfB4158 | This study |
| **Combined Plant and fungal pathways with GGPPS** | | | | |
| ST8258 | ↑YltHMG1-↑YlGGS1 ↑AtGA1-↑AtGA2 PrSQS1_50bp:SQS1 ↑AtGA3 ↑YlCyb5-↑AtATR2 ↑Cas9(Hph) | ST6440 | pCfB5921 | This study |
| ST8415 | ↑YltHMG1-↑YlGGS1 ↑AtGA1-↑AtGA2 PrSQS1_50bp:SQS1 ↑AtGA3 ↑YlCyb5-↑AtATR2 ↑Cas9(Hph) ↑SsGGPPS7 | ST8258 | pCfB8125, pCfB6631 | This study |
| ST8417 | ↑YltHMG1-↑YlGGS1 ↑AtGA1-↑AtGA2 PrSQS1_50bp:SQS1 ↑AtGA3 ↑YlCyb5-↑AtATR2 ↑Cas9(Hph) ↑SsGGPPS7 ↑GfCyb5-↑GfCybRed-↑GfCPR | ST8514 | pCfB8141, pCfB6637 | This study |
| ST8602 | ↑YltHMG1-↑YlGGS1 ↑AtGA1-↑AtGA2 PrSQS1_50bp:SQS1 ↑AtGA3 ↑YlCyb5-↑AtATR2 ↑Cas9(Hph) ↑SsGGPPS7 ↑GfCyb5-↑GfCybRed-↑GfCPR | ST8515 | pCfB8246, pCfB6630 | This study |
| ST8535 | ↑YltHMG1-↑YlGGS1 ↑AtGA1-↑AtGA2 PrSQS1_50bp:SQS1 ↑AtGA3 ↑YlCyb5-↑AtATR2 ↑Cas9(Hph) ↑SsGGPPS7 ↑GfCyb5-↑GfCybRed-↑GfCPR ↑GfP450-3-↑GfDes(Nat) | ST8417 | pCfB8299 | This study |
| ST8580 | ↑YltHMG1-↑YlGGS1 ↑AtGA1-↑AtGA2 PrSQS1_50bp:SQS1 ↑AtGA3 ↑YlCyb5-↑AtATR2 ↑Cas9(Hph) ↑SsGGPPS7 ↑GfCyb5-↑GfCybRed-↑GfCPR ↑GfP450-3-↑GfDes(Nat) ↑GfP450-2-↑GfP450-1 | ST8535 | pCfB8398, pCfB8399 | This study |
| **Strains with improved KA/GA-production** | | | | |
| ST10331 | ↑YltHMG1-↑YlGGS1 ↑AtGA1-↑AtGA2 PrSQS1_50bp:SQS1 ↑AtGA3 ↑YlCyb5-↑AtATR2 ↑Cas9(Hph) ↑SsGGPPS7 ↑GfCyb5-↑GfCybRed-↑GfCPR ↑GfP450-3-↑GfDes(Nat) ↑GfP450-2-↑GfP450-1 ↑AtKO ↑AtCPS-link-AtKS | ST8580 | pCfB10128 (pIntA-1-URA-TPex20-AtKO<-PrGPD-Tefintron->AtCPS-link-AtKS-TLip2_v2) | This study |
| ST10332 | ↑YltHMG1-↑YlGGS1 ↑AtGA1-↑AtGA2 PrSQS1_50bp:SQS1 ↑AtGA3 ↑YlCyb5-↑AtATR2 ↑Cas9(Hph) ↑SsGGPPS7 ↑GfCyb5-↑GfCybRed-↑GfCPR ↑GfP450-3-↑GfDes(Nat) ↑GfP450-2-↑GfP450-1 ↑AtKS ↑AtCPS ↑AtKO | ST8580 | pCfB10129 (pIntA-1-URA-TPex20-AtKS<-PrGPD-Tefintron->AtCPS-Tpot1-PrEXP->AtKO-TLip2_v2) | This study |
| ST10333 | ↑YltHMG1-↑YlGGS1 ↑AtGA1-↑AtGA2 PrSQS1_50bp:SQS1 ↑AtGA3 ↑YlCyb5-↑AtATR2 ↑Cas9(Hph) ↑SsGGPPS7 ↑GfCyb5-↑GfCybRed-↑GfCPR ↑GfP450-3-↑GfDes(Nat) ↑GfP450-2-↑GfP450-1 ↑GfKO ↑GfCPS/KS | ST8580 | pCfB10130 (pIntA-1-URA-TPex20-GfKO<-PrGPD-Tefintron->GfCPS/KS-TLip2_v2) | This study |
| ST10334 | ↑YltHMG1-↑YlGGS1 ↑AtGA1-↑AtGA2 PrSQS1_50bp:SQS1 ↑AtGA3 ↑YlCyb5-↑AtATR2 ↑Cas9(Hph) ↑SsGGPPS7 ↑GfCyb5-↑GfCybRed-↑GfCPR ↑GfP450-3-↑GfDes(Nat) ↑GfP450-2-↑GfP450-1 ↑tAtKO ↑tAtCPS-link-tAtKS | ST8580 | pCfB10131 (pIntA-1-URA-TPex20-tAtKO<-PrGPD-Tefintron->tAtCPS-link-tAtKS-TLip2_v2) | This study |
| ST10350 | ↑YltHMG1-↑YlGGS1 ↑AtGA1-↑AtGA2 PrSQS1_50bp:SQS1 ↑AtGA3 ↑YlCyb5-↑AtATR2 ↑Cas9(Hph) ↑SsGGPPS7 ↑GfCyb5-↑GfCybRed-↑GfCPR ↑GfP450-3-↑GfDes(Nat) ↑GfP450-2-↑GfP450-1 ↑tAtKS ↑tAtCPS ↑tAtKO | ST8580 | pCfB10135 (pIntA-1-URA-TPex20-tAtKS<-PrGPD-Tefintron->tAtCPS-Tpot1-PrEXP->tAtKO-TLip2_v2.2) | This study |

**Supplementary Sequences**

**Synthetic gene sequences**

*AtCPS (AtGA2)* gene from *A. thaliana*

ATGTCTCTGCAGTACCACGTGCTGAACTCTATTCCCTCTACCACCTTCCTGTCCTCTACCAAGACTACTATCTCTTCTTCTTTCCTGACCATCTCTGGCTCCCCCCTGAACGTGGCCCGAGACAAGTCTCGATCTGGCTCTATCCACTGCTCTAAGCTGCGAACCCAAGAGTACATCAACTCTCAAGAGGTGCAGCACGACCTGCCCCTGATCCACGAGTGGCAGCAGCTGCAGGGCGAGGACGCCCCCCAGATCTCTGTGGGCTCTAACTCTAACGCCTTCAAGGAAGCCGTGAAGTCTGTCAAGACCATCCTGCGAAACCTGACCGACGGCGAGATCACCATCTCTGCCTACGACACCGCCTGGGTGGCCCTGATCGACGCTGGCGACAAGACCCCCGCCTTCCCATCTGCCGTGAAGTGGATCGCCGAGAACCAGCTGTCTGACGGCTCTTGGGGCGACGCCTACCTGTTCTCTTACCACGACCGACTGATCAACACCCTGGCCTGCGTGGTGGCCCTGCGATCTTGGAACCTGTTCCCCCACCAGTGCAACAAGGGCATCACATTCTTCCGAGAGAACATCGGCAAGCTCGAGGATGAGAACGACGAGCACATGCCCATCGGCTTCGAGGTGGCCTTCCCCTCTCTGCTGGAAATCGCCCGAGGCATCAACATCGACGTGCCCTACGACTCTCCCGTGCTGAAGGACATCTACGCCAAGAAGGAACTGAAGCTGACCCGAATCCCCAAGGAAATCATGCACAAGATCCCTACCACCCTGCTGCACTCGCTCGAGGGCATGCGAGATCTGGACTGGGAGAAGCTGCTGAAGCTGCAGTCTCAGGACGGATCTTTCCTGTTCTCTCCCTCGTCTACCGCCTTCGCCTTCATGCAGACCCGAGACTCTAACTGCCTCGAGTACCTGCGAAACGCTGTGAAGCGATTCAACGGCGGCGTGCCCAACGTGTTCCCCGTGGACCTGTTCGAGCACATCTGGATCGTGGACCGACTGCAGCGACTGGGCATCTCTCGATACTTCGAGGAAGAGATCAAGGAATGCCTGGACTACGTGCACCGATACTGGACCGACAACGGCATCTGCTGGGCCCGATGCTCTCACGTGCAGGACATCGACGACACCGCCATGGCCTTCCGACTGCTGCGACAGCACGGCTACCAGGTGTCTGCCGACGTGTTCAAGAACTTCGAGAAGGAAGGCGAGTTCTTCTGCTTCGTGGGCCAGTCTAACCAGGCCGTGACCGGCATGTTCAACCTGTACCGAGCCTCTCAGCTGGCCTTCCCCAGAGAGGAAATCCTGAAGAACGCCAAGGAATTCTCTTACAACTACCTGCTCGAGAAGCGAGAGCGAGAGGAACTGATCGACAAGTGGATCATCATGAAGGACCTGCCCGGCGAGATCGGCTTCGCCCTCGAGATCCCCTGGTACGCCTCTCTGCCCCGAGTCGAGACTCGATTCTACATCGACCAGTACGGCGGCGAGAACGACGTGTGGATCGGCAAGACCCTGTACCGAATGCCCTACGTGAACAACAACGGCTACCTCGAGCTGGCCAAGCAGGACTACAACAACTGCCAGGCCCAGCACCAGCTCGAGTGGGACATCTTCCAGAAGTGGTACGAAGAGAACCGACTGTCTGAGTGGGGCGTGCGACGATCTGAGCTGCTCGAGTGCTACTACCTGGCCGCTGCCACCATCTTCGAGTCTGAGCGATCTCACGAGCGAATGGTGTGGGCCAAGTCCTCTGTGCTGGTGAAGGCCATCTCTTCTAGCTTCGGCGAGTCCTCTGACTCTCGACGATCTTTCTCGGACCAGTTCCACGAGTACATTGCCAACGCCCGACGATCTGACCACCACTTCAACGACCGAAACATGCGACTGGACCGACCCGGCTCTGTGCAGGCCTCTCGACTGGCTGGCGTGCTGATCGGCACCCTGAACCAGATGTCTTTCGACCTGTTCATGTCTCACGGCCGAGATGTGAACAACCTGCTGTACCTGTCTTGGGGAGACTGGATGGAAAAGTGGAAGCTGTACGGCGACGAGGGCGAGGGCGAGCTGATGGTGAAGATGATCATCCTGATGAAGAACAACGACCTGACCAACTTCTTCACCCACACCCACTTCGTGCGACTGGCCGAGATCATCAACCGAATCTGCCTGCCCCGACAGTACCTGAAGGCCCGACGAAACGACGAGAAGGAAAAGACCATCAAGTCTATGGAAAAGGAAATGGGCAAGATGGTCGAGCTGGCCCTGTCTGAGTCTGACACCTTCCGAGATGTGTCTATCACCTTCCTGGACGTGGCCAAGGCCTTCTACTACTTCGCCCTGTGCGGCGACCACCTCCAGACCCACATCTCTAAGGTGCTGTTCCAGAAGGTGTAA

*AtKS (AtGA2)* gene from *A. thaliana*

ATGTCGATCAACCTGCGATCTTCTGGCTGTTCTTCTCCCATCTCTGCCACCCTCGAGCGAGGCCTGGACTCTGAGGTGCAGACCCGAGCCAACAACGTGTCTTTCGAGCAGACCAAGGAAAAGATCCGAAAGATGCTCGAGAAGGTCGAGCTGTCTGTGTCTGCCTACGACACCTCTTGGGTGGCCATGGTGCCCTCGCCCTCTTCGCAGAACGCCCCCCTGTTCCCCCAGTGCGTGAAGTGGCTGCTGGACAACCAGCACGAGGACGGCTCTTGGGGCCTGGACAACCACGACCACCAGTCTCTGAAGAAGGACGTCCTGTCCTCTACCCTGGCCTCTATCCTGGCCCTGAAGAAGTGGGGCATCGGCGAGCGACAGATCAACAAGGGCCTGCAGTTCATCGAGCTGAACTCTGCCCTGGTGACCGACGAGACTATCCAGAAGCCCACCGGCTTCGACATCATCTTCCCCGGCATGATCAAGTACGCCCGAGATCTGAACCTGACCATCCCCCTGGGATCTGAGGTGGTGGACGACATGATCCGAAAGCGAGATCTGGACCTCAAGTGCGACTCTGAGAAGTTCTCTAAGGGCCGAGAGGCCTACCTGGCCTACGTGCTCGAGGGCACCCGAAACCTGAAGGACTGGGACCTGATCGTGAAGTACCAGCGAAAGAACGGCTCTCTGTTCGACTCTCCCGCCACCACCGCCGCTGCCTTCACCCAGTTCGGCAACGACGGCTGCCTGCGATACCTGTGCTCTCTGCTGCAAAAGTTCGAGGCCGCCGTGCCCTCTGTGTACCCCTTCGACCAGTACGCTCGACTGTCTATCATCGTGACCCTCGAGTCTCTGGGCATCGACCGAGACTTCAAGACCGAGATCAAGTCTATCCTGGACGAGACTTACCGATACTGGCTGCGAGGCGACGAGGAAATCTGCCTGGACCTGGCCACCTGTGCCCTGGCCTTCCGACTGCTGCTGGCCCACGGCTACGACGTGTCTTACGACCCCCTGAAGCCCTTCGCCGAGGAATCTGGCTTCTCCGACACTCTCGAGGGCTACGTGAAGAACACCTTCTCTGTGCTCGAGCTGTTCAAGGCCGCCCAGTCTTACCCCCACGAGTCTGCTCTGAAGAAGCAGTGCTGCTGGACCAAGCAGTACCTCGAGATGGAACTGTCCTCTTGGGTCAAGACCTCTGTGCGAGACAAGTACCTGAAGAAGGAAGTCGAGGACGCCCTGGCTTTCCCCTCTTACGCCTCTCTCGAGCGATCTGACCACCGACGAAAGATCCTGAACGGCTCTGCCGTCGAGAACACCCGAGTGACCAAGACCTCTTACCGACTGCACAACATCTGCACCTCTGACATCCTGAAGCTGGCCGTGGACGACTTCAACTTCTGCCAGTCTATCCACCGAGAGGAAATGGAACGACTGGACCGATGGATCGTCGAGAACCGACTGCAAGAGCTGAAGTTCGCCCGACAGAAGCTGGCCTACTGCTACTTCTCTGGCGCCGCTACCCTGTTCTCGCCCGAGCTGTCTGACGCCCGAATCTCTTGGGCCAAGGGCGGCGTGCTGACTACCGTGGTCGACGACTTCTTCGACGTGGGCGGCTCTAAGGAAGAACTCGAGAACCTGATCCACCTGGTCGAGAAGTGGGACCTGAACGGCGTGCCCGAGTACTCTTCTGAGCACGTCGAGATCATCTTCTCCGTGCTGCGAGACACCATCCTCGAGACTGGCGACAAGGCCTTCACCTACCAGGGCCGAAACGTGACCCACCACATCGTGAAGATCTGGCTGGACCTGCTGAAGTCTATGCTGCGAGAGGCCGAGTGGTCCTCTGACAAGTCTACCCCTTCGCTCGAGGACTACATGGAAAACGCCTACATCTCTTTCGCCCTGGGCCCCATCGTGCTGCCCGCCACCTACCTGATCGGACCCCCCCTGCCCGAAAAGACCGTGGACTCTCACCAGTACAACCAGCTGTACAAGCTCGTGTCTACCATGGGCCGACTGCTGAACGACATCCAGGGCTTCAAGCGAGAGTCTGCCGAGGGCAAGCTGAACGCCGTGTCTCTGCACATGAAGCACGAGCGAGACAACCGATCTAAGGAAGTGATCATCGAGTCTATGAAGGGCCTGGCCGAGCGAAAGCGAGAGGAACTGCACAAGCTGGTGCTGGAAGAGAAGGGCTCTGTGGTGCCCCGAGAGTGCAAGGAAGCCTTCCTGAAGATGTCTAAGGTGCTGAACCTGTTCTACCGAAAGGACGACGGCTTCACCTCTAACGACCTGATGTCTCTGGTGAAGTCTGTGATCTACGAGCCCGTGTCCCTGCAGAAGGAATCTCTGACCTAA

*AtKO (AtGA3)* gene from *A. thaliana*

ATGGCCTTCTTCTCCATGATCTCTATCCTGCTGGGCTTCGTGATCTCTTCGTTCATCTTCATCTTTTTCTTCAAAAAACTGCTGTCTTTCTCGCGAAAGAACATGTCTGAGGTGTCTACCCTGCCCTCTGTGCCCGTGGTGCCCGGCTTCCCCGTGATCGGCAACCTGCTGCAGCTGAAGGAAAAGAAGCCCCACAAGACCTTCACCCGATGGTCTGAGATCTACGGCCCCATCTACTCTATCAAGATGGGATCTTCTTCTCTGATCGTGCTGAACTCTACCGAGACTGCCAAGGAAGCCATGGTCACCCGATTCTCTTCTATCTCTACCCGAAAGCTGTCTAACGCCCTGACCGTGCTGACCTGCGACAAGTCTATGGTGGCTACCTCTGACTACGACGACTTCCACAAGCTGGTGAAGAGATGCCTGCTGAACGGCCTGCTGGGCGCCAACGCCCAGAAGCGAAAGCGACACTACCGAGATGCCCTGATCGAGAACGTGTCCTCTAAGCTGCACGCCCACGCCCGAGATCACCCCCAAGAGCCCGTGAACTTCCGAGCCATCTTCGAGCACGAGCTGTTCGGCGTGGCCCTGAAGCAGGCCTTCGGCAAGGACGTCGAGTCTATCTACGTGAAGGAACTGGGCGTGACCCTGTCTAAGGACGAGATCTTCAAGGTGCTGGTGCACGACATGATGGAAGGCGCCATCGACGTGGACTGGCGAGACTTCTTCCCATACCTGAAATGGATCCCCAACAAGTCTTTCGAGGCCCGAATCCAGCAGAAGCACAAGCGACGACTGGCCGTGATGAACGCCCTGATCCAGGACCGACTGAAGCAGAACGGCTCTGAGTCTGACGACGACTGCTACCTGAACTTCCTGATGTCTGAGGCCAAGACCCTGACCAAGGAACAGATCGCCATCCTGGTGTGGGAGACTATCATCGAGACTGCCGACACCACCCTGGTGACCACCGAGTGGGCCATCTACGAGCTGGCCAAGCACCCCTCTGTGCAGGACCGACTCTGCAAGGAAATCCAGAACGTCTGCGGCGGAGAGAAGTTCAAGGAAGAACAGCTGTCTCAGGTGCCCTACCTGAACGGCGTGTTCCACGAGACTCTGCGAAAGTACTCTCCCGCTCCCCTGGTGCCCATCCGATACGCCCACGAGGACACCCAGATCGGCGGCTACCACGTGCCCGCTGGCTCTGAGATCGCCATCAACATCTACGGCTGCAACATGGACAAGAAGAGATGGGAGCGACCCGAGGACTGGTGGCCCGAGCGATTCCTGGACGACGGCAAGTACGAGACTTCTGACCTGCACAAGACCATGGCCTTCGGAGCCGGCAAGCGAGTGTGCGCTGGCGCCCTGCAGGCTTCTCTGATGGCCGGAATCGCCATCGGCCGACTGGTGCAAGAGTTCGAGTGGAAGCTGCGAGATGGCGAGGAAGAGAACGTCGACACCTACGGCCTGACCTCTCAAAAGCTGTACCCCCTGATGGCCATTATCAACCCCCGACGATCTTAA

*AtKAO2* gene from *A. thaliana*

ATGACCGAGACTGGCCTGATCCTGATGTGGTTCCCCCTGATCATCCTGGGCCTGTTCGTGCTGAAGTGGGTGCTGAAGCGAGTGAACGTGTGGATCTACGTGTCTAAGCTGGGCGAGAAGAAGCACTACCTGCCCCCTGGCGACCTGGGCTGGCCCGTGATCGGCAACATGTGGTCTTTTCTGCGAGCCTTCAAGACCTCTGACCCCGAGTCTTTCATCCAGTCTTACATCACCCGATACGGCCGAACCGGCATCTACAAGGCCCACATGTTCGGCTACCCCTGCGTGCTGGTGACCACCCCCGAGACTTGCCGACGAGTGCTGACCGACGACGACGCCTTCCACATCGGCTGGCCCAAGTCTACCATGAAGCTGATCGGCCGAAAGTCTTTCGTGGGCATCTCTTTCGAGGAGCACAAGCGACTGCGACGACTGACCTCTGCCCCCGTGAACGGCCCCGAGGCCCTGTCTGTGTACATCCAGTTCATCGAGGAGACTGTGAACACCGACCTCGAGAAGTGGTCTAAGATGGGAGAGATCGAGTTCCTGTCTCACCTCCGAAAGCTGACCTTCAAGGTGATTATGTACATCTTCCTGTCCTCTGAGTCTGAGCACGTGATGGACTCCCTCGAGCGAGAGTACACCAACCTGAACTACGGCGTGCGAGCCATGGGCATCAACCTGCCCGGCTTCGCCTACCACCGAGCCCTGAAGGCCCGAAAGAAGCTGGTCGCCGCCTTCCAGTCTATCGTGACCAACCGACGAAACCAGCGAAAGCAGAACATCTCTTCTAACCGAAAGGACATGCTGGACAACCTGATCGACGTGAAGGACGAGAACGGCCGAGTGCTGGACGACGAGGAGATCATCGACCTGCTGCTGATGTACCTGAACGCCGGCCACGAGTCCTCTGGCCACCTGACCATGTGGGCCACCATCCTGATGCAGGAGCACCCCATGATCCTGCAGAAGGCCAAGGAGGAGCAGGAGCGAATCGTCAAGAAGCGAGCCCCCGGACAGAAGCTGACCCTGAAGGAGACTCGAGAGATGGTGTACCTGTCTCAGGTGATCGACGAGACTCTGCGAGTGATCACCTTCTCCCTGACCGCCTTCCGAGAGGCCAAGTCTGACGTGCAGATGGACGGCTACATCATCCCCAAGGGCTGGAAGGTGCTGACCTGGTTCCGAAACGTGCACCTGGACCCCGAGATCTACCCCGACCCCAAGAAGTTCGACCCCTCTCGATGGGAGGGCTACACCCCCAAGGCCGGCACCTTCCTGCCTTTCGGCCTGGGCTCTCACCTGTGCCCCGGCAACGACCTGGCCAAGCTCGAGATCTCTATCTTCCTGCACCACTTTCTGCTGAAGTACCGAGTCGAGCGATCTAACCCCGGCTGCCCCGTGATGTTCCTGCCCCACAACCGACCCAAGGACAACTGCCTGGCCCGAATCACCCGAACCATGCCCTGA

*AtC20ox (AtGA20ox1)* gene from *A. thaliana*

ATGGCCGTGTCTTTCGTGACCACCTCTCCTGAGGAAGAGGACAAGCCCAAGCTCGGCCTGGGCAACATTCAGACCCCTCTGATCTTCAACCCCTCTATGCTGAACCTGCAGGCCAACATTCCCAACCAGTTCATCTGGCCCGACGACGAGAAGCCCTCTATCAACGTGCTCGAGCTGGACGTGCCCCTGATCGACCTGCAGAACCTGCTGTCTGACCCCTCTTCTACCCTGGACGCCTCTCGACTGATCTCTGAGGCCTGCAAGAAGCACGGATTCTTTCTGGTGGTGAACCACGGCATCTCCGAGGAACTGATTTCTGACGCCCACGAGTACACCTCTCGATTCTTCGACATGCCCCTGTCTGAGAAGCAGCGAGTGCTGCGAAAGTCTGGCGAGTCTGTGGGCTACGCCTCTTCTTTCACCGGCCGATTCTCTACCAAGCTGCCCTGGAAGGAAACCCTGTCTTTCCGATTCTGCGACGACATGTCTCGATCTAAGTCTGTGCAGGACTACTTCTGTGACGCCCTCGGCCACGGCTTCCAGCCTTTCGGCAAGGTGTACCAAGAGTACTGCGAGGCTATGTCCTCTCTGTCTCTGAAGATCATGGAACTGCTGGGCCTGTCTCTGGGCGTGAAGCGAGACTACTTCCGAGAGTTCTTTGAAGAGAACGACTCTATCATGCGACTGAACTACTACCCTCCTTGCATGAAGCCCGACCTGACTCTCGGCACCGGACCTCACTGCGACCCCACCTCGCTGACCATCCTGCACCAGGACCACGTGAACGGCCTCCAGGTGTTCGTCGAGAACCAGTGGCGATCTATTCGACCCAACCCTAAGGCCTTCGTGGTGAACATCGGCGACACCTTCATGGCCCTGTCTAACGACCGATACAAGTCTTGCCTGCACCGAGCCGTGGTCAACTCTAAGTCCGAGCGAAAGTCCCTGGCATTCTTCCTGTGTCCTAAGAAGGACCGAGTCGTCACCCCTCCACGAGAGCTGCTGGACTCTATTACCTCTCGACGATACCCCGACTTTACCTGGTCTATGTTCCTCGAGTTCACCCAGAAGCACTACCGAGCCGACATGAACACCCTGCAGGCTTTCTCTGACTGGCTGACCAAGCCTATCTAA

*AtC3ox (AtGA3ox1)* gene from *A. thaliana*

ATGCCCGCCATGCTGACCGACGTGTTCCGAGGACACCCCATTCATCTGCCCCACTCTCATATCCCCGACTTCACCTCTCTGCGAGAGCTGCCCGACTCTTACAAGTGGACCCCTAAGGACGACCTGCTGTTCTCTGCTGCTCCCTCGCCTCCTGCCACCGGCGAGAACATCCCTCTGATCGACCTGGATCACCCCGACGCCACCAACCAGATCGGCCACGCCTGCCGAACCTGGGGCGCCTTCCAGATCTCTAACCACGGCGTGCCCCTGGGCCTGCTGCAGGACATCGAGTTTCTGACCGGCTCTCTGTTCGGACTGCCCGTGCAGCGAAAGCTGAAGTCTGCCCGATCTGAGACTGGCGTGTCTGGCTACGGCGTGGCCCGAATCGCCTCGTTCTTCAACAAGCAGATGTGGTCTGAGGGCTTTACCATCACCGGCTCGCCCCTGAACGACTTCCGAAAGCTGTGGCCCCAGCACCACCTGAACTACTGCGACATCGTGGAAGAGTACGAGGAACACATGAAGAAGCTGGCCTCTAAGCTGATGTGGCTGGCCCTGAACTCTCTGGGAGTGTCTGAAGAGGACATTGAGTGGGCTTCTCTGTCCTCTGACCTGAACTGGGCCCAAGCCGCTCTGCAGCTGAACCACTATCCTGTGTGCCCCGAGCCTGACCGAGCCATGGGCCTCGCCGCTCACACCGACTCTACCCTGCTGACCATCCTGTACCAGAACAACACCGCCGGACTCCAGGTGTTTCGAGATGACCTCGGCTGGGTGACCGTGCCTCCTTTTCCTGGCTCTCTGGTGGTGAACGTGGGCGACCTGTTCCACATCCTGTCTAACGGCCTGTTCAAGTCTGTGCTGCATCGAGCCCGAGTGAACCAGACACGAGCCCGACTGTCTGTGGCCTTCCTGTGGGGACCCCAGTCTGACATCAAGATCTCTCCCGTGCCTAAGCTGGTGTCTCCCGTCGAGTCTCCTCTGTACCAGTCTGTGACCTGGAAGGAATACCTGCGAACTAAGGCTACCCACTTTAACAAGGCCCTGTCTATGATCCGAAACCACCGAGAGGAATAA

*AtATR2* gene from *A. thaliana*

ATGTCCTCTTCGTCGTCCTCTTCTACCTCTATGATCGACCTGATGGCCGCCATCATCAAGGGCGAGCCCGTGATCGTGTCTGACCCCGCCAACGCCTCTGCCTACGAGTCTGTGGCCGCCGAGCTGTCCTCTATGCTGATCGAGAACCGACAGTTCGCCATGATCGTGACCACCTCTATCGCCGTGCTGATCGGCTGCATCGTGATGCTGGTGTGGCGACGATCTGGCTCTGGCAACTCTAAGCGAGTCGAGCCCCTGAAGCCCCTGGTGATCAAGCCCCGAGAGGAGGAGATCGACGACGGCCGAAAGAAGGTGACCATCTTCTTCGGCACCCAGACCGGCACTGCCGAGGGCTTCGCCAAGGCCCTGGGCGAGGAGGCCAAGGCCCGATACGAGAAGACCCGATTCAAGATCGTGGACCTGGACGACTACGCCGCCGACGACGACGAGTACGAGGAGAAGCTGAAGAAGGAGGACGTCGCCTTCTTCTTCCTGGCCACCTACGGCGACGGCGAGCCCACCGACAACGCCGCTCGATTCTACAAGTGGTTCACCGAGGGCAACGACCGAGGCGAGTGGCTGAAGAACCTGAAGTACGGCGTGTTCGGCCTGGGCAACCGACAGTACGAGCACTTCAACAAGGTGGCCAAGGTGGTGGACGACATCCTGGTCGAGCAGGGCGCTCAGCGACTGGTGCAGGTCGGCCTGGGCGACGACGACCAGTGCATCGAGGACGACTTCACCGCCTGGCGAGAGGCCCTGTGGCCCGAGCTGGACACCATCCTGCGAGAGGAGGGCGACACCGCCGTCGCCACCCCTTACACCGCCGCTGTGCTCGAGTACCGAGTGTCTATCCACGACTCTGAGGACGCCAAGTTCAACGACATCAACATGGCCAACGGCAACGGCTACACCGTGTTCGACGCCCAGCACCCCTACAAGGCCAACGTGGCCGTGAAGCGAGAGCTGCACACCCCCGAGTCTGACCGATCTTGCATCCACCTCGAGTTCGACATTGCCGGCTCTGGCCTGACCTACGAGACTGGCGACCACGTGGGCGTGCTGTGCGACAACCTGTCTGAGACTGTGGACGAGGCCCTGCGACTGCTGGACATGTCTCCCGACACCTACTTCTCGCTGCACGCCGAGAAGGAGGATGGAACCCCCATCTCTTCGTCGCTGCCCCCTCCCTTCCCCCCCTGCAACCTGCGAACCGCCCTGACCCGATACGCCTGCCTGCTGTCCTCGCCCAAGAAGTCTGCCCTGGTCGCCCTGGCCGCTCACGCTTCTGACCCCACCGAGGCCGAGCGACTGAAGCACCTGGCCTCTCCCGCCGGAAAGGACGAGTACTCTAAGTGGGTGGTCGAGTCTCAGCGATCTCTGCTCGAGGTGATGGCCGAGTTCCCCTCGGCCAAGCCCCCTCTGGGCGTGTTCTTCGCCGGCGTGGCTCCCCGACTGCAGCCCCGATTCTACTCTATCTCTTCGTCCCCCAAGATTGCCGAGACTCGAATCCACGTGACCTGCGCCCTGGTGTACGAGAAGATGCCCACCGGCCGAATCCACAAGGGCGTGTGCTCTACCTGGATGAAGAACGCCGTGCCCTACGAGAAGTCTGAGAACTGTTCTTCTGCCCCCATCTTCGTGCGACAGTCTAACTTCAAGCTGCCCTCTGACTCTAAGGTGCCCATCATCATGATCGGACCCGGCACCGGCCTGGCTCCCTTCAGAGGCTTCCTGCAGGAGCGACTGGCCCTGGTCGAGTCTGGCGTCGAGCTGGGCCCCTCTGTGCTGTTCTTCGGCTGCCGAAACCGACGAATGGACTTCATCTACGAGGAGGAGCTGCAGCGATTCGTCGAGTCTGGTGCCCTGGCTGAGCTGTCTGTGGCCTTCTCGCGAGAGGGACCCACCAAGGAGTACGTCCAGCACAAGATGATGGACAAGGCCTCTGACATCTGGAACATGATCTCTCAGGGCGCCTACCTGTACGTGTGCGGCGACGCCAAGGGCATGGCCCGAGATGTGCACCGATCTCTGCACACCATTGCCCAGGAGCAGGGCTCTATGGACTCTACCAAGGCCGAGGGATTCGTGAAGAACCTGCAGACCTCTGGCCGATACCTGCGAGATGTGTGGTGA

*GfCPS/KS* gene from *G. fujikuroi*

ATGCCCGGCAAGATCGAGAACGGCACCCCCAAGGACCTCAAGACCGGCAACGACTTCGTGTCTGCCGCCAAGTCTCTGCTGGACCGAGCCTTCAAGTCTCACCACTCTTACTACGGCCTGTGCTCTACCTCTTGCCAGGTGTACGACACCGCCTGGGTCGCCATGATCCCCAAGACCCGAGACAACGTGAAGCAGTGGCTGTTCCCCGAGTGCTTCCACTACCTGCTCAAGACCCAGGCCGCCGACGGCTCTTGGGGCTCTCTGCCCACCACCCAGACCGCCGGCATCCTGGACACCGCCTCTGCCGTGCTGGCCCTGCTGTGCCACGCCCAAGAGCCCCTGCAGATCCTGGACGTGTCTCCCGACGAGATGGGCCTGCGAATCGAGCACGGCGTGACCTCCCTGAAGCGACAGCTGGCCGTGTGGAACGACGTCGAGGACACCAACCACATCGGCGTCGAGTTCATCATCCCCGCCCTGCTGTCTATGCTCGAGAAGGAACTGGACGTGCCCTCGTTCGAGTTCCCCTGCCGATCTATCCTCGAGCGAATGCACGGCGAGAAGCTGGGCCACTTCGACCTCGAGCAGGTCTACGGCAAGCCCTCTTCTCTGCTGCACTCTCTCGAGGCCTTTCTGGGCAAGCTGGACTTCGACCGACTGTCTCACCACCTGTACCACGGCTCTATGATGGCCTCTCCCTCTTCTACCGCCGCCTACCTGATCGGCGCCACCAAGTGGGACGACGAGGCCGAGGACTACCTGCGACACGTGATGCGAAACGGCGCTGGCCACGGCAACGGCGGCATCTCTGGCACCTTCCCCACCACCCACTTCGAGTGCTCTTGGATCATTGCCACCCTGCTGAAGGGCGGCTTCACCCTGAAGCAGATCGACGGCGACGGCCTGCGAGGCCTGTCTACCATCCTGCTTGAGGCCCTGCGAGATGAGAACGGCGTCATCGGCTTCGCTCCCCGAACCGCCGACGTGGACGATACCGCCAAGGCCCTGCTGGCCCTGTCTCTGGTGAACCAGCCCGTGTCCCCCGACATCATGATCAAGGGCTTCGAGGGCAAGGACCACTTCACCACCTTCGGCTCTGAGCGAGATCCCTCTCTGACCTCTAACCTGCACGTGCTGCTGTCTCTGCCCGGCAAGCAGTCTAACCTGTCTCAGTACCACCCCCAGATCCTCAAGACCACCCTGTTCACCTGTCGATGGTGGTGGGGCTCTGACCACTGCGTGAAGGACAAGTGGAACCTGTCTCACCTGTACCCCACCATGCTGCTGGTCGAGGCCTTCACCGAGGTGCTGCACCTGATCGACGGCGGCGAGCTGTCCTCTCTGTTCGACGAGTCTTTCAAGTGCAAGATCGGCCTGTCTATCTTCCAGGCCGTGCTGCGAATCATCCTGACCCAGGACAACGACGGATCTTGGCGAGGCTACCGAGAGCAGACCTGCTACGCCATCCTGGCCCTGGTGCAGGCCCGACACGTGTGTTTCTTCACCCACATGGTGGACCGACTGCAGTCTTGCGTGGACCGAGGATTCTCTTGGCTGAAGTCTTGCTCTTTCCACTCTCAGGACCTGACCTGGACCTCTAAGACCGCCTACGAGGTGGGCTTCGTGGCCGAGGCCTACAAGCTGGCCGCCCTGCAGTCTGCCTCCCTCGAGGTGCCCGCTGCCACCATCGGCCACTCTGTGACCTCCGCCGTGCCCTCTTCGGACCTCGAGAAGTACATGCGACTGGTGCGAAAGACCGCCCTGTTCTCGCCCCTGGACGAGTGGGGCCTGATGGCCTCTATTATCGAGTCCTCTTTCTTCGTGCCCCTGCTGCAGGCCCAGCGAGTCGAGATCTACCCCCGAGACAACATCAAGGTGGACGAGGACAAGTACCTGTCTATCATCCCCTTCACCTGGGTGGGCTGCAACAACCGATCTCGAACCTTCGCCTCTAACCGATGGCTGTACGACATGATGTACCTGTCTCTGCTGGGCTACCAGACCGACGAGTACATGGAAGCCGTGGCCGGACCCGTGTTCGGCGACGTGTCCCTGCTGCACCAGACCATTGACAAGGTGATCGACAACACCATGGGCAACCTGGCCCGAGCCAACGGCACCGTGCACTCTGGCAACGGCCACCAGCACGAGTCTCCCAACATCGGCCAGGTTGAGGACACTCTGACCCGATTCACCAACTCTGTGCTGAACCACAAGGACGTGCTGAACTCTTCGTCCTCTGACCAGGACACCCTGCGACGAGAGTTCCGAACCTTCATGCACGCCCACATCACCCAGATCGAGGACAACTCTCGATTCTCTAAGCAGGCCTCTTCTGACGCCTTCTCGTCCCCCGAGCAGTCTTACTTCCAGTGGGTGAACTCTACCGGCGGCTCTCACGTGGCCTGCGCCTACTCTTTCGCCTTCTCTAACTGCCTGATGTCTGCCAACCTGCTGCAGGGCAAGGACGCCTTCCCATCTGGCACCCAGAAGTACCTGATCTCTTCTGTGATGCGACACGCCACCAACATGTGCCGAATGTACAACGACTTCGGCTCTATCGCTCGAGACAACGCCGAGCGAAACGTGAACTCTATCCACTTCCCCGAGTTCACCCTGTGCAACGGCACCTCTCAGAACCTGGACGAGCGAAAGGAACGACTGCTGAAGATCGCCACCTACGAGCAGGGCTACCTCGACCGAGCCCTCGAGGCTCTCGAGCGACAGTCTCGAGATGACGCCGGCGACCGAGCTGGCTCTAAGGACATGCGAAAGCTGAAGATCGTGAAGCTGTTCTGCGACGTGACCGACCTGTACGACCAGCTGTACGTGATCAAGGACCTGTCCTCTTCGATGAAGTAA

*GfKO (GfP450-4)* gene from *G. fujikuroi*

ATGTCCAAGTCCAACTCCATGAACTCTACCTCTCACGAGACTCTGTTCCAGCAGCTGGTGCTGGGCCTGGACCGAATGCCCCTGATGGACGTGCACTGGCTGATCTACGTGGCCTTCGGCGCCTGGCTGTGCTCTTACGTGATCCACGTGCTGTCCTCTTCGTCTACCGTGAAGGTGCCCGTGGTGGGCTACCGATCTGTGTTCGAGCCCACCTGGCTGCTGCGACTGCGATTCGTGTGGGAGGGCGGCTCTATCATCGGCCAGGGCTACAACAAGTTCAAGGACTCTATCTTCCAGGTGCGAAAGCTGGGCACCGACATCGTGATCATCCCCCCCAACTACATCGACGAGGTCCGAAAGCTGTCTCAGGACAAGACCCGATCTGTCGAGCCCTTCATCAACGACTTCGCCGGCCAGTATACCCGAGGAATGGTGTTCCTGCAGTCTGACCTGCAGAACCGAGTGATCCAGCAGCGACTGACCCCCAAGCTGGTGTCTCTGACCAAGGTGATGAAGGAAGAACTGGACTACGCCCTGACCAAGGAAATGCCCGACATGAAGAACGACGAGTGGGTCGAGGTGGACATCTCTTCTATCATGGTGCGACTGATCTCTCGAATCTCTGCCCGAGTGTTCCTGGGCCCCGAGCACTGCCGAAACCAGGAATGGCTGACCACCACCGCCGAGTACTCTGAGTCTCTGTTCATCACCGGCTTCATCCTGCGAGTGGTGCCCCACATCCTGCGACCCTTCATTGCCCCCCTGCTGCCCTCTTACCGAACCCTGCTGCGAAACGTGTCCTCTGGCCGACGAGTGATCGGCGACATCATCCGATCTCAGCAGGGCGACGGCAACGAGGACATCCTGTCTTGGATGCGAGATGCCGCCACCGGCGAGGAAAAGCAGATCGACAACATTGCCCAGCGAATGCTGATCCTGTCTCTGGCCTCTATCCACACCACCGCCATGACCATGACCCACGCCATGTACGACCTGTGCGCCTGCCCCGAGTACATCGAGCCCCTGCGAGATGAGGTGAAGTCTGTGGTGGGCGCCTCTGGCTGGGACAAGACCGCCCTGAACCGATTCCACAAGCTGGACTCGTTTCTGAAGGAATCTCAGCGATTCAACCCCGTGTTCCTGCTGACCTTCAACCGAATCTACCACCAGTCTATGACCCTGTCTGACGGCACCAACATTCCCTCTGGCACCCGAATCGCCGTGCCCTCTCACGCCATGCTGCAGGACTCTGCCCACGTGCCCGGACCCACCCCCCCCACCGAGTTCGACGGCTTCCGATACTCTAAGATCCGATCTGACTCTAACTACGCCCAAAAGTACCTGTTCTCTATGACCGACTCTTCTAACATGGCCTTCGGCTACGGCAAGTACGCCTGTCCCGGCCGATTCTACGCCTCTAACGAGATGAAGCTGACCCTGGCCATCCTGCTGCTGCAGTTCGAGTTCAAGCTGCCCGACGGCAAGGGCCGACCCCGAAACATCACCATCGACTCTGACATGATCCCCGACCCCCGAGCCCGACTGTGCGTGCGAAAGCGATCTCTCCGAGATGAGTAA

*GfP450-1* gene from *G. fujikuroi*

ATGGCCAACCACTCTTCTTCGTACTACCACGAGTTCTACAAGGACCACTCTCACACCGTGCTGACCCTGATGTCTGAGAAGCCCGTGATCCTGCCCTCTCTGATCCTGGGCACCTGTGCCGTGCTGCTGTGCATCCAGTGGCTGAAGCCCCAGCCCCTGATCATGGTGAACGGCCGAAAGTTCGGCGAGCTGTCTAACGTGCGAGCCAAGCGAGACTTCACCTTCGGAGCCCGACAGCTGCTCGAGAAGGGCCTGAAGATGTCTCCCGACAAGCCCTTCCGAATCATGGGCGACGTGGGCGAGCTGCACATCCTGCCCCCCAAGTACGCCTACGAGGTGCGAAACAACGAGAAGCTGTCTTTCACCATGGCCGCCTTCAAGTGGTTCTACGCCCACCTCCCCGGCTTCGAGGGCTTCCGAGAGGGCACCAACGAGTCTCACATCATGAAGCTGGTGGCCCGACACCAGCTGACCCACCAGCTCACCCTGGTGACCGGCGCTGTGTCTGAGGAGTGCGCCCTGGTGCTGAAGGACGTGTACACCGACTCTCCCGAGTGGCACGACATCACCGCCAAGGACGCCAACATGAAGCTGATGGCTCGAATTACCTCTCGAGTGTTCCTGGGCAAGGAGATGTGCCGAAACCCCCAGTGGCTGCGAATCACCTCTACCTACGCCGTGATCGCCTTCCGAGCCGTCGAGGAGCTGCGACTGTGGCCCTCTTGGCTGCGACCCGTGGTCCAGTGGTTTATGCCCCACTGCACCCAGTCTCGAGCCCTGGTGCAGGAGGCCCGAGATCTGATCAACCCCCTGCTCGAGCGACGACGAGAGGAGAAGGCCGAGGCCGAGCGAACCGGCGAGAAGGTGACCTACAACGACGCCGTCGAGTGGCTGGACGACCTGGCCCGAGAGAAGGGCGTGGGCTACGACCCCGCCTGCGCCCAGCTGTCTCTGTCTGTGGCCGCCCTGCACTCTACCACCGACTTTTTCACCCAGGTGATGTTCGACATTGCCCAGAACCCCGAGCTGATCGAGCCCCTGCGAGAGGAGATCATTGCCGTCCTGGGCAAGCAGGGCTGGTCTAAGAACTCTCTGTACAACCTGAAGCTCATGGACTCTGTGCTGAAGGAGTCTCAGCGACTGAAGCCCATTGCCATTGCCTCTATGCGACGATTCACCACCCACAACGTCAAGCTGTCTGACGGCGTGATTCTGCCCAAGAACAAGCTGACCCTGGTGTCCGCCCACCAGCACTGGGACCCCGAGTACTACAAGGATCCCCTGAAGTTCGACGGCTACCGATTCTTCAACATGCGACGAGAGCCCGGCAAGGAGTCTAAGGCCCAGCTGGTGTCTGCCACCCCCGACCACATGGGCTTCGGCTACGGCCTGCACGCCTGTCCCGGCCGATTCTTCGCCTCTGAGGAGATCAAGATCGCCCTGTCTCACATCCTGCTGAAGTACGACTTCAAGCCCGTCGAGGGATCTTCTATGGAGCCCCGAAAGTACGGCCTGAACATGAACGCCAACCCCACCGCCAAGCTGTCTGTGCGACGACGAAAGGAGGAGATCGCCATCTGA

*GfP450-2* gene from *G. fujikuroi*

ATGTCCATCTTCAACATGATCACCTCTTACGCCGGCTCTCAGCTGCTGCCCTTCTACATTGCCATCTTCGTGTTCACCCTGGTGCCTTGGGCCATCCGATTCTCTTGGCTCGAGCTGCGAAAGGGCTCTGTGGTGCCCCTGGCTAACCCTCCTGACTCTCTGTTCGGCACCGGCAAGACCCGACGATCTTTCGTGAAGCTGTCTCGAGAGATCCTGGCCAAGGCTCGATCTCTGTTCCCCAACGAGCCCTTCCGACTGATCACCGACTGGGGCGAAGTGCTGATCCTGCCTCCTGATTTCGCCGACGAGATCCGAAACGACCCTCGACTGTCTTTCTCGAAGGCCGCCATGCAGGACAACCACGCCGGCATTCCCGGCTTCGAGACTGTGGCCCTGGTGGGCCGAGAGGACCAGCTGATCCAGAAGGTGGCCCGAAAGCAGCTGACCAAGCACCTGTCTGCCGTGATCGAGCCCCTGTCGCGAGAGTCTACCCTGGCCGTGTCTCTGAACTTCGGCGAGACTACCGAGTGGCGAGCCATCCGACTGAAGCCCGCCATCCTGGACATCATTGCCCGAATCTCTTCTCGAATCTACCTGGGCGACCAGCTGTGCCGAAACGAGGCCTGGCTGAAGATCACCAAGACCTACACCACCAACTTCTACACCGCCTCTACCAACCTGCGAATGTTCCCTCGATCTATTCGACCCCTGGCTCACTGGTTTCTGCCCGAGTGCCGAAAGCTGCGACAAGAGCGAAAGGACGCCATCGGCATCATCACCCCTCTGATCGAGCGACGACGAGAGCTGCGACGAGCCGCCATTGCCGCCGGACAGCCCCTGCCTGTGTTCCACGACGCCATTGACTGGTCTGAGCAAGAGGCCGAGGCCGCTGGAACCGGCGCCTCTTTCGACCCCGTGATCTTCCAGCTGACCCTGTCTCTGCTGGCCATCCACACCACCTACGACCTGCTCCAGCAGACCATGATCGACCTGGGACGACACCCCGAGTACATTGAGCCCCTCCGACAAGAGGTGGTGCAGCTGCTCCGAGAGGAAGGCTGGAAGAAGACCACTCTGTTCAAGATGAAGCTGCTGGACTCTGCCATCAAGGAATCTCAGCGAATGAAGCCCGGCTCTATCGTGACCATGCGACGATACGTGACCGAGGACATCACCCTGTCCTCTGGACTGACCCTGAAGAAGGGCACCCGACTGAACGTGGACAACCGACGACTGGACGACCCCAAGATCTACGACAACCCTGAGGTGTACAACCCCTACCGATTCTACGACATGCGATCTGAGGCCGGCAAGGACCACGGCGCCCAGCTGGTGTCTACCGGCTCTAACCACATGGGCTTCGGCCACGGCCAGCACTCTTGTCCCGGCCGATTCTTCGCCGCCAACGAGATCAAGGTCGCCCTGTGCCACATCCTGGTGAAGTACGACTGGAAGCTGTGCCCCGACACCGAGACTAAGCCCGACACTCGAGGCATGATCGCCAAGTCGTCGCCCGTGACCGACATTCTGATTAAGCGACGAGAGTCTGTCGAGCTGGACCTCGAGGCCATCTAA

*GfP450-3* gene from *G. fujikuroi*

ATGAAGTACACCACCTGTCAGATGAACATCTTCCCTTCTCTGTGGTCTATGAAGACCTCTTTCCGATGGCCTCGAACCTCTAAGTGGTCCTCTGTGTCTCTGTACGACATGATGCTGCGAACCGTGGCTCTGCTGTCTGGCCGAGCCTTCGTGGGACTGCCCCTGTGCCGAGATGAAGGCTGGCTGCAGGCCTCTATCGGCTACACCGTGCAGTGCGTGTCTATCCGAGATCAGCTGTTCACCTGGTCGCCCGTGCTGCGACCTATCATCGGCCCCTTTCTGCCCTCTGTGCGATCCGTGCGACGACACCTCCGATTCGCCGCCGAGATCATGGCCCCTCTGATCTCTCAGGCCCTGCAGGACGAGAAGCAGCACCGAGCTGACACCCTGCTGGCCGACCAGACCGAAGGCCGAGGCACCTTCATTTCTTGGCTGCTGCGACATCTGCCCGAGGAACTGCGAACCCCTGAGCAGGTCGGCCTGGACCAGATGCTGGTGTCTTTCGCCGCCATCCACACCACCACCATGGCTCTGACCAAGGTGGTCTGGGAGCTTGTGAAGCGACCCGAGTACATCGAGCCCCTCCGAACCGAGATGCAGGACGTGTTCGGCCCCGACGCCGTGTCTCCCGACATCTGTATCAACAAGGAAGCCCTGTCTCGACTGCACAAGCTGGACTCGTTCATCCGAGAGGTGCAGAGATGGTGCCCCTCTACCTTCGTGACCCCTTCGCGACGAGTGATGAAGTCTATGACCCTGTCTAACGGCATCAAGCTGCAGCGAGGAACCTCTATCGCTTTCCCCGCTCACGCCATTCACATGTCTGAGGAAACCCCTACCTTCTCTCCCGACTTCTCGTCTGACTTCGAGAACCCCTCTCCTCGAATCTTCGACGGCTTCCGATACCTGAACCTGCGATCTATCAAGGGCCAGGGCTCTCAGCACCAGGCCGCCACCACCGGACCTGACTACCTGATCTTCAACCACGGCAAGCACGCTTGTCCCGGCCGATTCTTCGCCATCTCTGAGATCAAGATGATCCTGATCGAGCTGCTGGCCAAGTACGACTTCCGACTCGAGGACGGCAAGCCCGGTCCTGAGCTGATGCGAGTGGGCACCGAGACTCGACTGGACACCAAGGCCGGACTCGAGATGCGACGACGATAA

*GfDes* gene from *G. fujikuroi*

ATGCCCCACAAGGACAACCTGCTCGAGTCTCCCGTGGGCAAGTCTGTGACCGCCACCATTGCCTACCACTCTGGCCCCGCTCTGCCCACCTCTCCTATCGCCGGCGTGACCACTCTGCAGGACTGCACCCAGCAGGCCGTGGCCGTGACCGACATTCGACCCTCTGTGTCCTCTTTCACCCTGGACGGCAACGGCTTCCAGGTGGTGAAGCACACCTCTGCCGTGGGCTCGCCTCCTTACGACCACTCTTCTTGGACTGACCCCGTGGTGCGAAAGGAAGTGTACGACCCCGAGATCATCGAGCTGGCCAAGTCGCTGACCGGCGCCAAGAAGGTGATGATCCTGCTGGCCTCTTCTCGAAACGTGCCCTTCAAGGAACCCGAGCTGGCTCCTCCTTATCCTATGCCTGGCAAGTCCTCTTCGGGCTCTAAGGAACGAGAGGCTATCCCCGCCAACGAGCTGCCCACCACTCGAGCCAAGGGCTTCCAGAAGGGCGAAGAGGAAGGCCCCGTTCGAAAGCCTCACAAGGACTGGGGCCCCTCTGGCGCCTGGAACACCCTGCGAAACTGGTCCCAAGAGCTGATCGACGAGGCCGGCGACATCATCAAGGCCGGTGACGAGGCTGCCAAGCTGCCCGGTGGCCGAGCCAAGAACTACCAGGGCCGACGATGGGCCCTGTACACCACTTGGCGACCCCTCAAGACCGTGAAGCGAGATCCCATGGCCTACGTGGACTACTGGACCGCCGACGAAGAGGATGGCGTGTCTTTCTGGCGAAACCCTCCTGGCGTGCACGGCACCTTCGAGTCTGACGTGCTGCTGACCAAGGCTAACCCCAAGCACAAGTGGTACTGGATCTCTGACCAGACTCCTGACGAGGTCCTGCTGATGAAGATCATGGACACCGAGTCTGAGAAGGACGGCTCTGAGATTGCCGGCGGAGTGCACCACTGTTCTTTCCATCTGCCTGGCACCGAGAAGGAAGAGGTGCGAGAGTCTATCGAGACTAAGTTCATTGCTTTCTGGTAA

*GfCyb5* gene from *G. fujikuroi*

ATGTCTGCCAAGAAGGAATTCACCATGCAGGACGTGGCCGAGCACAACACCTCTTCCGACATCTACATGGTGGTGCACGACAAGGTGTACGACTGTACCAAGTTCCTGGACGAGCACCCCGGTGGCGAAGAGGTGATGCTGGACGTCGCCGGCCAGGACGCCACCGAGGCCTTCGAGGACGTGGGCCACTCTGACGAGGCCCGAGAGGTGCTGGACGGCCTGCTGGTGGGCGAGCTGAAGCGACTGCCCGGCGACGAGGGCCCCAAGCGACAGATCGCCAACTCTAACCAGGGCTCTGGCAAGGCTGACCCCGCTGGATCTTCTCTGAACACCTACGCCATCGTGGTGGCCGTGGGCTTCATTGCCTACGTGGCCTACAACTACCTGCAGAAGCAGCAAGAGGCCCAGGGCCAAGCCTCTGCCTAA

*GfCybRed* gene from *G. fujikuroi*

ATGTCCTCTAACGGCGACAACCACTCTCTGTTCGCCCGACACTACATCGACTACGTGTACGCTCCCGGCCTGCTGCTGTGGGGCACCCTGATCGTGAAGAAGGAATGGGCTCCCTGGGCTCTGCTGGTGGCCGTGGTGTTCGGCATCTACAACTTCATGGCCTTCCAGGTCAAGACCACTCTGAAGCCCGACGTGTTCCAAGAGTTCGAGCTGGAAGAAAAGACCATCGTGTCTCACAACGTGGCCATCTACCGATTCAAGCTGCCCTCGCCTAAGCACATCCTGGGACTGCCCATCGGCCAGCACATCTCTATCGGCGCTCCCTGTCCTCAGCCTGACGGCACCACCAAGGAAATCGTGCGATCTTACACCCCTATCTCTGGCGACCACCAGCCTGGCCACGTGGACCTGCTGATCAAGTCTTACCCTCAGGGCAACATCTCCAAGCACATGGCTTCTCTGACCGTGGGCCAGACCATCAAGGTGCGAGGCCCCAAGGGCGCCTTCGTGTACACTCCCAACATGGTGCGACACTTCGGCATGATTGCCGGCGGAACCGGCATCACCCCTATGCTGCAGGTCATCCGAGCCATCGTGCGAGGACGAGCCGCTGGCGACAAGACCGAGGTGGACCTGATCTTCGCCAACGTGACCGCTCAGGACATCCTGCTGAAGGAAGATCTGGACGCCCTGGCCAAGCAGGACTCTGGCATCCGAGTGCACTACGTGCTGGACAAGCCCGAGGAAGGCTGGACCGGCGGAGTGGGCTACGTCACCGCCGACATGATCGACAAGTACCTGCCTAAGCCTGCCGACGACGTGAAGATCCTGCTGTGCGGCCCTCCTCCTATGATCTCTGGCCTGAAGAAGGCCACCGAGTCTCTGGGCTTCAAGAAGGCCCGACCTGTCTCTAAGCTGGTGGACCAGGTGTTCGCCTTCTAA

*GfCPR* gene from *G. fujikuroi*

ATGGCCGAGCTGGACACCCTGGACATCGTGGTGCTGGGCGTGATCTTCCTGGGCACCGTGGCCTACTTCACCAAGGGCAAGCTGTGGGGCGTGACTAAGGACCCCTACGCCAACGGCTTCGCCGCTGGCGGAGCCTCTAAGCCCGGACGAACCCGAAACATCGTCGAGGCCATGGAAGAGTCTGGCAAGAACTGCGTGGTGTTCTACGGCTCTCAGACCGGCACCGCCGAGGACTACGCCTCTCGACTGGCCAAGGAAGGCAAGTCTCGATTCGGCCTGAACACCATGATCGCCGACCTCGAGGATTACGACTTCGACAACCTGGACACTGTGCCCTCTGACAACATCGTGATGTTCGTGCTGGCCACCTACGGCGAGGGCGAGCCCACCGACAACGCCGTGGACTTCTACGAGTTCATTACCGGCGAGGACGCCTCTTTCAACGAGGGCAACGACCCTCCTCTGGGCAACCTGAACTACGTGGCCTTCGGCCTGGGCAACAACACCTACGAGCACTACAACTCTATGGTGCGAAACGTGAACAAGGCCCTCGAGAAGCTGGGCGCTCACCGAATCGGCGAGGCCGGCGAGGGTGACGACGGCGCTGGAACTATGGAAGAGGACTTCCTGGCCTGGAAGGACCCCATGTGGGAAGCCCTGGCCAAGAAGATGGGCCTCGAGGAACGAGAGGCCGTGTACGAGCCCATTTTCGCCATCAACGAGCGAGATGACCTGACTCCTGAGGCCAACGAGGTGTACCTGGGAGAGCCCAACAAGCTGCACCTCGAGGGCACCGCCAAGGGACCCTTCAACTCTCACAACCCCTACATTGCCCCTATCGCCGAGTCTTACGAGCTGTTCTCTGCCAAGGACCGAAACTGCCTGCACATGGAAATCGACATCTCTGGCTCTAACCTGAAGTACGAGACTGGCGACCACATTGCCATCTGGCCTACAAACCCCGGCGAAGAGGTGAACAAGTTCCTGGACATTCTGGACCTGTCTGGAAAGCAGCACTCTGTGGTGACCGTGAAGGCTCTCGAGCCCACTGCCAAGGTGCCCTTTCCAAACCCTACCACCTACGACGCCATCCTGCGATACCACCTCGAGATCTGCGCTCCCGTGTCTCGACAGTTCGTGTCTACCCTGGCCGCCTTCGCTCCCAACGACGACATCAAGGCCGAGATGAACCGACTGGGCTCTGACAAGGACTACTTCCACGAAAAGACCGGACCTCACTACTACAACATTGCCCGATTTCTGGCCTCTGTGTCTAAGGGCGAGAAGTGGACCAAGATTCCCTTCTCGGCTTTCATCGAGGGACTGACCAAGCTGCAGCCCCGGTACTACTCTATCTCTTCTTCGTCCCTGGTGCAGCCTAAGAAGATCTCTATCACCGCCGTGGTCGAGTCTCAGCAGATCCCCGGACGGGACGACCCCTTCCGAGGCGTGGCCACCAACTACCTGTTCGCCCTGAAGCAGAAGCAGAACGGCGACCCCAACCCTGCTCCTTTCGGCCAGAGCTACGAGCTGACTGGACCCCGAAACAAGTACGACGGCATTCACGTGCCCGTGCACGTCCGACACTCTAACTTCAAGCTGCCTTCTGACCCCGGCAAGCCCATCATCATGATCGGACCCGGCACCGGCGTGGCTCCCTTCAGAGGCTTCGTGCAAGAGCGAGCCAAGCAGGCCCGAGATGGTGTCGAGGTGGGCAAGACCCTGCTGTTCTTCGGCTGCCGAAAGTCTACCGAGGACTTCATGTACCAGAAGGAATGGCAAGAGTACAAGGAAGCTCTGGGCGACAAGTTCGAGATGATCACCGCCTTTTCGCGAGAGGGCTCTAAGAAGGTGTACGTCCAGCACCGACTGAAGGAACGATCTAAGGAAGTGTCTGACCTGCTGTCTCAAAAGGCTTACTTCTACGTGTGCGGCGACGCCGCTCACATGGCCCGAGAGGTCAACACCGTGCTGGCTCAGATCATTGCCGAAGGACGAGGCGTGTCCGAGGCCAAGGGCGAAGAGATCGTGAAGAACATGCGATCTGCCAACCAGTACCAGGTGTGCTCTGACTTCGTGACCCTGCACTGCAAGGAAACCACTTACGCCAACTCTGAGCTGCAAGAGGACGTGTGGTCTTAA

*SsGGPPs7* gene from *Synechococcus sp.*

ATGGTGGCCCAGACCTTCAACCTGGACACCTACCTGTCTCAGCGACAGCAGCAGGTCGAGGAAGCCCTGTCTGCCGCTCTGGTGCCTGCTTACCCCGAGCGAATCTACGAGGCCATGCGATACTCCCTGCTGGCTGGCGGCAAGCGACTGCGACCCATCCTGTGCCTGGCTGCCTGCGAGCTGGCCGGTGGCTCTGTCGAGCAGGCTATGCCCACCGCCTGCGCTCTCGAGATGATCCACACCATGTCTCTGATCCACGACGACCTGCCTGCCATGGACAACGACGACTTCCGACGAGGCAAGCCCACCAACCACAAGGTGTTCGGCGAGGACATTGCCATCCTGGCCGGCGACGCTCTGCTGGCCTACGCCTTCGAGCACATTGCCTCTCAGACCCGAGGCGTGCCTCCTCAGCTGGTGCTGCAGGTCATTGCCCGAATCGGACACGCCGTGGCCGCCACCGGACTGGTTGGCGGACAGGTGGTGGACCTCGAGTCCGAAGGCAAGGCCATCTCTCTGGAAACCCTCGAGTACATCCACTCTCACAAGACCGGCGCTCTGCTCGAGGCCTCTGTGGTGTCTGGCGGCATCCTGGCTGGTGCCGACGAGGAACTGCTGGCCCGACTGTCTCACTACGCCCGAGACATCGGCCTGGCCTTCCAGATCGTGGACGACATCCTGGACGTGACCGCCACCTCTGAGCAGCTGGGCAAGACCGCCGGCAAGGACCAGGCCGCTGCCAAGGCTACTTACCCCTCTCTGCTGGGCCTTGAGGCTTCTCGACAGAAGGCTGAGGAACTGATCCAGTCTGCCAAGGAAGCTCTGCGACCCTACGGCTCTCAGGCTGAGCCCCTGCTCGCCCTGGCCGACTTCATCACCCGACGACAGCACTAA

References:

Angerer, H. *et al.* (2014) ‘The LYR protein subunit NB4M/NDUFA6 of mitochondrial complex I anchors an acyl carrier protein and is essential for catalytic activity’, *Proceedings of the National Academy of Sciences*, 111(14), pp. 5207–5212. doi: 10.1073/pnas.1322438111.

Arnesen, J. A. *et al.* (2020) ‘Yarrowia lipolytica Strains Engineered for the Production of Terpenoids’, *Frontiers in Bioengineering and Biotechnology*, 8(August), pp. 1–14. doi: 10.3389/fbioe.2020.00945.

Holkenbrink, C. *et al.* (2018) ‘EasyCloneYALI: CRISPR/Cas9-Based Synthetic Toolbox for Engineering of the Yeast Yarrowia lipolytica’, *Biotechnology Journal*, 13(9), p. 1700543. doi: 10.1002/biot.201700543.
